# Emergent Lag Phase in Flux-Regulation Models of Bacterial Growth

**DOI:** 10.1007/s11538-023-01189-6

**Published:** 2023-08-14

**Authors:** Fiona Bate, Yumechris Amekan, Dmitri O. Pushkin, James P. J. Chong, Martin Bees

**Affiliations:** 1 Department of Mathematics, University of York, York, YO10 5DD UK; 2 Department of Biology, University of York, York, YO10 5DD UK

**Keywords:** Lag phase, Proteome partitioning, Flux-regulation, Mechanistic model, Diauxic growth, *Escherichia coli*, 92-10

## Abstract

Lag phase is observed in bacterial growth during a sudden change in conditions: growth is inhibited whilst cells adapt to the environment. Bi-phasic, or diauxic growth is commonly exhibited by many species. In the presence of two sugars, cells initially grow by consuming the preferred sugar then undergo a lag phase before resuming growth on the second. Biomass increase is characterised by a diauxic growth curve: exponential growth followed by a period of no growth before a second exponential growth. Recent literature lacks a complete dynamic description, artificially modelling lag phase and employing non-physical representations of precursor pools. Here, we formulate a rational mechanistic model based on flux-regulation/proteome partitioning with a finite precursor pool that reveals core mechanisms in a compact form. Unlike earlier systems, the characteristic dynamics emerge as part of the solution, including the lag phase. Focussing on growth of *Escherichia coli* on a glucose–lactose mixture we show results accurately reproduce experiments. We show that for a single strain of *E. coli*, diauxic growth leads to optimised biomass yields. However, intriguingly, for two competing strains diauxic growth is not always the best strategy. Our description can be generalised to model multiple different microorganisms and investigate competition between species/strains.

## Introduction

Microbial cells show four phases of growth: lag, log (exponential), stationary and death. Lag phase is observed when microorganisms are subject to a sudden change in conditions, such as the introduction of fresh growth media. During lag phase cells adapt to their new environment, synthesising the cellular components necessary for growth.

Diauxic growth, first described by Monod (1942, 1949), occurs when a microorganism is presented with two sugars that can be metabolised. The microorganism first consumes the preferred sugar until that source is almost completely exhausted, only then switching to consume the second food source (Monod 1949). There is a lag phase between the two phases of microbial growth on the different food sources which appears to be the result of a trade-off between rapid adaptation to changing growth conditions and supporting a high (and therefore competitive) growth rate (Chu and Barnes 2016). Diauxic growth can be interpreted as a way to maximise growth on two substrates (Kompala et al. 1984; Salvy and Hatzimanikatis 2021): the sequential use of substrates rather than the simultaneous consumption being beneficial under a wide range of conditions (Chu and Barnes 2016). However, the exact conditions are unclear for which diauxic growth performs better than other strategies, such as consuming both substrates at the same time, albeit at reduced efficiency; in a competitive environment where the two resources are limited, which strain grows most overall?

The underlying molecular interactions governing the response of a microorganism to a change in conditions are complex, although some important regulatory processes have been identified. For example, *E. coli* produces proteins to metabolise lactose only when lactose is present and glucose (the preferred carbon source) is absent. This is achieved through carbon catabolite repression (CCR) and inducer exclusion. CCR is one of the most significant regulatory processes in many bacteria, accounting for $5-10\%$ of all bacterial genes (Görke and Stülke 2008). In *E. coli*, CCR is mediated by the prevention of transcriptional activation of catabolic genes in the presence of glucose via the catabolite activator protein (CAP). CAP senses glucose indirectly through the ‘hunger signal’ molecule cyclic adenosine monophosphate (cAMP). Glucose depletion induces *E. coli* to produce more cAMP which binds to CAP, inducing a conformational change that results in binding to DNA, stimulating transcription of the genes involved in lactose metabolism.

The uptake of glucose inhibiting the ability of lactose permease to transport lactose into the cell is known as inducer exclusion (Aggarwal and Narang 2022). The uptake of glucose by the phosphotransferase system (PTS) is accompanied by the formation of the de-phosphorylated enzyme EIIA$^{\textrm{Glc}}$, which inactivates lactose permease by binding to it (Hogema et al. 1998).

The cooperative coordination of gene expression levels between these two regulatory mechanisms ensures that the preferred carbon source is used first, then metabolism is reconfigured to use the secondary carbon source.

Guanosine 3$^{\prime }$,5$^{\prime }$-bispyrophosphate (ppGpp), which down-regulates ribosome production and up-regulates amino acid biosynthesis genes, has been found to have an overarching role in coordination of gene expression during glucose–lactose diauxie (Traxler et al. 2006). The regulation of ribosome synthesis, via ppGpp, is determined by a balance between demand for and synthesis of amino acids. This amino acid flux has been identified as an important factor in the regulation of bacterial growth rate (Scott et al. 2014). cAMP, which is important in the regulation of metabolism as noted above, coordinates the expression of catabolic, biosynthetic and ribosomal proteins, ensuring that proteomic resources are spent on distinct metabolic sectors as required in different growth conditions (You et al. 2013).

The mechanisms responsible for reorganisation of gene expression (resource allocation) in microorganisms are generally believed to be optimised by evolution (Giordano et al. 2016). The optimum mechanism will depend on the growth environment. For example, in a non-competitive environment the maximisation of growth yield is thought to provide an advantage (Giordano et al. 2016) whereas when there is competition for resources, maximising growth rate will give a competitive advantage (Ibarra et al. 2002).

Recent theoretical studies on resource allocation have focussed on maximizing growth rate (Scott et al. 2014, 2010). Scott et al. (2014) used a coarse-grained model of the cell to show that maximum growth rate is acheived at a specific value of the ribosomal protein fraction through maximisation of the amino acid flux. The amount of protein in the cell was assumed constant and divided into related sectors (proteome partitioning): ribosomal proteins and metabolic proteins. Increasing the number of ribosomes therefore decreases metabolic enzyme levels. Their optimisation control strategy was based on the amino acid pool size, assumed to be signalled via ppGpp, controlling the fraction of total protein synthesis producing ribosomes (Scott et al. 2014). Similar models of resource allocation optimisation include energy constraints in addition to constraints on the proteome (Maitra and Dill 2015; Weiße et al. 2015).

The above studies involve steady state models, describing an environment that is stable over a long period of time. However, on the whole a microorganism is subject to a fluctuating range of growth conditions in its natural environment. This has motivated the formulation of dynamic resource allocation models (Salvy and Hatzimanikatis 2021; Giordano et al. 2016; Pavlov and Ehrenberg 2013; Erickson et al. 2017; Basan et al. 2020; Kremling et al. 2018). Kremling et al. (2018) present an ensemble of different models all showing diauxic behaviour. By qualitatively comparing model predictions they offer an insight into the variety of mechanisms that have been proposed to play a role in CCR. Basan et al. (2020) invesitgated shifts between two single carbon sources reporting that long lag phases are due to the depletion of key metabolites and resulting metabolic bottlenecks. Pre-shift growth rates were varied by using different carbon sources and their model of sequential flux limitation predicts a linear relationship between lag time and pre-shift growth rate. A stochastic simulation model presented by Chu and Barnes (2016) shows that it is impossible to shorten the lag phase without reducing the long term growth potential. Premature activation of the secondary metabolism shortens the lag but causes costs to the cell thus reducing the growth rate on the preferred substrate. They predict, using simulated evolution, that the lag phase will evolve to be longer in environments where switching is less likely to be required and shorter in frequently changing environments. Erickson et al. (2017) present a kinetic flux-controlled regulation model that quantitatively describes adaptation dynamics based on the dynamic reallocation of proteomic resources. The time evolution of gene expression is determined by regulation functions whose form is derived from steady-state growth laws. There are limitations on the validity of these regulation functions and in addition the model predicts constant proportionality between growth rate and substrate uptake rate, which is not observed experimentally during lag-phase growth.

In this study we extend and modify the model of Erickson et al. (2017) to include accurate prediction of biomass growth and substrate uptake during an initial lag-phase and during diauxic shift. We develop a coarse-grained model which uses qualitative knowledge of the molecular processes and a flux balance approach. We have avoided the potentially excessive complication of other models (Salvy and Hatzimanikatis 2021) explicitly so that we do not have large numbers of unmeasurable parameters. Unknown kinetic parameters in the model description are related to measurable kinetic parameters to minimise the need for fitting. Unlike many mathematical models describing lag-phase (Swinnen et al. 2004; Erickson et al. 2017), we do not introduce an artificial lag parameter to control the onset or length of the lag. Instead, the timing of the lag-phase is determined by substrate concentrations and the initial structure of the microorganism’s proteome.

We present a rational description, based on experimentally measurable parameters, which reproduces all principal features of the growth curve of *E. coli* during the switch from rich to minimal media and during glucose–lactose diauxie. Both the lag phase and log phase of bacterial growth emerge as part of the solution. Such a description (summarised in Sect. 2.3) can be used to demonstrate the relative merit of diauxic growth over the whole growth period and explore other growth strategies.

## Flux-Controlled Regulation of Anabolism and Catabolism

To model flux-controlled regulation (FCR) we shall adopt the modelling formalism of Erickson et al. (2017), develop a rational mathematical approach to address modelling inconsistencies and extend the description to describe physical aspects of precursor and amino acid pools.

### Original FCR Model

The FCR model due to Erickson et al. (2017) describes the time evolution of gene expression and biomass growth during carbon upshifts and downshifts. The model balances carbon influx and protein synthesis flux via changes to the average translation rate, $\sigma$, which is set by the size of a pool of central precursors including ketoacids and amino acids. Which proteins are produced (catabolic enzymes/ribosomes) is determined by regulation functions whose form is derived from steady-state growth laws. The central assumption of this model is that the time-dependence of the regulation functions during growth transitions depends solely on changes to the translation rate.

#### Limitations of the Original FCR Model

The regulation functions defined in Erickson et al. (2017) are undefined for a particular value of the translation rate, which we will call $\sigma _P$, and for $\sigma >\sigma _P$ the regulation functions incorrectly are negative. Although values of $\sigma \ge \sigma _P$ do not occur during steady-state growth they can occur during growth transitions. To remove this inconsistency and provide a firmer theoretical foundation we derive our regulation functions directly, associated with a mathematical optimization of the growth rate (see Sect. 2.2.5).

The original FCR model (Erickson et al. 2017) states that, on the time scale of interest, all fluxes are balanced. This balance is achieved by assuming that the translation rate adjusts abruptly with any changes to carbon influx (due to changes in substrate availability or the concentration of a key enzyme). However, for small values of the ribosome mass fraction or large carbon influx this can lead to large, physically unrealistic translation rates. We reason that as the translation rate depends on the size of the precursor pool, which is finite, the rate must be limited. Therefore, we shall include this limitation in our model (see Sect. 2.2.3).

Moreover, requiring flux balance in the above way results in the protein synthesis rate, and hence biomass growth rate, only depending on the catabolic protein mass fraction: the ribosome mass fraction drops out of the equations. The resulting constant proportionality between growth rate and substrate uptake (the constant biomass yield) predicted by the model of Erickson et al. (2017) does not agree with experimental observations. Our data, which we present in Sect. 3.2.1, shows that during an initial lag phase the ratio of growth rate to substrate uptake rate is significantly less than it is during the subsequent log-phase growth: the biomass yield is not constant. This suggests that growth is not being limited solely by the catabolic proteins, as this would also limit substrate uptake, but must depend on the levels of other key proteins.

#### Factors Limiting Growth During the Initial Lag Phase

Prior to the diauxie experiment (a full description of which is given in Sect. 3.2.1) *E. coli* was grown on Luria–Bertani broth (LB) which contains carbon sources and amino acids essential for growth. Cells of *E. coli* growing in LB can import amino acids directly and therefore do not need to use anabolic proteins to build amino acids. Indeed, it has been found experimentally that *E. coli* grown in LB show much lower levels of many genes involved in the amino acid biosynthetic pathways than those grown in minimal media (Tao et al. 1999). Therefore, we propose that the lag phase occurring when *E. coli* switches from growth on rich LB to minimal media is caused by a lack of the anabolic proteins needed for the biosynthesis of amino acids. To investigate this we extend the original FCR model to include an amino acid synthesis flux.

### Modified and Extended FCR Model

External substrates, $S_j$, are consumed by a microorganism, *X*. Inside the microbial cell catabolic enzymes break the substrate down into precursors. Anabolic proteins combine precursors to form amino acids that are subsequently incorporated by ribosomes into proteins required for growth. The relative amounts of the different enzymes and proteins required are determined by the growth conditions and substrates being consumed.

We construct a mathematical description of this process incorporating proteome partitioning, flux-controlled regulation and allocation of protein synthesis via optimisation of the growth rate.

#### Proteome Partitioning

Using an established model of proteome partitioning (Scott et al. 2010; Scott and Hwa 2011) we split the total protein content of the cell into different sectors, each composed of proteins whose expression levels show similar growth rate dependency in different growth conditions. The growth rate dependent sectors of the proteome are ribosome-affiliated proteins, *R*, enzymes relating to carbon import and metabolism, *C*, anabolic enzymes related to the production of amino acids, *A*, and an ‘uninduced’ sector, *U*, which generally decreases with decreasing growth rate (You et al. 2013). The rest of the proteome, *Q*, is growth rate independent and its mass fraction is non-zero and constant. It follows that

1 $$\begin{aligned} \Phi _R+\Phi _C+\Phi _A+\Phi _U+\Phi _Q=1, \end{aligned}$$

where $\Phi _i$ is the mass fraction of sector *i*. The minimum mass fraction of each sector, $\Phi _{i,0}$, is assumed to be growth rate independent (You et al. 2013) so that for each sector the growth rate dependent part is given by $\phi _i =\Phi _i-\Phi _{i,0}$. Thus, in terms of the growth rate dependent parts of the mass fractions, Eq. (1) becomes

$$\begin{aligned} \phi _R+\phi _A+\phi _C+\phi _U=\Phi _{\textrm{max}}, \end{aligned}$$

where $\Phi _{\textrm{max}}=1-\Phi _Q-\Phi _{R,0}-\Phi _{A,0}-\Phi _{C,0}-\Phi _{U,0}<1$ is a constant. This can be further simplified by noting that the uninduced sector of the proteome is found to be related to the ribosomal sector (under *C* and *A* limitation) such that $\phi _U=\varepsilon \phi _R$ (You et al. 2013). It follows that

2 $$\begin{aligned} (1+\varepsilon ) \phi _R+\phi _A+\phi _C=\Phi _{\textrm{max}}. \end{aligned}$$

During the log phase of growth of bacterial cells, the rate of cell proliferation (the growth rate) and the expression levels of key proteins are linearly correlated (You et al. 2013; Scott et al. 2010; Erickson et al. 2017). Each protein sector is assumed to be regulated as a whole (You et al. 2013; Hui et al. 2015) so $\phi _i$ is proportional to the expression level of a key protein in sector *i*, and thus to the growth rate. Denoting the value of the mass fraction during the log phase by $\phi _i^*$ it follows that

3 $$\begin{aligned} \phi _R^*=\frac{\lambda ^*}{\nu _R}, \phi _C^*=\Phi _{\textrm{max}}\left( 1-\frac{\lambda ^*}{\lambda _C}\right) , \phi _A^*=\frac{\lambda ^*}{\nu _A}, \end{aligned}$$

where $\lambda ^*$ is the constant growth rate of the *E. coli* cells in log phase and $\nu _R$, $\lambda _C$ and $\nu _A$ are constants (see Appendix A for further details).

#### Flux-Controlled Regulation

**Fig. 1:**
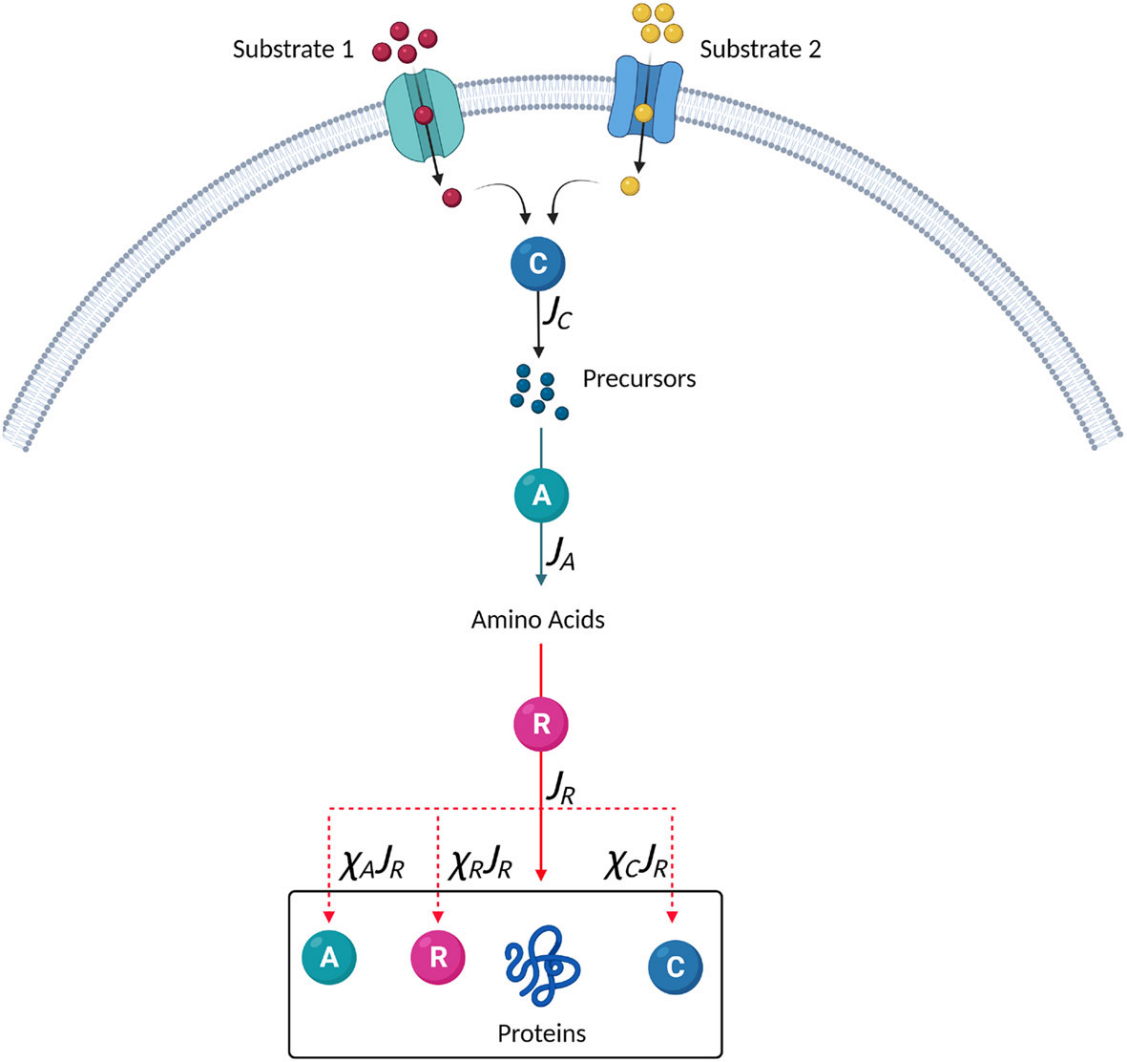
Flux-controlled regulation model. External substrates, $S_j$, are taken in and then broken down by catabolic enzymes, the C-sector, to supply a pool of carbon precursors. Changes in the concentration of the substrates and enzyme result in changes to the carbon influx, $J_C$. Other essential nutrients, including nitrogen, combine with these carbon precursors and are built up by anabolic proteins, the A-sector, to form amino acids. The flux of amino acid synthesis is given by $J_A$. A balance between $J_A$ and $J_C$ is achieved through changes to the average amino acid synthesis rate, $\sigma _C$, which in turn depends on the size of the precursor pool. The amino acids are “consumed” by ribosomes, the R-sector, in protein synthesis. The flux of protein synthesis is given by $J_R$. A balance between $J_R$ and $J_A$ is achieved through changes to the average translational activity, $\sigma _A$, which depends on the size of the amino acid pool. The regulation functions $\chi _R$, $\chi _A$ and $\chi _C$ determine the amount of ribosomal, anabolic and catabolic proteins, respectively, that are produced. Allocation of protein synthesis is regulated, via ppGpp and cAMP (Traxler et al. 2006; Scott et al. 2014; You et al. 2013), in response to changes to the precursor and amino acid pools. Under given growth conditions, there is an optimum level for each protein that will maximise the growth rate. During growth transitions the proteins are not at optimum levels, leading to changes in the precursor and amino acid pools and a non-optimum growth rate. In the model the regulation functions are derived directly, associated with a mathematical optimisation of the growth rate (Image created with BioRender.com) (Color figure online)

The core mechanisms represented in our model are shown in Fig. 1. The microorganism takes up substrates and breaks them down into carbon precursors. These precursors, together with other essential nutrients, combine to supply the cell with a pool of amino acids. The amino acids are utilised by ribosomes to produce proteins, *Z*. The rate of protein synthesis depends on the concentration of ribosomes, *R*, and the average translation rate, $\sigma _A$, so that $\textrm{d}Z/\textrm{d}t=\sigma _A R$. The total mass of protein as a fraction of total biomass is relatively constant (Erickson et al. 2017). Therefore, the total biomass concentration, *X*, is related to the total protein concentration by $Z=pX$, where the constant *p* is the fraction of biomass that is protein. It follows that

4 $$\begin{aligned} \frac{\textrm{d}X}{\textrm{d}t}=J_RX, \end{aligned}$$

where $J_R= \sigma _A\Phi _R$ represents the protein synthesis flux and $\Phi _R=R/(pX)$. Analogous to $J_R$ the amino acid synthesis flux is given by $J_A=\sigma _C {\bar{\Phi }}_{\mathcal {G}}$, where $\sigma _C$ is the average amino acid synthesis rate and ${\bar{\Phi }}_{\mathcal {G}}$ is the rescaled mass fraction of a key anabolic protein, $\mathcal {G}$. (We rescale $\Phi _\mathcal {G}$ with a factor proportional to $\Phi _{\textrm{max}}$ to remove an unknown constant from the equations, details are given in Appendix A.3). The relationship between ${\bar{\Phi }}_{\mathcal {G}}$ and the mass fraction of the total amino acid sector, $\Phi _A$, where $\Phi _A=A/(pX)$, is discussed in Sect. A.3 of the Appendix, with Eq. (A3) giving the explicit dependence.

The carbon influx rate, $J_C$, is proportional to the rate of substrate uptake. We base the substrate uptake equation on Michaelis–Menten kinetics (see Appendix B for details). For the case where there are *N* substrates, with concentrations $\{S_j\}=\{S_1,S_2,...S_N\}$, we have

5 $$\begin{aligned} \frac{\textrm{d}S_j}{\textrm{d}t}&=-k_{\textrm{max},j}\left( \frac{{\bar{\Phi }}_{E,j}}{{\bar{\Phi }}_{E,j}^*}\right) \frac{S_j}{K_{S,j}+S_j}X, \end{aligned}$$

where ${\bar{\Phi }}_{E,j}$ is the rescaled mass fraction of a substrate specific catabolic enzyme and ${\bar{\Phi }}_{E,j}^*$ is the value of that mass fraction during log-phase growth on the specific substrate. (As before, we rescale $\Phi _{E,j}$ to remove an unknown constant from the equations, details are given in Appendix A.2.) For non-repressed enzymes ${\bar{\Phi }}_{E,j}=\phi _C$, where $\phi _C=\Phi _C-\Phi _{C,0}$ and $\Phi _C=C/(pX)$. The constants $k_{\textrm{max},j}$ and $K_{S,j}$ are the maximum uptake rate and the Michaelis constant for substrate *j* respectively.

We define $Y_{C,j}$ to be the yield of carbon precursors from $S_j$, so, obtaining the substrate uptake rate from equation (5), it follows that the carbon influx rate due to substrate $S_j$ is given by

6 $$\begin{aligned} J_{C,j}=Y_{C,j}k_{\textrm{max},j}\left( \frac{{\bar{\Phi }}_{E,j}}{{\bar{\Phi }}_{E,j}^*}\right) \frac{S_j}{K_{S,j}+S_j}, \end{aligned}$$

with the total carbon flux $J_C=\sum \limits _{j=1}^NJ_{C,j}$. Note that we do not assume that $Y_{C,j}$ is constant, as is the case in Erickson et al. (2017), as this would result in the biomass yield, $Y_j$, being constant, which is inconsistent with experimental observations (as discussed in Sect. 2.1.1). In our model $Y_{C,j}$ and, therefore, the biomass yield, $Y_j$, depend on the growth conditions and proteome structure as we now show.

#### The Finite Precursor Pool Size

When growth conditions change, the amount of carbon available to enter the growth pathway (shown in Fig. 1), via the carbon influx, $J_C$, is affected. An abrupt upshift in substrate quality could cause $J_C$ to increase suddenly, resulting in a sudden increase in the production rate of carbon precursors. The level of the A-sector proteins cannot increase abruptly (as protein synthesis rates are proportional to the growth rate) and a bottleneck will occur in the growth pathway. This could be dealt with by abruptly increasing the amino acid synthesis rate, $\sigma _C$, as in Erickson et al. (2017), but accounting for large changes in $J_C$ in this way requires setting unrealistically high values for $\sigma _C$. Instead we note that the size of the precursor pool is limited by a cell’s capacity, there being only finite space within a cell. Thus the abundance of carbon precursors is limited which, as $\sigma _C$ depends on the abundance of carbon precursors, in turn limits the value of $\sigma _C$. (Similarly, the translational activity, $\sigma _A$, will have a maximum value.) To maintain flux balance we propose that the carbon entering the growth pathway, $J_C$, is limited. This is achieved by allowing the yield of carbon precursors, $Y_{C,j}$, to change as growth conditions change. Note that the substrate that is broken down but does not enter the growth pathway will be released as product (which we do not explicitly model). This is the case whether $Y_{C,j}$ is constant, as in Erickson et al. (2017), or changing, as in this model.

We let $P_{C,j}$ represent the concentration of carbon precursors added to the precursor pool by the flux $J_{C,j}$, defined in Eq. (6), and $P_{A,j}$ represent the amino acids subsequently synthesised from $P_{C,j}$. The combined size of the carbon precursor and amino acid pools can therefore be written as

7 $$\begin{aligned} P=\sum \limits _{j=1}^NP_{C,j}+\sum \limits _{j=1}^N\frac{P_{A,j}}{\alpha _{C,j}}, \end{aligned}$$

where $\alpha _{C,j}$ is a constant conversion factor from $P_{C,j}$ to $P_{A,j}$. There is a maximum value of *P* that can be maintained in the cell and we denote this by *K*. This constant, *K*, is analogous to the carrying capacity in population dynamics, the maximum population size that can be sustained in a given growth environment. In population dynamics the growth rate is limited by the carrying capacity, with growth tending to zero as the population size tends towards the carrying capacity. Here we limit the fluxes entering the carbon precursor pool so that $J_{C,j}\rightarrow 0$ as $P\rightarrow K$. We have

$$\begin{aligned} J_{C,j}=\left( \frac{K-P}{K}\right) J_{C,j,0}, \end{aligned}$$

where $J_{C,j,0}$ is the carbon flux from substrate *j* when $P=0$ given by

$$\begin{aligned} J_{C,j,0}=Y_{C,j,0}k_{\textrm{max},j}\left( \frac{{\bar{\Phi }}_{E,j}}{{\bar{\Phi }}_{E,j}^*}\right) \frac{S_j}{K_{S,j}+S_j}. \end{aligned}$$

The constant $Y_{C,j,0}$ is the yield of carbon precursors from $S_j$ when $P=0$. To simplify notation we introduce the function

8 $$\begin{aligned} f_j(\{S_j\})=\alpha _{A,j}\alpha _{C,j}Y_{C,j,0}k_{\textrm{max},j}\left( \frac{1}{{\bar{\Phi }}_{E,j}^*}\right) \frac{S_j}{K_{S,j}+S_j}, \end{aligned}$$

so that

9 $$\begin{aligned} J_{C,j}=\left( \frac{K-P}{K}\right) \left( \frac{1}{\alpha _{A,j}\alpha _{C,j}}\right) f_j{\bar{\Phi }}_{E,j}. \end{aligned}$$

To keep the number of variables in the model to a minimum we want the carbon influxes $J_{C,j}$ to be defined only in terms of the substrate concentrations and protein mass fractions. This means we need to know *P*, and therefore $P_{C,j}$ and $P_{A,j}$, only in terms of the substrate concentrations and protein mass fractions. This is done by considering flux balance.

The amino acid synthesis flux is given by $J_A=\sigma _C {\bar{\Phi }}_{\mathcal {G}}$, as discussed in Sect. 2.2.2, where $\sigma _C=\sigma _C(\{P_{C,j}\})$ depends on the abundance of carbon precursors. For simplicity, we take a linear dependence, setting ${\sigma _C=\sum _j\alpha _{C,j}k_{C,j}P_{C,j}}$, where $k_{C,j}$, the uptake rate of $P_{C,j}$, is a constant. The amino acid synthesis flux due to substrate *j* is therefore given by $J_{A,j}= \alpha _{C,j}k_{C,j}P_{C,j}{\bar{\Phi }}_{\mathcal {G}}$. Similarly, as the total protein synthesis flux $J_R= \sigma _A\Phi _R$, we take $\sigma _A=\sum _j\alpha _{A,j}k_{A,j}P_{A,j}$, where the constant $k_{A,j}$ is the uptake rate of $P_{A,j}$ and $\alpha _{A,j}$ is a constant conversion factor from $P_{A,j}$ to protein, and obtain the protein synthesis flux due to substrate *j* as $J_{R,j}= \alpha _{A,j}k_{A,j}P_{A,j}\Phi _R$.

The rates of change of $P_{C,j}$ and $P_{A,j}$ in terms of the fluxes, $J_{C,j}$, $J_{A,j}$ and $J_{R,j}$ are given by

$$\begin{aligned} \frac{\textrm{d}P_{C,j}}{\textrm{d}t}=J_{C,j}-\frac{1}{\alpha _{C,j}}J_{A,j},{} & {} \frac{\textrm{d}P_{A,j}}{\textrm{d}t}=J_{A,j}-\frac{1}{\alpha _{A,j}}J_{R,j}. \end{aligned}$$

To achieve flux balance, changes in $P_{C,j}$ and $P_{A,j}$ are assumed to take place over a relatively fast time scale, so that $\textrm{d}P_{C,j}/\textrm{d}t=\textrm{d}P_{A,j}/\textrm{d}t=0$. Essentially this means that on the timescale of interest all fluxes balance so that

10 $$\begin{aligned} J_{R,j}=\alpha _{A,j}J_{A,j},{} & {} J_{A,j}=\alpha _{C,j}J_{C,j}. \end{aligned}$$

Substituting for $J_{R,j}$, $J_{A,j}$ and $J_{C,j}$ in equations (10) we obtain a system of 2*N* equations in terms of $P_{C,j}$ and $P_{A,j}$. These can be solved to give $P_{C,j}$ and $P_{A,j}$ in terms of the substrate concentrations and protein mass fractions. From these we can then work out *P*, using equation (7), and substituting for *P* into (9) we obtain

11 $$\begin{aligned} J_{C,j}&=\frac{\displaystyle \left( \frac{f_j}{\alpha _{C,j}\alpha _{A,j}}\right) {\bar{\Phi }}_{\mathcal {G}}\Phi _R{\bar{\Phi }}_{E,j}}{\displaystyle {\bar{\Phi }}_{\mathcal {G}}\Phi _R+\left( \sum \limits _{n=1}^N\frac{f_n}{\sigma _{C\textrm{max},n}}{\bar{\Phi }}_{E,n}\right) \Phi _R+\left( \sum \limits _{n=1}^N\frac{f_n}{\sigma _{A\textrm{max},n}}{\bar{\Phi }}_{E,n}\right) {\bar{\Phi }}_{\mathcal {G}}}, \end{aligned}$$

where $\sigma _{A\textrm{max},n}=\alpha _{A,n}\alpha _{C,n}k_{A,n}K$ and $\sigma _{C\textrm{max},n}=\alpha _{A,n}\alpha _{C,n}k_{C,n}K$ are, respectively, the maximum translation rate and maximum amino acid synthesis rate when only substrate *n* is being consumed. Full details of the derivation of Eq. (11) are given in Appendix C.

Comparing Eq. (11) with the definition of $J_{C,j}$ given by Eq. (6), it follows that

12 $$\begin{aligned} Y_{C,j}=\frac{\displaystyle Y_{C,j,0}{\bar{\Phi }}_{\mathcal {G}}\Phi _R}{\displaystyle {\bar{\Phi }}_{\mathcal {G}}\Phi _R+\left( \sum \limits _{n=1}^N\frac{f_n}{\sigma _{C\textrm{max},n}}{\bar{\Phi }}_{E,n}\right) \Phi _R+\left( \sum \limits _{n=1}^N\frac{f_n}{\sigma _{A\textrm{max},n}}{\bar{\Phi }}_{E,n}\right) {\bar{\Phi }}_{\mathcal {G}}}. \end{aligned}$$

This equation describes how the yield of carbon precursors changes with the substrate concentrations (through $f_j$) and protein mass fractions.

We now use the expression we have derived for $J_{C,j}$, given by Eq. (11), and the flux balance equations (10) to derive an equation for biomass growth.

#### Biomass Growth

The equation for biomass growth in terms of the protein synthesis flux, $J_R=\sum \limits _{j=1}^NJ_{R,j}$, is given by equation (4). From flux balance we have $J_{R,j}= \alpha _{A,j}\alpha _{C,j}J_{C,j}$, with $J_{C,j}$ given by Eq. (11). It follows that

13 $$\begin{aligned} \frac{\textrm{d}X}{\textrm{d}t}=\frac{\displaystyle \left( \sum \limits _{n=1}^Nf_n{\bar{\Phi }}_{E,n}\right) {\bar{\Phi }}_{\mathcal {G}}\Phi _R}{\displaystyle {\bar{\Phi }}_{\mathcal {G}}\Phi _R+\left( \sum \limits _{n=1}^N\frac{f_n}{\sigma _{C\textrm{max},n}}{\bar{\Phi }}_{E,n}\right) \Phi _R+\left( \sum \limits _{n=1}^N\frac{f_n}{\sigma _{A\textrm{max},n}}{\bar{\Phi }}_{E,n}\right) {\bar{\Phi }}_{\mathcal {G}}}X, \end{aligned}$$

with $f_j$, given by Eq. (8). Note that the growth rate

14 $$\begin{aligned} \mu \left( =\frac{1}{X}\frac{\textrm{d}X}{\textrm{d}t}\right) =\frac{\displaystyle \left( \sum \limits _{n=1}^Nf_n{\bar{\Phi }}_{E,n}\right) {\bar{\Phi }}_{\mathcal {G}}\Phi _R}{\displaystyle {\bar{\Phi }}_{\mathcal {G}}\Phi _R+\left( \sum \limits _{n=1}^N\frac{f_n}{\sigma _{C\textrm{max},n}}{\bar{\Phi }}_{E,n}\right) \Phi _R+\left( \sum \limits _{n=1}^N\frac{f_n}{\sigma _{A\textrm{max},n}}{\bar{\Phi }}_{E,n}\right) {\bar{\Phi }}_{\mathcal {G}}}, \end{aligned}$$

is not directly proportional to the substrate uptake rate and depends on the mass fractions of each of the growth dependent proteome sectors. For small $\Phi _R$ the growth rate is limited by $\Phi _R$ and similarly for ${\bar{\Phi }}_{\mathcal {G}}$ and $\phi _C$ (through ${\bar{\Phi }}_{E,j}$). Crucially, for fixed substrate concentration (constant $f_j$), the growth rate $\mu =\mu ({\bar{\Phi }}_{\mathcal {G}},\Phi _R,\phi _C)$ has a unique maximum value at specific values of ${\bar{\Phi }}_{\mathcal {G}}$, $\Phi _R$ and $\phi _C$. We hypothesise that during the log-phase cells grow at this optimal rate. This hypothesis uniquely defines the values of the unknown constants in Eqs. (13) and (14), $\sigma _{C\textrm{max},j}$, $\sigma _{A\textrm{max},j}$ and $\alpha _{A,j}\alpha _{C,j}Y_{C,j,0}$ (this latter combination of constants appears in the definition of $f_j$). In terms of experimentally measurable parameters we find

15 $$\begin{aligned} \sigma _{A\textrm{max},j}&= \frac{\left( \Phi _{\textrm{max}}+(1+\varepsilon )\Phi _{R,0}+{\bar{\Phi }}_{\mathcal {G},0}\right) Y_{j}^*k_{\textrm{max},j}}{(1+\varepsilon )\Phi _{R,j}^{*2}}, \end{aligned}$$

16 $$\begin{aligned} \sigma _{C\textrm{max},j}&= \frac{\left( \Phi _{\textrm{max}}+(1+\varepsilon )\Phi _{R,0}+{\bar{\Phi }}_{\mathcal {G},0}\right) Y_{j}^*k_{\textrm{max},j}}{{\bar{\Phi }}_{\mathcal {G},j}^{*2}}, \end{aligned}$$

17 $$\begin{aligned} f_j(\{S_j\})&= \left( \Phi _{\textrm{max}}+(1+\varepsilon )\Phi _{R,0}+{\bar{\Phi }}_{\mathcal {G},0}\right) Y_{j}^*k_{\textrm{max},j}\left( \frac{1}{{\bar{\Phi }}_{E,j}^*}\right) ^2\frac{S_j}{K_{S,j}+S_j}, \end{aligned}$$

where the constant $Y_j^*$ is the measured biomass yield during log-phase growth on substrate *j*. Full details are given in Appendix D.

#### Allocation of the Protein Synthesis Flux

Allocation of protein synthesis is regulated, via ppGpp and cAMP, in response to changes in the precursor and amino acid pools (Traxler et al. 2006; Scott et al. 2014; You et al. 2013): protein levels are adjusted until pool sizes are optimal. Variations in pool sizes manifest as changes to the growth rate (the size of precursor and amino acid pools being explicitly included in the derivation of the growth rate equation (14), see Sect. 2.2.3) and it follows that when precursor and amino acid pool sizes are optimal the growth rate is maximal. We therefore allocate the protein synthesis flux through regulation functions that adjust the level of proteins until the growth rate is maximal.

Proteins belonging to the growth independent sector of the proteome, *Q*, are produced as a constant fraction, $\Phi _{Q}$, of total protein production. Proteins belonging to the growth dependent proteome sectors are produced in varying amounts depending on the current state of the proteome and the growth conditions. The *R*, *A* and *C* sectors of the proteome are each composed of a growth independent part and a growth dependent part. The growth independent parts are produced at a constant fraction of total protein produced as for sector *Q*. For the growth dependent parts we define regulation functions $\chi _R$, $\chi _C$ and $\chi _A$ to represent the fraction of the total amount of protein produced that is R-sector, C-sector, and A-sector protein respectively so that

18a $$\begin{aligned} \frac{\textrm{d}R}{\textrm{d}t}&= \left( \Phi _{R,0}+\chi _R\right) \frac{\textrm{d}Z}{\textrm{d}t}, \end{aligned}$$

18b $$\begin{aligned} \frac{\textrm{d}C}{\textrm{d}t}&= \left( \Phi _{C,0}+\chi _C\right) \frac{\textrm{d}Z}{\textrm{d}t}, \end{aligned}$$

18c $$\begin{aligned} \frac{\textrm{d}A}{\textrm{d}t}&= \left( \Phi _{A,0}+\chi _A\right) \frac{\textrm{d}Z}{\textrm{d}t}. \end{aligned}$$

As the total amount of protein $Z=pX$ the regulation functions are constrained by

19 $$\begin{aligned} (1+\varepsilon )\chi _R+\chi _C+\chi _A=\Phi _{\textrm{max}}, \end{aligned}$$

and we also require $\chi _R\ge 0$, $\chi _C\ge 0$ and $\chi _A\ge 0$ as protein is not recycled or destroyed (this is a simplifying assumption of our model). Using the relationships between biomass concentration and total protein concentration, and protein concentrations and protein mass fractions, given in Sect. 2.2.2, we rewrite Eq. (18) in terms of the growth dependent protein mass fractions (details given in Appendix E). We have

20a $$\begin{aligned} \frac{\textrm{d}\phi _R}{\textrm{d}t}&= \left( \chi _R-\phi _R\right) \mu , \end{aligned}$$

20b $$\begin{aligned} \frac{\textrm{d}\phi _C}{\textrm{d}t}&= \left( \chi _C-\phi _C\right) \mu , \end{aligned}$$

20c $$\begin{aligned} \frac{\textrm{d}\phi _A}{\textrm{d}t}&= \left( \chi _A-\phi _A\right) \mu , \end{aligned}$$

with the growth rate, $\mu$, given by Eq. (14). Substrate specific enzymes belong to the *C* sector and their regulation therefore depends on $\chi _C$. We set

21 $$\begin{aligned} \frac{\textrm{d}{\bar{\phi }}_{E,j}}{\textrm{d}t}&= \left( \chi _{E,j}-{\bar{\phi }}_{E_j}\right) \mu , \end{aligned}$$

where $\chi _{E,j}$ is the regulation function for the substrate specific enzyme. For enzymes that are always expressed proportional to the whole *C* sector we have $\chi _{E,j}=\chi _C$, however, if an enzyme is repressed under certain conditions this is not the case. Here we set

22 $$\begin{aligned} \chi _{E,j}&= \eta _j\left( \zeta _j\Phi _{\textrm{max}}+\chi _C\left( 1-\zeta _j\right) \right) , \end{aligned}$$

where the function $\zeta _j({\bar{\phi }}_{E,j},\phi _C)$ switches $\chi _{E,j}$ from its maximum level, $\chi _{E,j}=\Phi _{\textrm{max}}$, for ${\bar{\phi }}_{E,j}\ll \phi _C$ to $\chi _{E,j}=\chi _C$ for ${\bar{\phi }}_{E,j}\sim \phi _C$. The choice of

23 $$\begin{aligned} \zeta _j=\frac{1}{2}\left( 1-\tanh \left( \frac{1}{\epsilon }\left( \frac{{\bar{\phi }}_{E,j}}{\phi _C}-\frac{1}{2}\right) \right) \right) , \end{aligned}$$

where $\epsilon \ll 1$, facilitates such a switch smoothly (for ${\bar{\phi }}_{E,j}\ll \phi _C$ we have $\zeta _j\rightarrow 1$ and for ${\bar{\phi }}_{E,j}\sim \phi _C$ we have $\zeta _j\rightarrow 0$). The function $\eta _j(\{S_j\})$, $0\le \eta _j\le 1$, depends on which substrates are present in the system and determines whether a substrate specific enzyme is being expressed. For example, when modelling glucose–lactose diauxie, the glucose specific enzyme will always be expressed so we set $\eta _{\textrm{gl}}=1$ (note also that as ${\bar{\phi }}_{E,j}=\phi _C$ when an enzyme is always expressed we have $\zeta _{\textrm{gl}}=0$ and hence $\chi _{E,\textrm{gl}}=\chi _C$). However, the lactose specific enzyme is only expressed when the concentration of glucose drops sufficiently. The point at which lactose uptake switches on is not very well defined but we require $\eta _{\textrm{la}}\ll 1$ when the glucose concentration, $S_{\textrm{gl}}$, is large and $\eta _{\textrm{la}}\rightarrow 1$ as $S_{\textrm{gl}}\rightarrow 0$. This can be modelled by setting

24 $$\begin{aligned} \eta _{\textrm{la}}=\frac{K_L^2+\xi S_{\textrm{gl}}^2}{K_L^2+S_{\textrm{gl}}^2}, \end{aligned}$$

where $K_L$ is a constant and $\xi \ll 1$ gives the level of lactose specific enzyme expression in the presence of glucose (the pre-expression level). This choice of function enables a smooth transition between repressing lactose uptake when glucose concentrations are high and no repression of lactose uptake at zero glucose concentration. Glucose levels must drop so that $S_{\textrm{gl}}\approx K_L$ before the lactose specific enzyme is fully expressed. A similar functional form is used in Okano et al. (2020) to model enzyme regulation in the hierarchical use of substrates by *E. coli*.

As stated above, we require the regulation functions in our model to adjust the level of proteins until, for the given conditions i.e. substrate concentrations, the growth rate is maximised. To derive the regulation functions we make use of the standard calculus result that the greatest rate of increase of a function at a given point is in the direction given by the gradient of that function at that point. As the regulation functions are constrained we set

25a $$\begin{aligned} \chi _R&= \phi _R+\frac{C(\{S_j\})}{1+\varepsilon }\left( \left( \frac{1}{1+\varepsilon }\right) \frac{\partial \mu }{\partial \phi _R}-\gamma (\{S_j\})\frac{\partial \mu }{\partial \phi _C}-(1-\gamma (\{S_j\}))\frac{\partial \mu }{\partial \phi _A}\right) , \end{aligned}$$

25b $$\begin{aligned} \chi _C&= \phi _C+C(\{S_j\}) \left( \frac{\partial \mu }{\partial \phi _C}-\gamma (\{S_j\})\frac{\partial \mu }{\partial \phi _A}-(1-\gamma (\{S_j\}))\left( \frac{1}{1+\varepsilon }\right) \frac{\partial \mu }{\partial \phi _R}\right) , \end{aligned}$$

25c $$\begin{aligned} \chi _A&= \phi _A+C(\{S_j\}) \left( \frac{\partial \mu }{\partial \phi _A}-(1-\gamma (\{S_j\}))\frac{\partial \mu }{\partial \phi _C}-\gamma (\{S_j\})\left( \frac{1}{1+\varepsilon }\right) \frac{\partial \mu }{\partial \phi _R}\right) , \end{aligned}$$

which satisfies Eq. (19). The function $C(\{S_j\})$ is chosen to ensure that $(1+\varepsilon )\chi _R$, $\chi _C$ and $\chi _A$ never individually exceed $\Phi _{\textrm{max}}$, and $\gamma (\{S_j\})$ is chosen to ensure that $\chi _R\ge 0$, $\chi _C\ge 0$ and $\chi _A\ge 0$. Details of the derivation of $C(\{S_j\})$ and $\gamma (\{S_j\})$ are given in Appendix F.

These regulation functions are a mathematical representation of protein synthesis regulation via ppGpp and cAMP: protein levels are adjusted to optimise growth rate in a given environment. The regulation functions are never negative and the constraint on protein production, given by Eq. (19), is always satisfied.

From here on we will not solve explicitly for $\phi _A$ as its value is determined from $\phi _R$ and $\phi _C$ using equation (2).

### Governing Equations

In summary, we have constructed a mechanistic model describing the time evolution of biomass growth, substrate concentration and gene expression during carbon upshifts and downshifts. Phases of microorganism growth emerge from the dynamics of the proteome, rather than being switched on/off at a particular time. The model incorporates proteome partitioning, flux-controlled regulation and optimal allocation of protein synthesis. The governing equations are

26a $$\begin{aligned} \frac{\textrm{d}S_j}{\textrm{d}t}&= -k_{\textrm{max},j}\left( \frac{{\bar{\Phi }}_{E,j}}{{\bar{\Phi }}_{E,j}^*}\right) \frac{S_j}{K_{S,j}+S_j}X, \end{aligned}$$

26b $$\begin{aligned} \frac{\textrm{d}\phi _R}{\textrm{d}t}&= \left( \chi _R-\phi _R\right) \mu , \end{aligned}$$

26c $$\begin{aligned} \frac{\textrm{d}{\bar{\phi }}_{E,j}}{\textrm{d}t}&= \left( \chi _{E,j}-{\bar{\phi }}_{E_j}\right) \mu , \end{aligned}$$

26d $$\begin{aligned} \frac{\textrm{d}X}{\textrm{d}t}&= \mu X, \end{aligned}$$

with the growth rate, $\mu =\mu (\{S_j\},\Phi _R,\{{\bar{\Phi }}_{E,j}\})$, given by equation (14). An overview of the model variables and parameters is given in Tables 1 and 2.

**Table 1:** Overview of model variables

Variable		Description
$S_j$		Concentration of $j{\textrm{th}}$ substrate
*X*		Concentration of biomass
$\phi _R$		Growth-dependent mass fraction of R-sector proteins
$\phi _C$		Growth-dependent mass fraction of C-sector proteins
$\phi _A$	$=\Phi _{\textrm{max}}-(1+\varepsilon )\phi _R-\phi _C$	Growth-dependent mass fraction of A-sector proteins
$\Phi _R$	$=\Phi _{R,0}+\phi _R$	Total mass fraction of R-sector proteins
${\bar{\Phi }}_{E,j}$		Total mass fraction of substrate specific C-sector protein
${\bar{\Phi }}_{\mathcal {G}}$	$={\bar{\Phi }}_{\mathcal {G},0}+\phi _A$	Total mass fraction of key A-sector protein
$f_j(\{S_j\})$	Defined in Eq. (17)	Substrate dependent function
$\mu$	Defined in Eq. (14)	Growth rate
$\chi _R$	Defined in Eq. (25a)	Fraction of growth-dependent protein produced that is R-sector
$\chi _C$	Defined in Eq. (25b)	Fraction of growth-dependent protein produced that is C-sector
$\chi _{E,j}$	Defined in Eq. (22)	Regulation function for substrate specific catabolic enzyme
$\zeta _j({\bar{\phi }}_{E,j},\phi _C)$	Defined in Eq. (23)	Function determining whether substrate specific enzyme is expressed
$\eta _j(\{S_j\})$	Example given in Eq. (24)	Function determining whether substrate specific enzyme is expressed
$C(\{S_j\})$	Defined in Appendix F	Ensures that $(1+\varepsilon )\chi _R$ and $\chi _C$ do not exceed $\Phi _{\textrm{max}}$
$\gamma (\{S_j\})$	Defined in Appendix F	Ensures that $\chi _R$ and $\chi _C$ are positive

A full description and derivation of variables is given in the text

**Table 2:** Overview of model parameters

Parameter		Description
$Y_j^*$		Biomass yield measured during log-phase growth on substrate $S_j$
$k_{\textrm{max},j}$		Maximum uptake rate of $S_j$
$K_{S,j}$		Half saturation constant on $S_j$
$\nu _R$		Fitted parameter in growth law
$\lambda _C$		Fitted parameter in growth law
$\nu _A$	$= \left( \frac{\Phi _{\textrm{max}}}{\lambda _C}-\frac{(1+\varepsilon )}{\nu _R}\right) ^{-1}$	Fitted parameter in growth law
$\Phi _{\textrm{max}}$		Total combined mass fraction of growth dependent proteins
$\varepsilon$		Constant relating uninduced sector to R-sector
$\phi _{R,j}^*$	$=\frac{\displaystyle Y_j^*k_{\textrm{max},j}}{\displaystyle \nu _R}$	Value of $\phi _R$ during log-phase growth on substrate $S_j$
$\phi _{C,j}^*$	$=\Phi _{\textrm{max}}\left( 1-\frac{\displaystyle Y_j^*k_{\textrm{max},j}}{\displaystyle \lambda _C}\right)$	Value of $\phi _C$ during log-phase growth on substrate $S_j$
$\phi _{A,j}^*$	$=\frac{\displaystyle Y_j^*k_{\textrm{max},j}}{\displaystyle \nu _A}$	Value of $\phi _A$ during log-phase growth on substrate $S_j$
$\Phi _{R,0}$		Minimum ribosomal mass fraction
$\Phi _{\mathcal {G},0}$		Minimum mass fraction of key A-sector protein
$\sigma _{A\textrm{max},j}$	$=\frac{\displaystyle \left( \Phi _{\textrm{max}}+(1+\varepsilon )\Phi _{R,0}+{\bar{\Phi }}_{\mathcal {G},0}\right) Y_{j}^*k_{\textrm{max},j}}{\displaystyle (1+\varepsilon )\Phi _{R,j}^{*2}}$	Maximum translation rate when consuming $S_j$
$\sigma _{C\textrm{max},j}$	$=\frac{\displaystyle \left( \Phi _{\textrm{max}}+(1+\varepsilon )\Phi _{R,0}+{\bar{\Phi }}_{\mathcal {G},0}\right) Y_{j}^*k_{\textrm{max},j}}{\displaystyle {\bar{\Phi }}_{\mathcal {G},j}^{*2}}$	Maximum amino acid synthesis rate when consuming $S_j$

A full description and derivation of parameters is given in the text

The exact mechanism underlying the inhibition of substrate uptake is not made explicit in the model, making it flexible and applicable to many processes. In addition, the description can be generalised to model multiple different microorganisms, facilitating investigation of competition between different species or strains.

The model can be applied to describe lag phases caused by a lack of ribosomal proteins, anaobolic proteins or catabolic proteins. Indeed, unlike previously published models (Erickson et al. 2017; Salvy and Hatzimanikatis 2021; Wu et al. 2023), this model can capture more complex systems where there are sequential lag phases, for example an initial lag due to a change from rich to minimal growth media (lack of anabolic proteins) followed by a diauxic shift (lack of specific catabolic protein).

We now apply our model to the particular case of *E. coli* growing on a glucose–lactose mixture, comparing numerical results to data from the literature and preliminary experimental data.

## Results

We first parameterize and test our mathematical model using the *E. coli* glucose–lactose diauxie experimental data from Erickson et al. (2017). We then test the model against our own experimental data, which in addition to the glucose–lactose diauxic shift includes an initial lag due to a change from rich to minimal growth media.

**Fig. 2:**
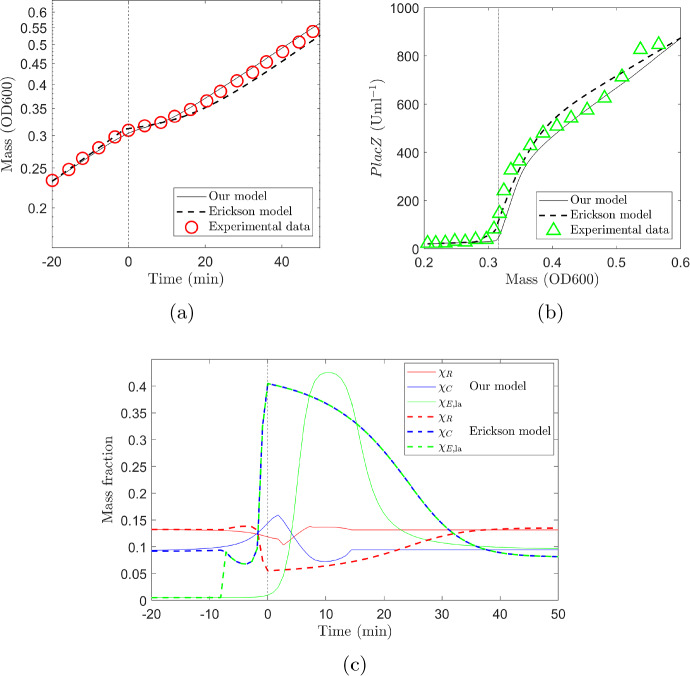
Comparison of simulation results using our model and the model of Erickson et al. (2017) with experimental data taken from Figure 3 of Erickson et al. (2017). **a** Growth curves of *E. coli* grown on $0.03\%$ glucose and $0.2\%$ lactose. The solid black line shows results of our model, the dashed black line shows results using the Erickson model (Erickson et al. 2017) and red circles show experimental data. **b** Expression level of lactose specific catabolic enzyme *PlacZ*. The solid black line shows results of our model, the dashed black line shows results using the Erickson model (Erickson et al. 2017) and green triangles show experimental data. **c** Comparison of regulation functions in the two models. Solid lines show this model and dashed lines the Erickson model. The main difference between the models is in the regulation of the *C* sector at the point of diauxic shift. In our model the large upscaling of enzyme production affects only the lactose specific enzyme whereas in the Erickson model the whole *C* sector is upregulated. Vertical dashed lines indicate the diauxic shift (Color figure online)

### Simulation 1: Modelling a Single Lag Phase

We simulated the diauxic shift of *E. coli* growing on a mixture of glucose and lactose and compared our results to experimental data from Erickson et al. (2017). Full details of the governing equations and parameters used in the numerical simulations are given in Appendix G. The equations were solved in Matlab (MATLAB 2020) using the solver ode15s.

Figure 2 shows simulation results from our model, simulation results we have reproduced using the model of Erickson et al. (2017) and experimental data taken from Figure 3 of Erickson et al. (2017). Figure 2a shows growth curves of *E. coli* grown on $0.03\%$ glucose and $0.2\%$ lactose. Our model results are shown as the solid black line, the dashed black line shows results using the Erickson model (Erickson et al. 2017) and red circles show experimental data. There is a discrepancy between the results of the Erickson model we have reproduced here and those shown in Figure 3 of Erickson et al. (2017). This is due to the miscalculation of a scaling factor in the original work that we have corrected (the predicted response immediately after shift quoted in the caption to Figure 3 in Erickson et al. (2017) as 7866 Uml$^{-1}$OD600 should be 8280Uml$^{-1}$OD600). Figure 2b shows the expression level of lactose specific catabolic enzyme *PlacZ*. The solid black line shows results of our model, the dashed black line shows results using the Erickson model (Erickson et al. 2017) and green triangles show experimental data. The expression of *PlacZ* is repressed in the presence of glucose but then increases rapidly on depletion of glucose, which occurs at $t=0$ shown by the vertical dashed line. A comparison of regulation functions in the two models is shown in Fig. 2c. Solid lines show the regulation functions for our model and dashed lines for the Erickson model with $\chi _R$ (red), $\chi _C$ (blue) and $\chi _{E,\textrm{la}}$ (green). The main difference between the models is in the regulation of the *C* sector at the point of diauxic shift. In our model the large upscaling of enzyme production affects only the lactose specific enzyme whereas in the Erickson model the whole *C* sector is upregulated.

Results show that both models reproduce the growth curve and enzyme expression level well with our model fitting slightly better to the growth curve data (residual sum of squares (RSS) $1.6\times 10^{-3}$ our model, $3.8\times 10^{-3}$ Erickson model) and Erickson model fitting slightly better to the *PlacZ* expression level (RSS $1.2\times 10^{-3}$ our model and $4.1\times 10^{-4}$ Erickson model).

### Simulation 2: Modelling Two Sequential Lag Phases

We now use our model to describe a more complex experimental system where there are two sequential lag phases: an initial lag due to a change from rich to minimal growth media followed by a diauxic shift. To accurately predict the time evolution of biomass and substrate concentrations in such a system a model must include multiple proteome sectors and a variable biomass yield. These features are included in our model unlike previously published models (Pavlov and Ehrenberg 2013; Erickson et al. 2017; Salvy and Hatzimanikatis 2021) which are, therefore, unable to model this system.

#### Methods

**Fig. 3:**
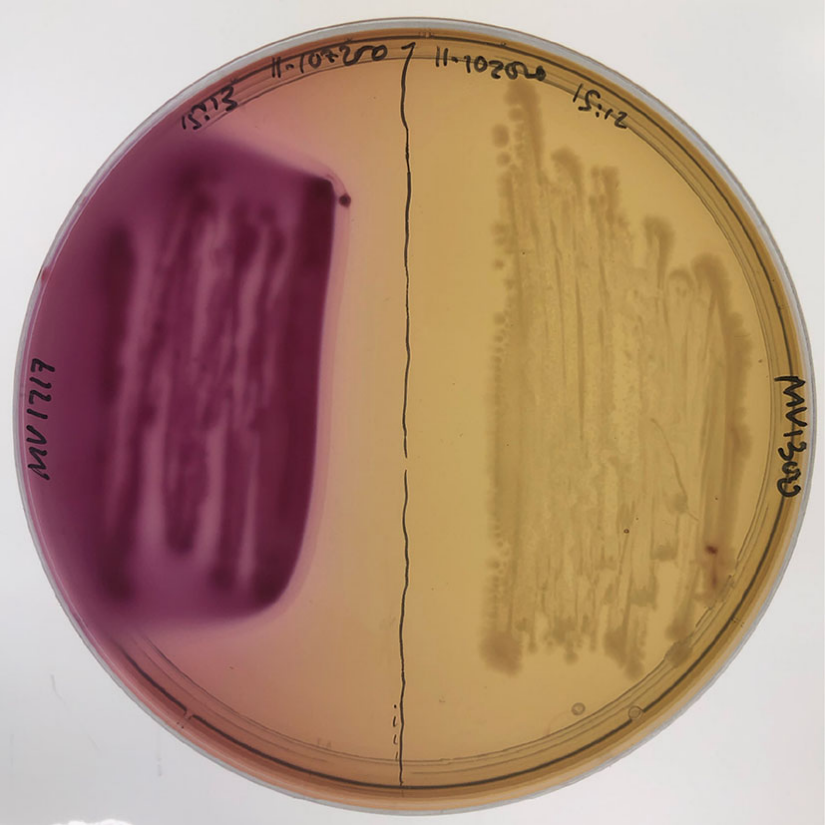
The two utilised *E. coli* strains growing on differential MacConkey agar. Lactose fermenters grow red or pink, cells unable to utilise lactose do not change colour. Left: MV1717 a lactose fermenting (*lac*$^{+}$) strain. Right: MV1300 a non-lactose utilising (*lac*$^{-}$) strain (Color figure online)

We recreated the glucose–lactose diauxie experiment of Mostovenko et al. (2011), using mixed *E. coli* strains. The following two strains were employed: *E. coli* MV1717 (MG1655 *lac*$^{+}$ containing chromosome-encoded, inducible CDI-msfGfp, chloramphenicol (Cm) resistance) and *E. coli* MV1300 (MG1655 delta lacZYA; kanamycin (Kan) resistance). Strain MV1717 can grow on lactose (*lac*$^{+}$), while MV1300 cannot utilise lactose (*lac*$^{-}$) as it is missing the lacY gene that encodes lactose permease, a membrane transporter that pumps lactose into cells. This characteristic was confirmed by growth on MacConkey agar, as shown in Fig. 3, where MV1717 (*lac*$^{+}$) colonies grow pink and MV1300 (*lac*$^{-}$) colonies grow colourless (white).

Strains were grown separately overnight on Luria–Bertani agar (LB-agar Miller; LMM0204, Formedium, Hunstanton, UK) at 37°C. A single colony of each strain was then grown overnight in 50 mL Falcon tubes containing 25 mL LB medium (LB broth, Miller; BP9723-500, Fisher BioReagents, Loughborough, UK) at 37°C with 120 rpm orbital shaking. Both the LB-agar and LB broth contained antibiotics: 30 $\upmu$g/mL Kan for MV1300 and 34 $\upmu$g/mL Cm for MV1717 respectively. The use of antibiotics was necessary for the qPCR we carried out which targeted plasmids carried by these strains that were used for quantification.

Biomass was measured using an established OD vs cell density relationship for *E. coli* (Brown 2010). (Note that whilst OD will likely depend on the geometry of cells, granularity and other aspects, it is commonly assumed to be proportional to biomass.) When the optical density at 600 nm (OD600) reached 1.4 ($\sim 1.1\times 10^9$ cells/mL), the biomass from each tube was harvested via centrifugation (Centrifuge 5810 R, Eppendorf) at $1940\times$g, 37°C. Supernatants were removed and the pellet was resuspended in 10 mL of warm (37°C) filter sterile 1x phosphate buffered saline (PBS 20-7400-10, Severn Biotech Ltd.). The biomass was spun again with the same parameters. The PBS was removed and the pellet was resuspended in 10 mL of 1x Morpholinepropanesulfonic acid (MOPS) minimal medium (Teknova, Hollister, CA, USA).

The strains were mixed in 1:1 ratio (v/v) prior to inoculation and OD600 measured (the inoculum OD600 was 6.82). The mixed culture was used to inoculate a 3 L glass autoclavable bioreactor (Applikon Biotechnology, Delft, The Netherlands) with 1 L of 1x MOPS minimal medium (Teknova, Hollister, CA, USA) containing 0.5 g/L glucose and 1.5 g/L lactose as the only carbon sources (Traxler et al. 2006; Mostovenko et al. 2011). Bioreactor temperature was maintained at 37°C ($\pm 0.3$°C) via a recirculating water bath (OLS200, Grant Instruments). Culture pH was monitored and logged via a Bio Controller (ADI 1010, Applikon Biotechnology, Delft, The Netherlands) and maintained at pH $7.2\pm 0.05$ by addition of 2 M NaOH. Dissolved oxygen was maintained above 20% saturation by adjusting agitation speed in the range of 270 - 500 rpm (Motor Controller, ADI 110, Applikon Biotechnology, Delft, The Netherlands) with fixed 1 L/min air flow (Traxler et al. 2006).

To monitor cell density and glucose and lactose concentration, 2 mL samples were collected every 30 min before and after diauxie and every 10 min near and during the diauxic shift, as described in Mostovenko et al. (2011). *E. coli* growth was measured by assessing OD600 using a Thermo Spectronic Biomate 3 UV-Visible spectrophotometer (ThermoFisher Scientific, UK) zeroed against an uninoculated growth medium blank. For large values of OD600 ($> 0.4$), we calculated OD600 based on samples that were diluted in media and remeasured. The concentrations of glucose and lactose were assayed using enzymatic kits (CBA086, Sigma-Aldrich and K624, BioVision, respectively). Aliquots of cells were also cultured on MacConkey agar and incubated at 37°C overnight for differentiation and enumeration of lactose and non-lactose fermenting strains.

**Fig. 4:**
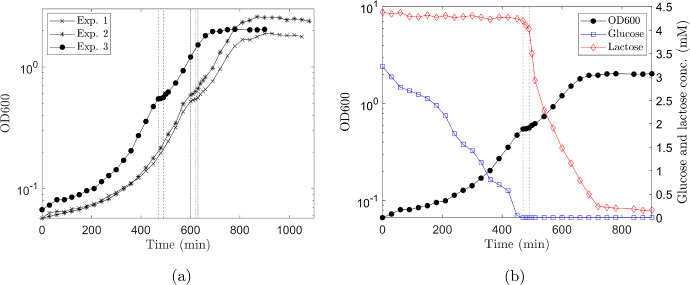
Mixed *E. coli* strains diauxic growth profile on glucose and lactose. **a** Growth curves of three independent biological replicates illustrating the transitions between the initial lag phase, lag phase due to diauxic shift and the stationary phase as all sugars are depleted. **b** Glucose and Lactose concentrations relating to different parts of the growth curve for replicate experiment 3; black line and circles in **a**, shown again here for completeness. Glucose (blue line, squares) is initially depleted by both strains (MV1717, MV1300) before a lag phase induced by the diauxic shift to lactose fermentation. Vertical dashed lines indicate the passage of diauxic shift (Color figure online)

Cell density and glucose and lactose concentration measurements allowed the accurate establishment of the initial lag phase (caused by the switch from growth on rich LB to minimal media) and the onset of diauxic growth, see Fig. 4.

**Fig. 5:**
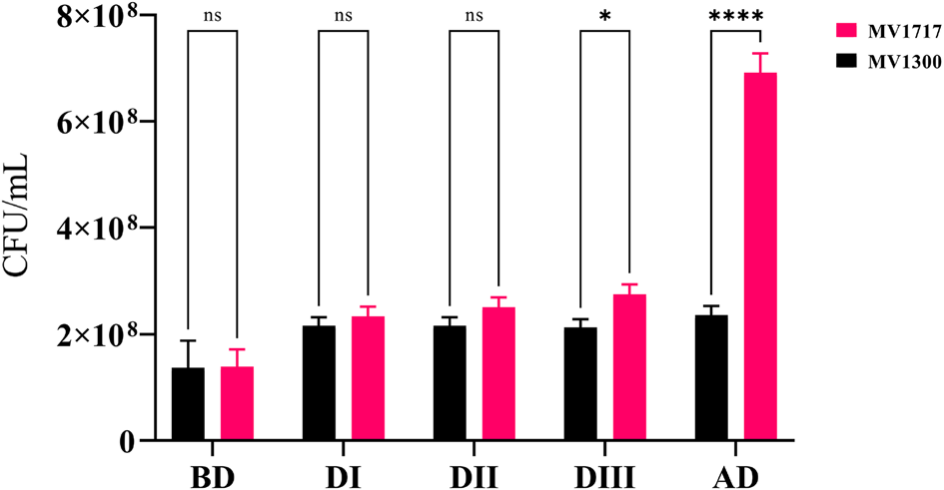
The enumeration of lactose (MV1717) and non-lactose (MV13000) fermenting strains on MacConkey agar. BD = samples in exponential growth before the diauxic shift (420 min); DI–DIII = samples during the diauxic shift (470, 480 and 490 min, respectively); AD = samples after the diauxic shift and during exponential growth (600 min). Bars represent the standard deviation of three replicates (n = 3). The significance of differences was analysed by two-way ANOVA test (****$P<0.0001$; ns, not significant) and performed using GraphPad Prism software version 9.0.2 (Motulsky 2021) (Color figure online)

During the initial lag phase (up to around 200 min) substrate is taken up (the glucose level declines; see blue line and squares in Fig. 4b) but the growth rate is significantly less (the gradient of the growth curve, black line and circles in Fig. 4b, is much less for $t<200$ min than for $t>200$ min). The longer initial lag phase observed in experiments 1 and 2 is likely due to the fact that the two cultures used to inoculate experiments 1 and 2 were slightly older (20 h post inoculation) compared to experiment 3 (18 h post inoculation). Diauxie began when the culture reached OD600 of $\sim 0.5$ or a density of approximately $4 \times 10^8$ cells/mL (Brown 2010) and was indicated by a $20{-}30$ min plateau in the growth curve (Fig. 4). The OD600 of the diauxic shift was comparable in three experiments (OD600 of 0.52, 0.59, 0.55), see Fig. 4a and Mostovenko et al. (2011). The onset of diauxie corresponded to exhaustion of glucose in the growth media. Lactose was depleted at around 250 min after the diauxic shift and growth reached stationary phase when OD600 $\sim 2$. Data for qPCR indicate that MV1717 and MV1300 are present in approximately equal numbers while glucose is available in the media but that only MV1717 continues to grow after the lag phase associated with the switch to fermenting lactose, see Fig. 5. These cells reach stationary phase once the sugar sources have been depleted.

These results clearly demonstrate the lag phases associated with switching from rich to minimal media and glucose/lactose diauxie. Growth is interrupted and then resumed as the cells switch metabolic pathways.

#### Comparison with Experiment

We have two strains of *E. coli*, with concentrations $X_1$ and $X_2$, one of which, $X_2$, cannot grow on lactose. Both strains are initially grown on a rich media (LB broth). The strains are then mixed in a 1 : 1 ratio and transferred to a minimal media containing a mixture of glucose (0.5g/L) and lactose (1.5g/L) as the only carbon source. Measurements of the concentrations of glucose, $S_{\textrm{gl}}$, lactose, $S_{\textrm{la}}$, and total biomass, $X_1+X_2$, are taken at intervals from the point at which the strains are transferred to the minimal media, $t=0$.

**Fig. 6:**
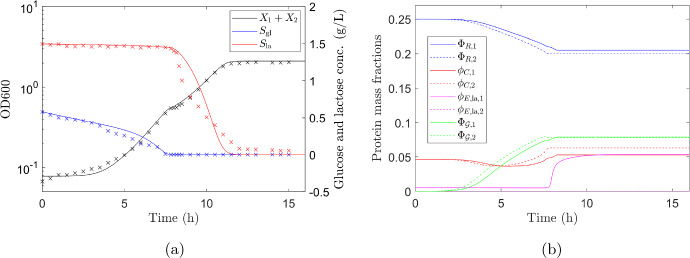
Mixed *E. coli* strains diauxic growth profile on glucose and lactose. **a** Solid lines show numerical predictions of glucose (blue) and lactose (red) concentrations and growth curve (black). Experimental data (Experiment 3 in Fig. 4) are shown as crosses. The growth curve is the sum of biomass of both strains, $X_1+X_2$. The model predicts a sequence of regimes. Initially there is very slow biomass growth even though substrate is being taken up, which is in good agreement with the experimental data. The diauxic shift can clearly be seen in the predicted growth curve at around 8 h, in agreement with experiment. **b** Mass fractions of R-sector proteins (blue), C-sector proteins (red), lactose specific enzyme (magenta) and A-sector proteins (green) for strain 1 (solid lines) and strain 2 (dashed lines). The initial low level of A-sector proteins (${\bar{\Phi }}_\mathcal {G}$) results in slow growth and, as protein production is proportional to growth rate, slow change in protein mass fractions. As the growth rate increases the mass fractions move towards their optimum levels for glucose consumption. Strain 2 stops growing when glucose is depleted and its protein mass fractions stop changing. Strain 1 begins to consume lactose and readjusts its protein mass fractions, most notably the level of lactose specific enzyme, towards levels optimum for lactose consumption. Strain 1 stops growing when lactose is depleted, at around 12 h, and its protein mass fractions stop changing (Color figure online)

Full details of the governing equations and parameters used in the numerical simulations are given in Appendix H. The equations were solved in Matlab (MATLAB 2020) using the solver ode15s. Results showing the predicted concentration of sugars and total biomass over time are shown as the solid lines in Fig. 6a with the experimental data (shown as crosses) plotted for comparison. The mass fractions of the proteome sectors are plotted in Fig. 6b for both strains.

The model predicts very slow initial biomass growth even though substrate is being taken up, which is in good agreement with the experimental data. The initial slow growth is due to the protein mass fractions being at non-optimum levels for growth on glucose in a minimal media. The strains, having previously been growing in a rich media, have a low level of anabolic A-sector proteins, hence ${\bar{\Phi }}_\mathcal {G}$ is small (see Fig. 6b), resulting in slow growth. As the growth rate increases, the mass fractions move towards their optimum levels for glucose consumption. On depletion of glucose, strain 2 stops growing and its protein mass fractions stop changing. The protein mass fractions of strain 1, notably that of the lactose specific enzyme, are at non-optimal levels for lactose consumption and its growth slows, the lag phase. We measure the lag duration as the period when growth rate has dropped below $50\%$ of the maximum on glucose. The model predicts the lag-phase to occur between 450 and 475 min in good agreement with the experimentally observed lag phase between 470 and 490 min. (The accuracy of determining the lag-phase duration from the data is obviously constrained by the frequency of measurements, in this case every 30 min pre and post diauxic shift and every 10 min during the shift).

As strain 1 begins to consume lactose its protein mass fractions adjust to optimise lactose consumption, most notably the mass fraction of the lactose specific enzyme. Strain 1 stops growing when lactose is depleted, at around 12 h, and its protein mass fractions stop changing. There are differences between model predictions of lactose concentration (zero at 12 h) and experimental data (lactose not fully depleted after 12 h). As a microorganism enters the stationary phase different proteins, required for survival in nutrient deprived conditions, must be expressed (Jaishankar and Srivastava 2017). We do not consider this in our model, as we are primarily focused on describing the lag phases, and this may explain the observed discrepancies.

It can be seen from Fig. 6a that our description captures all principal features of the non-trivial growth curve of *E. coli* glucose–lactose diauxie. The lag-phase and diauxic shift are reproduced accurately using our rather fundamental model with minimal fitting and without the need for introducing an artificial lag parameter. All phases of growth, including the initial lag and diauxic shift, are determined from the structure of the microorganism’s proteome.

We now present the results of a sensitivity analysis looking at how changes to the fitted parameters affect the model predictions.

### Sensitivity Analysis

The parameters $Y^*_{\textrm{gl}}$, $Y^*_{\textrm{la}}$, $\Phi _{\mathcal {G},0}$, $K_L$ and $\epsilon$ were determined to give a best fit to both the Erickson et al. (2017) data and our experimental data. For consistency, all parameters (fitted and those taken from the literature) have the same values for both simulations with the exception of the log-phase yields $Y^*_{\textrm{gl}}$ and $Y^*_{\textrm{la}}$. This is because the yield depends on the ratio of OD600 to g/L of biomass which will differ between the Erickson et al. (2017) experiment and our own experiment. We do, however, keep the ratio $Y^*_{\textrm{gl}}:Y^*_{\textrm{la}}$ the same for both simulations.

**Fig. 7:**
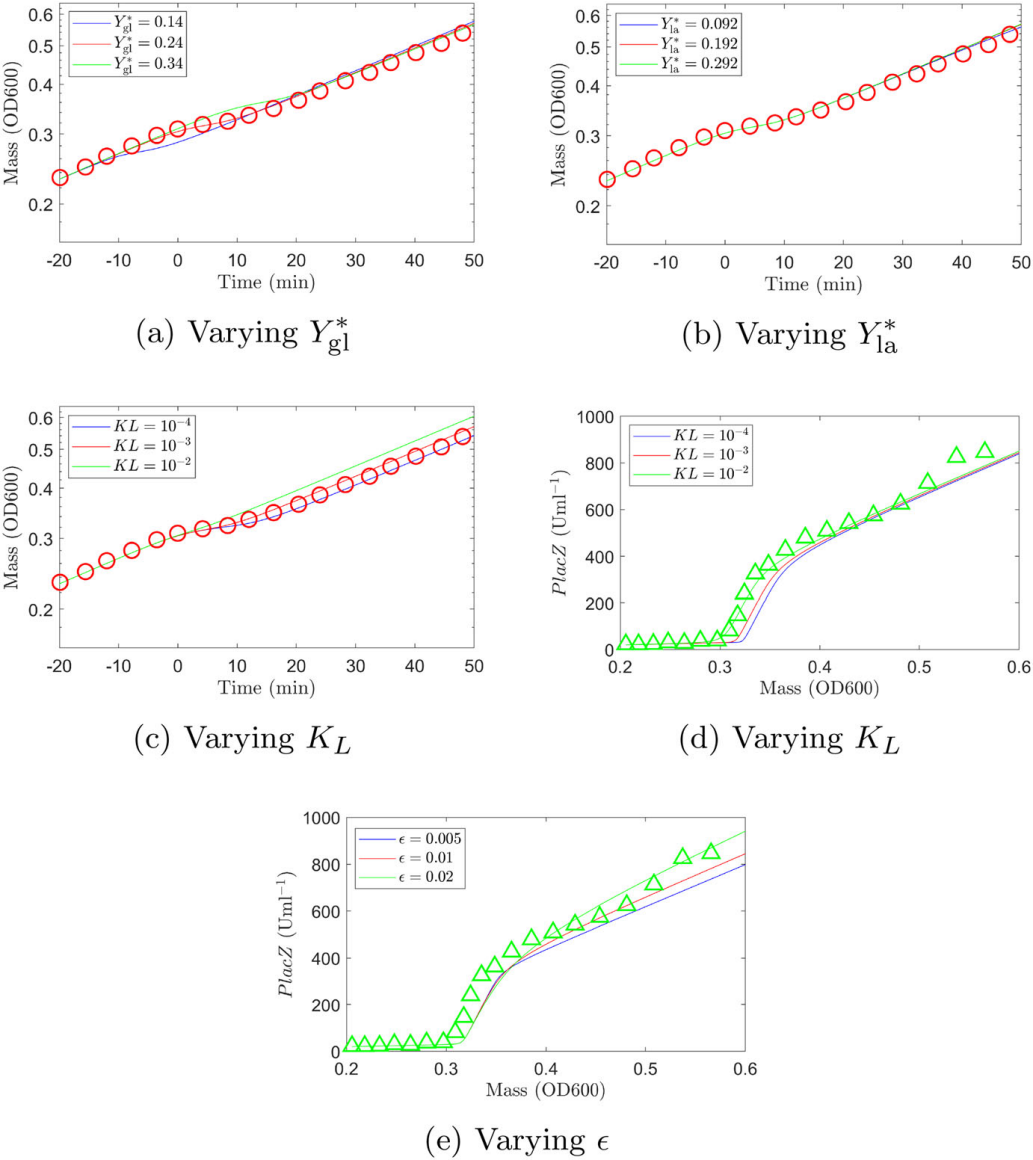
Comparison of numerical solutions when values of fitted parameters are varied (experimental data shown as red circles (biomass) and green triangles (PlacZ concentration)). **a** The fitted value, $Y^*_{\textrm{gl}}=0.24$, is shown in red. Reducing $Y^*_{\textrm{gl}}$ ($Y^*_{\textrm{gl}}=0.14$ shown in blue) decreases the biomass growth on glucose and brings forward the time of diauxic shift to $t\approx -10$ min. Conversely, increasing $Y^*_{\textrm{gl}}$ ($Y^*_{\textrm{gl}}=0.34$ shown in green) increases the biomass growth on glucose and the diauxic shift occurs later at around $t=10$ min. **b** The fitted value, $Y^*_{\textrm{la}}=0.192$, is shown in red. Reducing $Y^*_{\textrm{la}}$ ($Y^*_{\textrm{la}}=0.092$ shown in blue) or increasing $Y^*_{\textrm{la}}$ ($Y^*_{\textrm{la}}=0.292$ shown in green) has little effect on the predicted growth curve. **c**, **d** The fitted value, $K_L=10^{-3}$, is shown in red. Reducing $K_L$ ($K_L=10^{-4}$ shown in blue) means that the glucose level must reach a lower value before the lactose specific enzyme is expressed. This increases the length of the diauxic lag. Conversely, increasing $K_L$ ($K_L=10^{-2}$ shown in green) means that the lactose specific enzyme is expressed at higher glucose levels which shortens the diauxic lag. The fitted value was chosen to give a best fit to both the growth curve and enzyme expression level curve. **e** The fitted value, $\epsilon =0.01$, is shown in red. Reducing $\epsilon$ ($\epsilon =0.005$ shown in blue) decreases the production rate of lactose specific enzyme more quickly. Conversely, increasing $\epsilon$ ($\epsilon =0.02$ shown in green) means that the production rate of lactose specific enzyme is higher for longer (Color figure online)

**Fig. 8:**
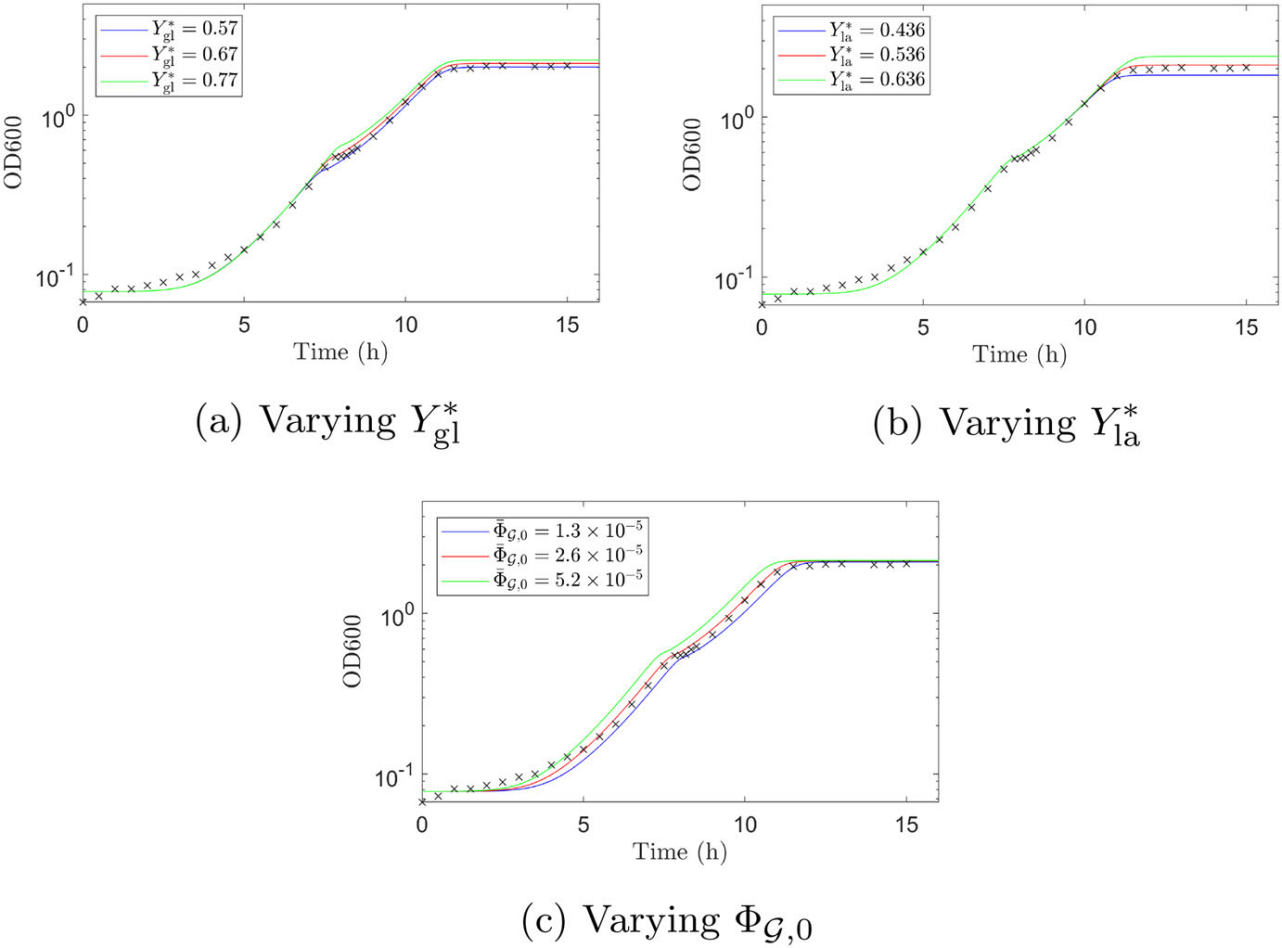
Comparison of numerical solutions when values of fitted parameters are varied (experimental data shown as black crosses). **a** The fitted value, $Y^*_{\textrm{gl}}=0.67$, is shown in red. Reducing the log-phase yield ($Y^*_{\textrm{gl}}=0.57$ shown in blue) decreases the biomass concentration. Conversely, increasing the log-phase yield ($Y^*_{\textrm{gl}}=0.77$ shown in green) increases the biomass concentration. **b** The fitted value, $Y^*_{\textrm{la}}=0.536$, is shown in red. Reducing the log-phase yield ($Y^*_{\textrm{la}}=0.436$ shown in blue) decreases the biomass concentration post diauxic shift. Conversely, increasing the log-phase yield ($Y^*_{\textrm{la}}=0.0.436$ shown in green) increases the biomass concentration post diauxic shift. **c** The fitted value, $\Phi _{\mathcal {G},0}=2.6\times 10^{-5}$, is shown in red. Reducing $\Phi _{\mathcal {G},0}$ ($\Phi _{\mathcal {G},0}=1.3\times 10^{-5}$ shown in blue) reduces the initial growth rate, increasing the time taken for the protein mass fractions to reach their optimum levels thus increasing the length of initial lag phase. The final biomass yield is also reduced. Conversely, increasing $\Phi _{\mathcal {G},0}$ ($\Phi _{\mathcal {G},0}=5.2\times 10^{-5}$ shown in green) increases the initial growth rate, shortens the initial lag phase and increases final biomass concentration. The qualitative behaviour of the growth curve is similar for all cases shown (Color figure online)

The best fit values are given in Table 5 of Appendix G. The log-phase yields in the table are those fitted to the Erickson et al. (2017) data; fitting to our data gives $Y^*_{\textrm{gl}}=0.67$ and $Y^*_{\textrm{la}}=0.536$.

Figures 7 and 8 show results from simulation 1 and 2 respectively for different values of the fitted parameters (experimental data is also shown for comparison). The parameters $K_L$ and $\epsilon$ were fitted to simulation 1 as in this case we have data for enzyme expression levels as well as biomass. The parameter ${\bar{\Phi }}_{\mathcal {G},0}$ was fitted to simulation 2 as it has negligible effect on the results of simulation 1 as there is no initial lag phase. The log-phase yields were fit to both simulations seperately, as discussed above.

In Figs. 7 and 8 the predicted curve using the best fit parameters is shown in red. In each subfigure one parameter is varied while all other parameters are fixed (to the values given in Tables 4 and 5 in Appendix G). As expected, changing the yields on glucose and lactose alters the final biomass concentration; a lower yield value giving lower final biomass concentration and vice versa (see Figs. 7a, b, and 8a, b). This is more evident for simulation 2 (Fig. 8a, b) where the simulation is run until lactose is depleted.

The constant $K_L$ determines when the lactose specific enzyme starts to be expressed via the function

$$\begin{aligned} \eta _{\textrm{la}}&= \frac{K_L^2+\xi S_{\textrm{gl}}^2}{K_L^2+S_{\textrm{gl}}^2}, \end{aligned}$$

and $\epsilon$ affects the rate of production of lactose specific enzyme via the function

$$\begin{aligned} \zeta _{\textrm{la}}&= \frac{1}{2}\left( 1-\tanh \left( \frac{1}{\epsilon }\left( \frac{{\bar{\phi }}_{E,\textrm{la}}}{\phi _{C}}-\frac{1}{2}\right) \right) \right) . \end{aligned}$$

For values of $S_{\textrm{gl}}\gg K_L$ we have $\eta _{\textrm{la}}\approx \xi \ll 1$ and the lactose specific enzyme is expressed only at a very low level. This expression level increases only when glucose levels drop so that $S_{\textrm{gl}}\approx K_L$. Reducing $K_L$ increases the length of the diauxic lag phase as glucose levels must reach a lower value before lactose begins to be consumed (Fig. 7c, d). If on the other hand $K_L$ is increased the lag phase will shorten, with the extreme case $K_L\rightarrow \infty$ ($\eta _{\textrm{la}}=1$) removing the diauxic lag phase completely (glucose and lactose are consumed simultaneously). The function $\zeta _{\textrm{la}}$ is essentially a smoothed-out step function ($\zeta _{\textrm{la}} = 1$ for ${\bar{\phi }}_{E,\textrm{la}}< \phi _C/2$ and $\zeta _{\textrm{la}} = 0$ for ${\bar{\phi }}_{E,\textrm{la}}\ge \phi _C/2$) with the parameter $\epsilon$ defining the sharpness of the step (as $\epsilon \rightarrow 0$ we tend towards a step function, for larger $\epsilon$ the change from 1 to 0 as ${\bar{\phi }}_{E,\textrm{la}}$ increases is more gradual). For small $\epsilon$ (a steep changing $\zeta _{\textrm{la}}$) the production of lactose specific enzyme is reduced from the maximum level $\Phi _{\textrm{max}}$ quite sharply when ${\bar{\phi }}_{E,\textrm{la}}\approx \phi _C/2$. As $\epsilon$ increases the production rate drops more slowly as ${\bar{\phi }}_{E,\textrm{la}}$ increases past ${\bar{\phi }}_{E,\textrm{la}}\approx \phi _C/2$. This means that for larger $\epsilon$ we have a higher production rate of enzyme for longer, as can be seen in Fig. 7e where the expression level of lactose specific enzyme increases at a higher rate for larger values of $\epsilon$.

Changing the minimum mass fraction of the key anabolic protein, $\Phi _{\mathcal {G},0}$, alters the length of the initial lag phase and the final combined biomass concentration (Fig. 8c). The lower the value of $\Phi _{\mathcal {G},0}$ the slower the initial growth rate, increasing the time taken for the protein mass fractions to reach their optimum levels thus increasing the length of initial lag phase. The final biomass yield is also less for smaller $\Phi _{\mathcal {G},0}$.

### Applying the Model to Investigate Different Growth Strategies

Diauxic growth is usually regarded as a process by which a microorganism maximises growth, however, during the diauxic lag phase there is a significant loss of growth. There is a trade-off between consuming the preferred sugar efficiently, maximising the microorganism’s long-term growth, and lost growth during the switch as the microorganism adjusts to using the secondary sugar. Are there conditions under which diauxic behaviour is an advantage and others which favour simultaneous consumption of resources?

When resources are scarce, a strain that can outgrow its competitors will have an advantage (Giordano et al. 2016). In the following we compare two strains with the same initial biomass, hence we define the ‘better’ growth strategy as belonging to the strain with a higher final biomass concentration.

To examine whether diauxie is beneficial for growth we use our parameterised model to simulate the growth of two different strains of *E. coli*. We let $X_{\textrm{D}}$ denote a diauxic strain (the same as *X* in simulation 1) and introduce a theoretical strain, $X_{\textrm{ND}}$, which does not exhibit diauxie. This non-diauxic strain consumes glucose and lactose simultaneously, so that $\eta _{\textrm{la,ND}}=1$ (this is the limit $K_L\rightarrow \infty$ noted in Sect. 3.3). All other growth parameters are assumed to be the same as for $X_{\textrm{D}}$ given in Tables 4 and 5. Initial conditions for both strains are identical and equal to those used in simulation 1, described in Appendix G, with the exception of those for the lactose specific enzyme. For $X_{\textrm{D}}$ we have ${\bar{\phi }}_{E,\textrm{la,D}}=\xi \Phi _{\textrm{max}}$ (the pre-expression level) and for $X_{\textrm{ND}}$ we have ${\bar{\phi }}_{E,\textrm{la,ND}}=\phi _C$ as the enzyme will always be expressed for this strain (lactose consumption is not inhibited for the non-diauxic strain).

**Fig. 9:**
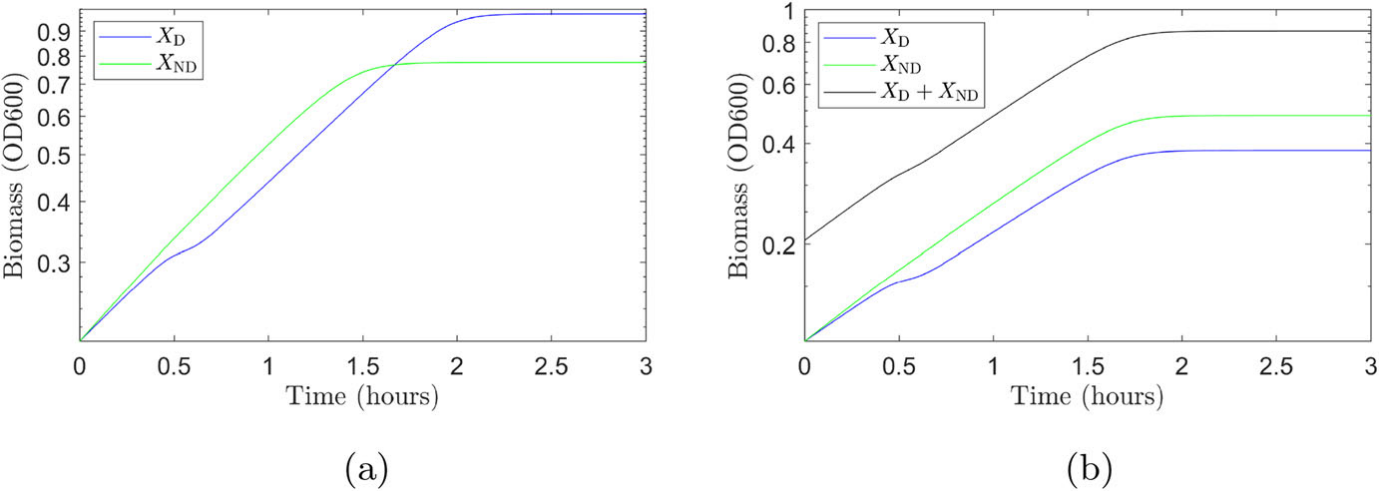
Growth curves for *E. coli* strains growing on a 0.3 g/L glucose/2.0 g/L lactose mixture. **a** Growth curves from two simulations with only one strain present (no competition): diauxic strain $X_{\textrm{D}}$ (blue); and non-diauxic strain $X_{\textrm{ND}}$ (green). The strain exhibiting diauxie, $X_{\textrm{D}}$ has a higher final biomass as it is able to consume glucose efficiently in the presence of lactose. **b** Growth curves from one simulation where two strains $X_{\textrm{D}}$ (blue) and $X_{\textrm{ND}}$ (green) are competing for resources. The non-diauxic strain, $X_{\textrm{ND}}$, out-competes the diauxic strain, $X_{\textrm{D}}$. Initially $X_{\textrm{ND}}$ has a higher growth rate than $X_{\textrm{D}}$ as it consumes both glucose and lactose simultaneously. In addition, it is able to consume lactose efficiently immediately on exhaustion of glucose. These two factors give it a competitive advantage over the diauxic strain (Color figure online)

We first use our parameterised model to predict growth curves when only $X_{\textrm{D}}$ is present and when only $X_{\textrm{ND}}$ is present. We then simulate the growth for the two strains in competition (for this simulation we assume the initial biomass to be split equally between the two strains). The results are shown in Fig. 9. When only one strain is present (Fig. 9a) the final biomass concentration is higher for $X_{\textrm{D}}$ (blue) than $X_{\textrm{ND}}$ (green): in this case it is beneficial to grow diauxically. The diauxic strain blocks the uptake of lactose when glucose is present, using all of the cells’ resources to metabolise glucose. The non-diauxic cells must share their resources to break down glucose and lactose simultaneously, reducing the efficiency and lowering the final biomass yield. If however, we look at the case where the diauxic and non-diauxic strains are competing for resources, growth curves are shown in Fig. 9b, we find that $X_{\textrm{ND}}$ (green) outgrows $X_{\textrm{D}}$ (blue). The total biomass, $X_{\textrm{D}}+X_{\textrm{ND}}$ (black curve), is lower than in the case of growth only on $X_{\textrm{D}}$ (blue curve in Fig. 9a) but higher than that on only $X_{\textrm{ND}}$ (green curve in Fig. 9a). When both sugars are present, the growth rate of the non-diauxic strain (consuming two sugars simultaneously) is higher that that of the diauxic strain giving it a competitive advantage. In addition the non-diauxic strain has no pause in growth on depletion of glucose, no diauxic shift, giving it a further advantage over the diauxic strain.

**Fig. 10:**
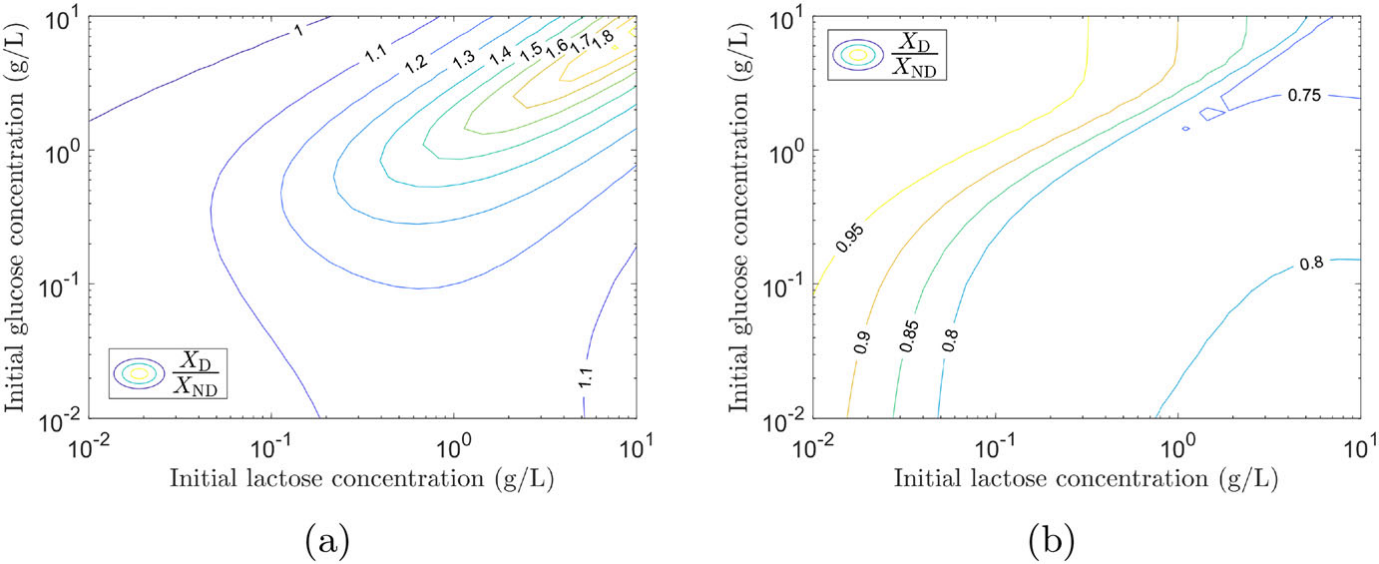
Comparison of two strains of *E. coli*, $X_{\textrm{D}}$ which exhibits diauxie and $X_{\textrm{ND}}$ which has no diauxic shift, growing in a glucose/lactose mixture. **a** Non-competitive simulations results. Final biomass ratio $X_{\textrm{D}}/X_{\textrm{ND}}$ from simulations with varying different initial concentrations of glucose and lactose. The diauxic strain has a higher final biomass ($X_{\textrm{D}}/X_{\textrm{ND}}>1$) for the majority of initial concentrations of glucose and lactose. The non-diauxic strain has a higher final biomass ($X_{\textrm{D}}/X_{\textrm{ND}}<1$) only when the initial glucose concentration is much larger than the initial lactose concentration. **b** Competitive simulations results. Final biomass ratio $X_{\textrm{D}}/X_{\textrm{ND}}$ from simulations with varying different initial concentrations of glucose and lactose. The non-diauxic strain always has a higher final biomass ($X_{\textrm{D}}/X_{\textrm{ND}}<1$) (Color figure online)

We find that when a single strain of *E. coli* is growing on a mixture of glucose (0.3 g/L) and lactose (2.0 g/L), it is better to consume the two sugars sequentially, however, when strains are competing for these resources it is not necessarily beneficial for a strain to grow diauxically. To investigate whether this remains the case for different glucose–lactose mixtures, the simulations were repeated for a range of different initial concentrations of glucose and lactose. Each simulation was run until all sugars were depleted and the final biomass concentration for each strain was obtained. The ratio of the final biomass of the two strains $X_{\textrm{D}}/X_{\textrm{ND}}$ was calculated. The results for the non-competitive case are shown in Fig. 10a. The diauxic strain performs better, $X_{\textrm{D}}/X_{\textrm{ND}}>1$, for the majority of initial concentrations of glucose and lactose. The non-diauxic strain has a higher biomass, $X_{\textrm{D}}/X_{\textrm{ND}}<1$, only when the initial concentration of glucose is much higher than the initial concentration of lactose. Figure 10b shows the results for the competitive case. The non-diauxic strain always has a higher final biomass, $X_{\textrm{D}}/X_{\textrm{ND}}<1$, when the strains are competing for the same resources.

The results in Fig. 9 show that on a mixture of 0.3 g/L glucose 2.0 g/L lactose, the diauxic strain has a lower growth rate but a higher biomass yield than the non-diauxic strain. By running the simulations over a range of initial concentrations of glucose and lactose, results shown in Fig. 10, we find that, except when the initial concentration of glucose is much higher than the initial concentration of lactose, we have the same situation: the diauxic strain has a lower growth rate but a higher biomass yield than the non-diauxic strain. We infer that diauxic growth is the optimal growth strategy in a non-competitive environment, where the maximisation of growth yield is an advantage (Giordano et al. 2016), whereas in a competitive environment, where maximising growth rate is an advantage (Ibarra et al. 2002), diauxic growth is not the optimum growth strategy.

Studies have shown that strains of bacteria (Spencer et al. 2007) and yeast (Wang et al. 2015) can evolve to have differing lengths of diauxic shift. When a microorganism is subject to frequent changes in environment the diauxic lag will evolve to be short, whereas in a stable environment the lag phase will be longer (Chu and Barnes 2016).

For constant sugar concentrations we have a stable growth environment. When sugars are consumed the growth environment changes on depletion of glucose/lactose so the higher the initial sugar concentrations the longer the time before conditions change. Therefore, the environment can be considered stable for a longer period as the initial concentrations of the two sugars increases. The results in Fig. 10a show that the advantage of growing diauxically increases ($X_{\textrm{D}}/X_{\textrm{ND}}$ increases) as initial sugar concentrations increase. Our simulation results agree qualitatively with experimental evidence that bacteria (Spencer et al. 2007) and yeast strains (Wang et al. 2015) with a long diauxic lag perform better in a stable environment and those with a short (or no) diauxic lag perform better in a changing environment.

## Discussion

In this paper, we have formulated a coarse-grained mechanistic model describing the time evolution of biomass growth, substrate concentration and gene expression during carbon upshifts and downshifts. The model extends recent descriptions, incorporating proteome partitioning, flux-controlled regulation and optimal allocation of protein synthesis. Carbon influx is balanced with amino acid and protein synthesis fluxes via adjustments to the amino acid synthesis rate and average translation rate, the rates being determined by the size of pools of central precursors (including ketoacids and amino acids). Here, we recognise that the central precursors are limited by the innate capacity of a cell; the model includes a mechanistic functional response to limit the size of the precursor pools, ruling out physically unrealizable behaviour observed in results from earlier models.

Phases of microorganism growth emerge from the dynamics, rather than being switched on/off at a particular time. The selective use of substrates, regulated by mechanisms such as CCR, is achieved by completely different methods in different microorganisms (Görke and Stülke 2008). Accordingly, the exact mechanism underlying the inhibition of substrate uptake is not made explicit in our model, making it flexible and applicable to processes other than *E. coli* glucose–lactose diauxie, which we have focussed on. The switch to consuming the secondary substrate, controlled through functions $\eta _j$, occurs when the concentration of the preferred substrate drops below a set value, $K_L$. In this way we avoid having to artificially switch on the inferior carbon uptake system at a predetermined time as in other models (Erickson et al. 2017; New et al. 2014).

Furthermore, the regulation functions allocating protein synthesis are derived directly, associated with a mathematical optimisation of the growth rate. Resource allocation in steady state conditions can be determined from fundamental growth laws relating protein levels to growth rate (You et al. 2013; Scott et al. 2010; Erickson et al. 2017; Hui et al. 2015). In dynamic conditions, however, it remains unclear how protein synthesis is regulated. Erickson et al. (2017) construct regulation functions based on the steady state growth laws but these suffer from being undefined or negative during growth transitions. Therefore, we formulated an alternative description of protein allocation, that is valid during growth transitions, where the regulation functions are derived directly via mathematical optimization of the growth rate.

Employing our modelling approach, we found that phases of bacterial growth, including the lag phase and diauxic shift, emerged from the structure of the bacterial proteome. In particular, the deterministic model predicted the diauxic growth of *E. coli* on glucose and lactose, comparing favourably with the model of Erickson et al. (2017) and the related experimental data. Furthermore, unlike earlier models (Pavlov and Ehrenberg 2013; Erickson et al. 2017; Salvy and Hatzimanikatis 2021), our model was able to simulate a more complex system with two successive lag phases; the first lag due to the switch between growth on a rich and a minimal media, the second a diauxic lag. The lag-phase and diauxic shift were reproduced accurately using our rather fundamental model with minimal fitting. The primary focus of the current study was to describe lag and log-phase growth. Therefore, the transition to stationary phase is less well captured (as demonstrated by inconsistencies between predicted and measured lactose concentration post 12 h). This could be addressed by taking account of the expression of proteins required for survival in nutrient deprived conditions (Jaishankar and Srivastava 2017).

Earlier dynamic resource allocation models have focused on predictions of growth rate/biomass and protein levels (Pavlov and Ehrenberg 2013; Erickson et al. 2017). However, these models are unable to capture the non-simple relationship between substrate uptake rate and growth rate observed experimentally during the lag phase. Substrate concentrations have been predicted in the rather large and complex modelling approach of Salvy and Hatzimanikatis (2021). Complex models typically have many undetermined/unmeasurable parameters. We found that our much simpler coarse-grained model was sufficient to describe the time evolution of substrate concentrations in addition to biomass and protein levels, accurately replicating the observed relationship between substrate uptake and biomass growth during lag phase.

When a microorganism switches between carbon sources there is a trade off between optimising growth on the preferred substrate and being able to switch quickly when the primary source is depleted (Chu and Barnes 2016; Basan et al. 2020). We have shown that the lag phase observed when * E coli.* switches from a rich to a minimal media can be explained by a low level of a key anabolic protein causing a bottleneck in the metabolic flux pathway. This agrees with the conclusions of Wu et al. (2023) and Basan et al. (2020) that lag phases are caused by metabolic bottlenecks.

Our investigation into the merits of different bacterial growth strategies finds that in a non-competitive environment, where the maximisation of growth yield is an advantage (Giordano et al. 2016), growing diauxically is the optimum strategy. Conversely, in a competitive environment, where maximising growth rate is an advantage (Ibarra et al. 2002), diauxic growth is not the best strategy. This behaviour is in agreement with results of Chu and Barnes (2016) that premature activation of the secondary metabolism shortens the lag but causes costs to the cell thus reducing the growth rate on the preferred substrate.

Recent work has made clear that microorganisms living in changing environments do not always favour perfect catabolite repression (Wang et al. 2015; New et al. 2014; Siegal 2015). New et al. (2014) found that although stringent catabolite repression seems favourable in relatively stable environments, less stringent regulation can increase fitness in variable conditions. To explore competition in a changing environment we ran simulations starting with a limited amount of sugar. The growth environment changes on depletion of glucose/lactose so the higher the initial sugar concentrations the longer the time before conditions change. The environment is therefore stable for a longer period as the initial concentrations of the two sugars, particularly glucose, increases. Our results show that, in a non-competitive environment the advantage of growing diauxically increases the more stable the environment becomes. Our results compare favourably with the results of New et al. (2014) and other experimental evidence that bacteria (Spencer et al. 2007) and yeast strains (Wang et al. 2015) with a long diauxic lag dominate in a stable environment and those with a short (or no) diauxic lag dominate in a changing environment.

This study adds to the rich body of work showing how microorganisms react to changing environments (Salvy and Hatzimanikatis 2021; Erickson et al. 2017; New et al. 2014; Mori et al. 2019; Wang et al. 2019; Wu et al. 2023). The range of applications of our modelling approach is large: the description can be easily adapted to model multiple different microorganisms, investigate competition between different species or strains and explore other growth strategies. The model can be adapted to predict the growth of many bacteria and yeasts that exhibit diauxie. More generally, the model provides a means to investigate and describe lag phase, the mechanisms for which, despite many years of research, are only just being revealed.

## Data Availability

All data generated or analysed during this study are included in this published article.

## Appendix A: The Values of the Mass Fractions During the Log Phase of Growth

It has been shown experimentally that during the log phase of growth of bacterial cells, the rate of cell proliferation (the growth rate) and the expression levels of key proteins are linearly correlated (You et al. 2013; Scott et al. 2010; Erickson et al. 2017). In these experiments *E. coli* cells are grown on a range of different nutrients. Once the cells reach the log phase of growth measurements of protein expression levels and the growth rate are taken. In the following a star denotes the value of a variable during the log phase of growth.

### R-Sector Proteins

Experimentally the RNA/protein ratio, *r*, which is a well established proxy for the ribosomal mass fraction (Erickson et al. 2017), is measured rather than $\Phi _R$ itself. For cells growing exponentially under nutrient (e.g. carbon or nitrogen) limitation, *r* is linearly correlated with the growth rate (Scott et al. 2010) and we have the following bacterial growth law

$$\begin{aligned} \Phi _R^*=&\Phi _{R,0}+\frac{\lambda ^*}{\nu _R},{} & {} \text{ or }&\phi _R^*=&\frac{\lambda ^*}{\nu _R}, \end{aligned}$$

where $\lambda ^*$ is the growth rate of *E. coli* cells during log phase, $\Phi _{R,0}$ is the growth independent minimum level of $\Phi _R$ and $\nu _R$ is a constant (Erickson et al. 2017). Values of these parameters taken from the literature are shown in Table 3 (the relation $\Phi _R=0.46\,r$ was used to fit the data in Erickson et al. (2017)).

### C-Sector Proteins

The value of $\phi _C^*$ is determined by assuming proportionality to a reporter enzyme (P*LacZ* in You et al. (2013) and Erickson et al. (2017)). Experimentally it has been shown (You et al. 2013; Scott et al. 2010; Erickson et al. 2017) that when carbon is limiting growth we have

$$\begin{aligned} L^*=L_{\textrm{max}}\left( 1-\frac{\lambda ^*}{\lambda _C}\right) , \end{aligned}$$

where $L_{\textrm{max}}$, the maximum level of the reporter enzyme, *L*, and the constant $\lambda _C$ are determined by fitting to experimental data. Values of these parameters taken from the literature are shown in Table 3.

The growth dependent part of the catabolic protein sector is assumed to be regulated as a whole (You et al. 2013; Hui et al. 2015). If a catabolic enzyme is not being repressed its expression level will therefore be proportional to the expression level of the total catabolic sector. The mass fraction of such a catabolic enzyme, $\Phi _{E,j}$, therefore satisfies

A1 $$\begin{aligned} \Phi _{E,j}-\Phi _{E,j,0}=\beta _{E,j}\left( \Phi _C-\Phi _{C,0}\right) =\beta _{E,j}\phi _C, \end{aligned}$$

where $\Phi _{E,j,0}$ is the growth independent minimum level of the enzyme and $\beta _{E,j}$ is a constant ($\beta _{E,j}=(\Phi _{E,j,\textrm{max}}-\Phi _{E,j,0})/\Phi _{\textrm{max}}$). For the reporter enzyme, *L*, we therefore have $L-L_0=\beta _L\phi _C$, where $L_0$ is the growth independent minimum level of reporter enzyme (the total protein level is independent of growth rate (You et al. 2013) so *L* is directly proportional to the mass fraction of reporter enzyme). When the catabolic sector is at its maximum this gives $L_{\textrm{max}}-L_0=\beta _L\Phi _{\textrm{max}}$ and it follows that

$$\begin{aligned} \phi _C^*=\Phi _{\textrm{max}}\frac{L^*-L_0}{L_{\textrm{max}}-L_0}=\Phi _{\textrm{max}}\left( \frac{L_{\textrm{max}}}{L_{\textrm{max}}-L_0}\left( 1-\frac{\lambda ^*}{\lambda _C}\right) -\frac{L_0}{L_{\textrm{max}}-L_0}\right) . \end{aligned}$$

Experimental results show that $L_{\textrm{max}}\gg L_0$ (You et al. 2013; Erickson et al. 2017) so the above expression can be simplified by neglecting small terms to give

$$\begin{aligned} \phi _C^*=\Phi _{\textrm{max}}\left( 1-\frac{\lambda ^*}{\lambda _C}\right) . \end{aligned}$$

Rescaling the mass fraction so that ${\bar{\Phi }}_{E,j}=\Phi _{E,j}/\beta _{E,j}$ we can remove $\beta _{E,j}$ from equation A1. Further assuming that $\Phi _{E,j,\textrm{max}}\gg \Phi _{E,j,0}$ (as supported by experimental evidence (You et al. 2013)), equation A1 reduces to to ${\bar{\Phi }}_{E,j}=\phi _C$, which holds for all non-repressed catabolic enzymes. It follows that

$$\begin{aligned} {\bar{\Phi }}_{E,j}^*=\Phi _{\textrm{max}}\left( 1-\frac{\lambda _j^*}{\lambda _C}\right) , \end{aligned}$$

for all catabolic enzymes.

### A-Sector Proteins

The A-sector is assumed to be regulated as a whole (You et al. 2013; Hui et al. 2015) so for an anabolic protein, $\mathcal {G}$, with mass fraction $\Phi _\mathcal {G}$, we have

A2 $$\begin{aligned} \Phi _\mathcal {G}-\Phi _{\mathcal {G},0}=\beta _{\mathcal {G}}\left( \Phi _A-\Phi _{A,0}\right) , \end{aligned}$$

where $\Phi _{\mathcal {G},0}$ is the growth independent minimum level of protein $\mathcal {G}$ and

$$\begin{aligned} \beta _{\mathcal {G}}=\frac{\Phi _{\mathcal {G},\textrm{max}}-\Phi _{\mathcal {G},0}}{\Phi _{\textrm{max}}}. \end{aligned}$$

Rescaling the mass fraction so that ${\bar{\Phi }}_{\mathcal {G}}={\Phi _{\mathcal {G}}}/{\beta _{\mathcal {G}}}$, we can remove $\beta _G$ from Eq. (A2) obtaining

A3 $$\begin{aligned} {\bar{\Phi }}_{\mathcal {G}}={\bar{\Phi }}_{\mathcal {G},0}+\left( \Phi _A-\Phi _{A,0}\right) ={\bar{\Phi }}_{\mathcal {G},0}+\phi _A. \end{aligned}$$

The value of $\phi _A$ can be determined from $\phi _R$ and $\phi _C$: from Eq. (2) we have

$$\begin{aligned} \phi _A=\Phi _{\textrm{max}}-(1+\varepsilon )\phi _R-\phi _C, \end{aligned}$$

which yields

$$\begin{aligned} \phi _A^*=\frac{\lambda ^*}{\nu _A}, \end{aligned}$$

with

$$\begin{aligned} \nu _A=\left( \frac{\Phi _{\textrm{max}}}{\lambda _C}-\frac{(1+\varepsilon )}{\nu _R}\right) ^{-1}. \end{aligned}$$

**Table 3:** Parameter values taken from the literature that are used in the equations defining the value of mass fractions during the log phase

RNA/protein ratio (C-limitation)		Ribosomal mass fraction (C-limitation)	
$r^*=r_{0}+\frac{\lambda ^*}{\nu _R}^{\textrm{a}}$		$\Phi _R^*=\Phi _{R,0}+\frac{\lambda ^*}{\nu _R}^{\textrm{a}}$	
$r_0$	$\nu _R$ (h$^{-1}$)	$\Phi _{R,0}$	$\nu _R$ (h$^{-1}$)
$0.076\pm 0.005^{\textrm{b}}$	$5.3^{\textrm{b}}$	$0.049\pm 0.02^{\textrm{c}}$	$11.02\pm 0.44^{\textrm{c}}$

P*LacZ* expression (C-limitation)			
$$L^*=L_{\textrm{max}}\left( 1-\frac{\lambda ^*}{\lambda _C}\right) $$			
$L_{\textrm{max}}$ (MU$\times 10^3$)	$\lambda _C$ (h$^{-1}$)		
$32\pm 1^{\textrm{b}}$	$1.2\pm 0.0^{\textrm{b}}$		
$21.8\pm 0.5^{\textrm{c}}$	$1.17\pm 0.05^{\textrm{c}}$		

These equations are described fully in the text

$^{\textrm{a}}$ The two expressions for the ribosomal bacterial growth law given in You et al. (2013) and Erickson et al. (2017) respectively are interchangeable. In Erickson et al. (2017) it is presented in terms of the ribosomal mass fraction, although the RNA/protein ratio is measured, and the relation $\Phi _R=0.46r$ is used to fit the model to data. Further details are given in Sects. A.1 and A.2

$^{\textrm{b}}$ Data from You et al. (2013).

$^{\textrm{c}}$Data from Erickson et al. (2017).

## Appendix B: Substrate uptake—Michaelis–Menten Kinetics

Following Michaelis–Menten kinetics (Murray 2013), substrates with concentrations $S_j$ are broken down by a key catabolic enzyme, $E_j$, in the following way

where $P_j$ represents the product of the reaction. The reactions to form the enzyme-substrate complexes $SE_j$ are assumed to be reversible with the rate of the forward reaction given by $k_{1j}$ and the reverse reaction given by $k_{-1j}$. The product forming reactions are not reversible with rate given by $k_{2j}$. Applying the law of mass action we obtain the ordinary differential equations

B4a $$\begin{aligned} \frac{\textrm{d}[S_j]}{\textrm{d}t}&=-k_{1j}[S_j][E_j]+k_{-1j}[SE_j], \end{aligned}$$

B4b $$\begin{aligned} \frac{\textrm{d}[E_j]}{\textrm{d}t}&=Q(\{[S_j]\})-k_{1j}[S_j][E_j]+k_{-1j}[SE_j]+k_{2j}[SE_j], \end{aligned}$$

B4c $$\begin{aligned} \frac{\textrm{d}[SE_j]}{\textrm{d}t}&=k_{1j}[S_j][E_j]-k_{-1j}[SE_j]-k_{2j}[SE_j], \end{aligned}$$

where $Q(\{[S_j]\})$ gives the rate of enzyme production and square brackets denote concentration. We assume that the enzyme-substrate complex is formed on a much faster timescale than product formation and that the concentration of complex does not change on the time-scale of product formation (quasi-steady-state assumption). We therefore set

$$\begin{aligned} \frac{\textrm{d}[SE_j]}{\textrm{d}t}&=k_{1j}[S_j][E_j]-k_{-1j}[SE_j]-k_{2j}[SE_j]=0, \end{aligned}$$

so that

B5 $$\begin{aligned}{}[SE_j]&=\frac{[S_j][E_j]}{K_{S,j}}, \end{aligned}$$

where

$$\begin{aligned} K_{S,j}=\frac{k_{-1j}+k_{2j}}{k_{1j}}, \end{aligned}$$

is the Michaelis constant for substrate *j*. We now introduce $[E_{T,j}]=[E_j]+[SE_j]$ to be the total amount of catabolic enzyme present in the system (the free enzyme plus the enzyme in the substrate-enzyme complex). Substituting in for $[SE_j]$ from Eq. (B5) and rearranging gives

$$\begin{aligned}{}[E_j]=\frac{[E_{T,j}]}{\displaystyle 1+\frac{[S_j]}{K_{S,j}}}, \end{aligned}$$

which on substitution back into Eq. (B5) gives

$$\begin{aligned}{}[SE_j]&=\frac{[S_j][E_{T,j}]}{K_{S,j}+[S_j]}. \end{aligned}$$

Equation (B4) become

$$\begin{aligned} \frac{\textrm{d}[S_j]}{\textrm{d}t}&=-k_{2j}\frac{[S_j][E_{T,j}]}{K_{S,j}+[S_j]},\\ \frac{\textrm{d}[E_{T,j}]}{\textrm{d}t}&=Q(\{[S_j]\}). \end{aligned}$$

Writing $[E_{T,j}]=p\,\Phi _{E,j}[X]$, where the constant *p* (introduced in Sect. 2.2.2) is the fraction of biomass that is protein, the substrate equations become

$$\begin{aligned} \frac{\textrm{d}[S_j]}{\textrm{d}t}&=-k_{2j}p\,\Phi _{E,j}\frac{[S_j]}{K_{S,j}+[S_j]}[X]. \end{aligned}$$

The maximum uptake rate, $k_{\textrm{max},j}$, occurs during the log-phase of growth when substrate *j* is the only substrate present and is in excess ($[S_j]\gg K_{S,j}$) so that

$$\begin{aligned} k_{\textrm{max},j}=k_{2j}p\,\Phi _{E,j}^*, \end{aligned}$$

where $\Phi _{E,j}^*$ is the value of the mass fraction of enzyme during log-phase growth on substrate *j*. It follows that

$$\begin{aligned} \frac{\textrm{d}[S_j]}{\textrm{d}t}&=-k_{\textrm{max},j}\left( \frac{\Phi _{E,j}}{\Phi _{E,j}^*}\right) \frac{[S_j]}{K_{S,j}+[S_j]}[X]. \end{aligned}$$

In terms of the rescaled mass fractions ${\bar{\Phi }}_{E,j}(=\Phi _{E,j}/\beta _{E,j})$ introduced in Appendix A.2 we have

$$\begin{aligned} \frac{\textrm{d}[S_j]}{\textrm{d}t}&=-k_{\textrm{max},j}\left( \frac{{\bar{\Phi }}_{E,j}}{{\bar{\Phi }}_{E,j}^*}\right) \frac{[S_j]}{K_{S,j}+[S_j]}[X]. \end{aligned}$$

In the main text the brackets denoting concentration are dropped: substrate and biomass concentrations in the main text are denoted by $S_j$ and *X* respectively.

## Appendix C: Solving the Flux Balance Equations to Obtain the Carbon Influxes, $J_{C,j}$

To keep the number of variables in the model to a minimum we can remove the explicit dependence of the carbon influxes, $J_{C,j}$, on *P*. In this section we solve the flux balance equations to obtain $P_{C,j}$ and $P_{A,j}$, and hence *P*, only in terms of the substrate concentrations and protein mass fractions. We can then eliminate *P* from the equation for $J_{C,j}$.

The flux balance equations, given in the main text in equation (10), are

C7 $$\begin{aligned} J_{R,j}&=\alpha _{A,j}J_{A,j}&\text { and }{} & {} J_{A,j}&=\alpha _{C,j}J_{C,j}, \end{aligned}$$

with the fluxes given by $J_{R,j}=\alpha _{A,j}k_{A,j}P_{A,j}\Phi _R$, $J_{A,j}=\alpha _{C,j}k_{C,j}P_{C,j}{\bar{\Phi }}_{\mathcal {G}}$ and, from Eq. (9),

C8 $$\begin{aligned} J_{C,j}=\left( \frac{K-P}{K}\right) \left( \frac{1}{\alpha _{A,j}\alpha _{C,j}}\right) f_j{\bar{\Phi }}_{E,j}. \end{aligned}$$

Substituting for $J_{R,j}$ and $J_{A,j}$ into the first of equations (C7) and rearranging we obtain

$$\begin{aligned} P_{A,j}=\frac{\alpha _{C,j}k_{C,j}{\bar{\Phi }}_{\mathcal {G}}}{k_{A,j}\Phi _R}P_{C,j}. \end{aligned}$$

It follows, from Eq. (7), that the combined size of precursor and amino acid pools is given by

$$\begin{aligned} P=\sum \limits _{j=1}^N\left( 1+\frac{k_{C,j}{\bar{\Phi }}_{\mathcal {G}}}{k_{A,j}\Phi _R}\right) P_{C,j}. \end{aligned}$$

This expression for *P* can now be substituted into equation (C8) from which we obtain

C9 $$\begin{aligned} J_{C,j}=\left( 1-\frac{1}{K}\sum \limits _{n=1}^N\left( 1+\frac{k_{C,n}{\bar{\Phi }}_{\mathcal {G}}}{k_{A,n}\Phi _R}\right) P_{C,n}\right) \frac{1}{\alpha _{A,j}\alpha _{C,j}}f_j{\bar{\Phi }}_{E,j}. \end{aligned}$$

Substituting for $J_{A,j}$ and $J_{C,j}$ into the second of Eq. (C7) and rearranging we obtain

$$\begin{aligned} P_{C,j}=\frac{\displaystyle \left( K-\sum \limits _{n=1(n\ne j)}^N\left( 1+\frac{\sigma _{C\textrm{max},n}{\bar{\Phi }}_{\mathcal {G}}}{\sigma _{A\textrm{max},n}\Phi _R}\right) P_{C,n}\right) f_j{\bar{\Phi }}_{E,j}}{\displaystyle \sigma _{C\textrm{max},n}{\bar{\Phi }}_{\mathcal {G}}+\left( 1+\frac{\sigma _{C\textrm{max},n}{\bar{\Phi }}_{\mathcal {G}}}{\sigma _{A\textrm{max},n}\Phi _R}\right) f_j{\bar{\Phi }}_{E,j}}. \end{aligned}$$

To simplify the notation we have introduced $\sigma _{A\textrm{max},n}=\alpha _{A,n}\alpha _{C,n}k_{A,n}K$, the maximum translation rate when only substrate *n* is being consumed. The definition of $\sigma _{A\textrm{max},n}$ follows from substituting the maximum possible value for $P_{A,n}=\alpha _{C,n}K$ (which comes from Eq. (7) with $P=K$, $P_{A,j}=0$ for $j\ne n$ and $P_{C,j}=0$ $\forall j$) into our expression for the translation rate $\sigma _A=\sum \limits _j\alpha _{A,j}k_{A,j}P_{A,j}$. We similarly define $\sigma _{C\textrm{max},n}=\alpha _{A,n}\alpha _{C,n}k_{C,n}K$ which is $\alpha _{A,n}$ times the maximum amino acid synthesis rate when only substrate *n* is being consumed. (Defining $\sigma _{C\textrm{max},n}$ as being $\alpha _{A,n}$ times the maximum amino acid synthesis rate instead of just the maximum amino acid synthesis rate further simplifies our notation.)

We now have each $P_{C,j}$ in terms of all the other $P_{C,n}$, a total of *N* equations for *N* unknowns. We solve the equations by first eliminating $P_{C,1}$ from all the equations then $P_{C,2}$, until we obtain an expression for $P_{C,N}$ which does not reference any other $P_{C,n}$. For $j=1$ we have

$$\begin{aligned}&\left( \sigma _{C\textrm{max},1}{\bar{\Phi }}_{\mathcal {G}}+\left( 1+\frac{\sigma _{C\textrm{max},1}{\bar{\Phi }}_{\mathcal {G}}}{\sigma _{A\textrm{max},1}\Phi _R}\right) f_1{\bar{\Phi }}_{E,1}\right) P_{C,1}\\&\qquad =\left( K-\left( 1+\frac{\sigma _{C\textrm{max},i}{\bar{\Phi }}_{\mathcal {G}}}{\sigma _{C\textrm{max},i}\Phi _R}\right) P_{C,i}-\sum \limits _{n=2,n\ne i}^N\left( 1+\frac{\sigma _{C\textrm{max},n}{\bar{\Phi }}_{\mathcal {G}}}{\sigma _{A\textrm{max},n}\Phi _R}\right) P_{C,n}\right) f_1{\bar{\Phi }}_{E,1}, \end{aligned}$$

and for $j=i\ge 2$ we have

$$\begin{aligned}&\left( \sigma _{C\textrm{max},i}{\bar{\Phi }}_{\mathcal {G}}+\left( 1+\frac{\sigma _{C\textrm{max},i}{\bar{\Phi }}_{\mathcal {G}}}{\sigma _{A\textrm{max},i}\Phi _R}\right) f_i{\bar{\Phi }}_{E,i}\right) P_{C,i}\\&\qquad =\left( K-\left( 1+\frac{\sigma _{C\textrm{max},1}{\bar{\Phi }}_{\mathcal {G}}}{\sigma _{A\textrm{max},1}\Phi _R}\right) P_{C,1}-\sum \limits _{n=2,n\ne i}^N\left( 1+\frac{\sigma _{C\textrm{max},n}{\bar{\Phi }}_{\mathcal {G}}}{\sigma _{A\textrm{max},n}\Phi _R}\right) P_{C,n}\right) f_i{\bar{\Phi }}_{E,i}, \end{aligned}$$

where we have written out explicitly terms in $P_{C,1}$ and $P_{C,i}$. Eliminating $P_{C,1}$ from these equations we obtain

$$\begin{aligned} P_{C,j}=\frac{\displaystyle \left( K-\sum \limits _{n=2,n\ne j}^N\left( 1+\frac{\sigma _{C\textrm{max},n}{\bar{\Phi }}_{\mathcal {G}}}{\sigma _{A\textrm{max},n}\Phi _R}\right) P_{C,n}\right) f_j{\bar{\Phi }}_{E,j}}{\displaystyle \sigma _{C\textrm{max},j}{\bar{\Phi }}_{\mathcal {G}}\left( 1+\sum \limits _{n=1,j}\left( \frac{1}{\sigma _{C\textrm{max},n}{\bar{\Phi }}_{\mathcal {G}}}+\frac{1}{\sigma _{A\textrm{max},n}\Phi _R}\right) f_n{\bar{\Phi }}_{E,n}\right) }, \end{aligned}$$

where $\sum \limits _{n=1,j}$ denotes the sum of the $1^{\textrm{st}}$ and $j{\textrm{th}}$ terms. Again, writing out explicitly terms in $j=2$ and $j=i\ge 3$ we have for $j=2$

$$\begin{aligned}&\sigma _{C\textrm{max},2}{\bar{\Phi }}_{\mathcal {G}}\left( 1+\sum \limits _{n=1}^2\left( \frac{1}{\sigma _{C\textrm{max},n}{\bar{\Phi }}_{\mathcal {G}}}+\frac{1}{\sigma _{A\textrm{max},n}\Phi _R}\right) f_n{\bar{\Phi }}_{E,n}\right) P_{C,2}\\&\qquad =\left( K-\left( 1+\frac{\sigma _{C\textrm{max},i}{\bar{\Phi }}_{\mathcal {G}}}{\sigma _{A\textrm{max},i}\Phi _R}\right) P_{C,i}-\sum \limits _{n=3,n\ne i}^N\left( 1+\frac{\sigma _{C\textrm{max},n}{\bar{\Phi }}_{\mathcal {G}}}{\sigma _{A\textrm{max},n}\Phi _R}\right) P_{C,n}\right) f_2{\bar{\Phi }}_{E,2}, \end{aligned}$$

and for $$j=i\ge 3$$

$$\begin{aligned}&\sigma _{C\textrm{max},i}{\bar{\Phi }}_{\mathcal {G}}\left( 1+\sum \limits _{n=1,i}\left( \frac{1}{\sigma _{C\textrm{max},n}{\bar{\Phi }}_{\mathcal {G}}}+\frac{1}{\sigma _{A\textrm{max},n}\Phi _R}\right) f_n{\bar{\Phi }}_{E,n}\right) P_{C,i}\\&\qquad =\left( K-\left( 1+\frac{\sigma _{C\textrm{max},2}{\bar{\Phi }}_{\mathcal {G}}}{\sigma _{A\textrm{max},2}\Phi _R}\right) P_{C,2}-\sum \limits _{n=3,n\ne i}^N\left( 1+\frac{\sigma _{C\textrm{max},n}{\bar{\Phi }}_{\mathcal {G}}}{\sigma _{A\textrm{max},n}\Phi _R}\right) P_{C,n}\right) f_i{\bar{\Phi }}_{E,i}. \end{aligned}$$

Eliminating $P_{C,2}$ from these equations we obtain

$$\begin{aligned} P_{C,j}=\frac{\displaystyle \left( K-\sum \limits _{n=3,n\ne j}^N\left( 1+\frac{\sigma _{C\textrm{max},n}{\bar{\Phi }}_{\mathcal {G}}}{\sigma _{A\textrm{max},n}\Phi _R}\right) P_{C,n}\right) f_j{\bar{\Phi }}_{E,j}}{\displaystyle \sigma _{C\textrm{max},j}{\bar{\Phi }}_{\mathcal {G}}\left( 1+\sum \limits _{n=1,2,j}\left( \frac{1}{\sigma _{C\textrm{max},n}{\bar{\Phi }}_{\mathcal {G}}}+\frac{1}{\sigma _{A\textrm{max},n}\Phi _R}\right) f_n{\bar{\Phi }}_{E,n}\right) }. \end{aligned}$$

Carrying on in this way we obtain an expression for $P_{C,N}$ as

$$\begin{aligned} P_{C,N}=\frac{\displaystyle Kf_N{\bar{\Phi }}_{E,N}}{\displaystyle \sigma _{C\textrm{max},N}{\bar{\Phi }}_{\mathcal {G}}\left( 1+\sum \limits _{n=1}^N\left( \frac{1}{\sigma _{C\textrm{max},n}{\bar{\Phi }}_{\mathcal {G}}}+\frac{1}{\sigma _{A\textrm{max},n}\Phi _R}\right) f_n{\bar{\Phi }}_{E,n}\right) }, \end{aligned}$$

and in general we have

$$\begin{aligned} P_{C,j}=\frac{\displaystyle Kf_j{\bar{\Phi }}_{E,j}}{\displaystyle \sigma _{C\textrm{max},j}{\bar{\Phi }}_{\mathcal {G}}\left( 1+\sum \limits _{n=1}^N\left( \frac{1}{\sigma _{C\textrm{max},n}{\bar{\Phi }}_{\mathcal {G}}}+\frac{1}{\sigma _{A\textrm{max},n}\Phi _R}\right) f_n{\bar{\Phi }}_{E,n}\right) }. \end{aligned}$$

Substituting into Eq. (C9) and rearranging we obtain

$$\begin{aligned} J_{C,j}&=\frac{\displaystyle \left( \frac{f_j}{\alpha _{C,j}\alpha _{A,j}}\right) {\bar{\Phi }}_{E,j}{\bar{\Phi }}_{\mathcal {G}}\Phi _R}{\displaystyle {\bar{\Phi }}_{\mathcal {G}}\Phi _R+\left( \sum \limits _{n=1}^N\frac{f_n}{\sigma _{C\textrm{max},n}}{\bar{\Phi }}_{E,n}\right) \Phi _R+\left( \sum \limits _{n=1}^N\frac{f_n}{\sigma _{A\textrm{max},n}}{\bar{\Phi }}_{E,n}\right) {\bar{\Phi }}_{\mathcal {G}}}, \end{aligned}$$

which is the carbon influx from substrate *j* in terms of only the substrate concentrations $\{S_j\}$ (through the substrate dependent functions $\{f_j\}$) and the protein mass fractions $\Phi _R$, ${\bar{\Phi }}_{\mathcal {G}}$ and ${\bar{\Phi }}_{E,j}$.

## Appendix D: Determining the Unknown Constants $\sigma _{C\textrm{max},j}$, $\sigma _{A\textrm{max},j}$ and $\alpha _{A,j}\alpha _{C,j}Y_{C,j,0}$ in Terms of Experimentally Measurable Parameters

The expression for the growth rate, given in the main text by Eq. (14), is

$$\begin{aligned} \mu =\frac{\displaystyle \left( \sum \limits _{n=1}^Nf_n{\bar{\Phi }}_{E,n}\right) {\bar{\Phi }}_{\mathcal {G}}\Phi _R}{\displaystyle {\bar{\Phi }}_{\mathcal {G}}\Phi _R+\left( \sum \limits _{n=1}^N\frac{f_n}{\sigma _{C\textrm{max},n}}{\bar{\Phi }}_{E,n}\right) \Phi _R+\left( \sum \limits _{n=1}^N\frac{f_n}{\sigma _{A\textrm{max},n}}{\bar{\Phi }}_{E,n}\right) {\bar{\Phi }}_{\mathcal {G}}}. \end{aligned}$$

This contains the unknown constants $\sigma _{C\textrm{max},j}$, $\sigma _{A\textrm{max},j}$ and $\alpha _{A,j}\alpha _{C,j}Y_{C,j,0}$, the latter combination of constants appearing in the definition of $f_j$ (Eq. (8)). We cannot determine these constants directly from known experimental measurements so instead we relate them to measurable parameters such as growth rate, biomass yield and protein mass fraction. These experimental measurements are obtained when only one substrate is present. The equation for growth rate simplifies when only one substrate is present giving

D10 $$\begin{aligned} \mu =\frac{\displaystyle f_j{\bar{\Phi }}_{E,j}{\bar{\Phi }}_{\mathcal {G}}\Phi _R}{\displaystyle {\bar{\Phi }}_{\mathcal {G}}\Phi _R+\frac{f_j}{\sigma _{C\textrm{max},j}}{\bar{\Phi }}_{E,j}\Phi _R+\frac{f_j}{\sigma _{A\textrm{max},j}}{\bar{\Phi }}_{E,j}{\bar{\Phi }}_{\mathcal {G}}}. \end{aligned}$$

The unknown constants in this equation are determined by finding the maximum value of $\mu$ in terms of the protein mass fractions and assuming that during the log-phase of growth the growth rate is equal to this maximum value.

We optimise $\mu$ in terms of $\Phi _R$, ${\bar{\Phi }}_{\mathcal {G}}$ and ${\bar{\Phi }}_{E,j}$ using the method of Lagrangian multipliers with the constraint given by

$$\begin{aligned} \Phi _{\textrm{max}}=(1+\varepsilon )\left( \Phi _R-\Phi _{R,0}\right) +\left( {\bar{\Phi }}_{\mathcal {G}}-{\bar{\Phi }}_{\mathcal {G},0}\right) +{\bar{\Phi }}_{E,j}, \end{aligned}$$

which is obtained from Eq. (2). We have used the fact that in the absence of other substrates there will be no inhibitory effects on enzyme production and therefore the mass fraction of the specific catabolic enzyme will be directly proportional to the mass fraction of the whole C-sector and, as derived in Appendix A.2, we have $\phi _C={\bar{\Phi }}_{E,j}$.

Writing

$$\begin{aligned} {\mathscr {L}}=\mu +\lambda \left( \Phi _{\textrm{max}}-(1+\varepsilon )\left( \Phi _R-\Phi _{R,0}\right) -\left( {\bar{\Phi }}_{\mathcal {G}}-{\bar{\Phi }}_{\mathcal {G},0}\right) -{\bar{\Phi }}_{E,j}\right) , \end{aligned}$$

it follows that

$$\begin{aligned} \frac{\partial {\mathscr {L}}}{\partial \Phi _R}&= \frac{\displaystyle \left( f_j\right) \left( \frac{f_j}{\sigma _{A\textrm{max},j}}\right) {\bar{\Phi }}_{\mathcal {G}}^2{\bar{\Phi }}_{E,j}^2}{\displaystyle \left( {\bar{\Phi }}_{\mathcal {G}}\Phi _R+\frac{f_j}{\sigma _{C\textrm{max},j}}{\bar{\Phi }}_{E,j}\Phi _R+\frac{f_j}{\sigma _{A\textrm{max},j}}{\bar{\Phi }}_{E,j}{\bar{\Phi }}_{\mathcal {G}}\right) ^2}-(1+\varepsilon )\lambda ,\\ \frac{\partial {\mathscr {L}}}{\partial {\bar{\Phi }}_{\mathcal {G}}}&= \frac{\displaystyle \left( f_j\right) \left( \frac{f_j}{\sigma _{C\textrm{max},j}}\right) \Phi _R^2{\bar{\Phi }}_{E,j}^2}{\displaystyle \left( {\bar{\Phi }}_{\mathcal {G}}\Phi _R+\frac{f_j}{\sigma _{C\textrm{max},j}}{\bar{\Phi }}_{E,j}\Phi _R+\frac{f_j}{\sigma _{A\textrm{max},j}}{\bar{\Phi }}_{E,j}{\bar{\Phi }}_{\mathcal {G}}\right) ^2}-\lambda ,\\ \frac{\partial {\mathscr {L}}}{\partial \phi _C}&= \frac{\displaystyle \left( f_j\right) {\bar{\Phi }}_{\mathcal {G}}^2\Phi _R^2}{\displaystyle \left( {\bar{\Phi }}_{\mathcal {G}}\Phi _R+\frac{f_j}{\sigma _{C\textrm{max},j}}{\bar{\Phi }}_{E,j}\Phi _R+\frac{f_j}{\sigma _{A\textrm{max},j}}{\bar{\Phi }}_{E,j}{\bar{\Phi }}_{\mathcal {G}}\right) ^2}-\lambda ,\\ \frac{\partial {\mathscr {L}}}{\partial \lambda }&= \Phi _{\textrm{max}}-(1+\varepsilon )\left( \Phi _R-\Phi _{R,0}\right) -\left( {\bar{\Phi }}_{\mathcal {G}}-{\bar{\Phi }}_{\mathcal {G},0}\right) -{\bar{\Phi }}_{E,j}. \end{aligned}$$

Setting all partial derivatives equal to zero and eliminating $\lambda$ we obtain

D11a $$\begin{aligned} \Phi _R^2&= {\bar{\Phi }}_{E,j}^2\left( \frac{1}{(1+\varepsilon )}\frac{f_j}{\sigma _{A\textrm{max},j}}\right) , \end{aligned}$$

D11b $$\begin{aligned} {\bar{\Phi }}_{\mathcal {G}}^2&= {\bar{\Phi }}_{E,j}^2\left( \frac{f_j}{\sigma _{C\textrm{max},j}}\right) , \end{aligned}$$

D11c $$\begin{aligned} \Phi _{\textrm{max}}&= (1+\varepsilon )\left( \Phi _R-\Phi _{R,0}\right) +\left( {\bar{\Phi }}_{\mathcal {G}}-{\bar{\Phi }}_{\mathcal {G},0}\right) +{\bar{\Phi }}_{E,j}. \end{aligned}$$

We denote experimental measurements of biomass yield and growth rate on a single substrate $S_j$ during the log-phase of growth by $Y_j^*$ and $\mu _j^*(=Y_j^*k_{\textrm{max},j})$ and mass fraction values by ${\bar{\Phi }}_{\mathcal {G},j}^*$ $\Phi _{R,j}^*$ and ${\bar{\Phi }}_{E,j}^*$. The values of the mass fractions during the log-phase are calculated from Eq. (3). Now, assuming that during log-phase the growth rate, $\mu _j^*$, takes its maximum value, the calculated values for the mass fractions must satisfy Eq. (D11) and it follows from equations (D11a) and (D11b) that

D12a $$\begin{aligned} \sigma _{A\textrm{max},j}&= \frac{f_j^*{\bar{\Phi }}_{E,j}^{*2}}{(1+\varepsilon )\Phi _{R,j}^{*2}}, \end{aligned}$$

D12b $$\begin{aligned} \sigma _{C\textrm{max},j}&= \frac{f_j^*{\bar{\Phi }}_{E,j}^{*2}}{{\bar{\Phi }}_{\mathcal {G},j}^{*2}}, \end{aligned}$$

where $f_j^*$ is the value of the function $f_j$ during the log-phase of growth on substrate *j*. From Eq. (D10) we have

$$\begin{aligned} \mu _j^*=\frac{\displaystyle f_j^*{\bar{\Phi }}_{\mathcal {G},j}^*\Phi _{R,j}^*{\bar{\Phi }}_{E,j}^*}{\displaystyle {\bar{\Phi }}_{\mathcal {G},j}^*\Phi _{R,j}^*+\left( \frac{f_j^*}{\sigma _{C\textrm{max},j}}\right) \Phi _{R,j}^*{\bar{\Phi }}_{E,j}^*+\left( \frac{f_j^*}{\sigma _{A\textrm{max},j}}\right) {\bar{\Phi }}_{\mathcal {G},j}^*{\bar{\Phi }}_{E,j}^*}. \end{aligned}$$

which after elimninating $\sigma _{A\textrm{max},j}$ and $\sigma _{C\textrm{max},j}$ using Eq. (D12) gives

$$\begin{aligned} \mu _j^*=\frac{f_j^*{\bar{\Phi }}_{E,j}^{*2}}{\Phi _{\textrm{max}}+(1+\varepsilon )\Phi _{R,0}+{\bar{\Phi }}_{\mathcal {G},0}}. \end{aligned}$$

Now $\mu _j^*=Y_j^*k_{\textrm{max},j}$ and $f_j^*=\alpha _{A,j}\alpha _{C,j}Y_{C,j,0}k_{\textrm{max},j}/{\bar{\Phi }}_{E,j}^*$, from Eq. (8), and on substituting for $\mu _j^*$ and $f_j^*$ into the above and rearranging we obtain

$$\begin{aligned} \alpha _{A,j}\alpha _{C,j}Y_{C,j,0}=\left( \frac{\Phi _{\textrm{max}}+(1+\varepsilon )\Phi _{R,0}+{\bar{\Phi }}_{\mathcal {G},0}}{{\bar{\Phi }}_{E,j}^*}\right) Y_{j}^*. \end{aligned}$$

This can now be substituted into Eq. (8) to give

D13 $$\begin{aligned} f_j(\{S_j\})=\left( \frac{\Phi _{\textrm{max}}+(1+\varepsilon )\Phi _{R,0}+{\bar{\Phi }}_{\mathcal {G},0}}{{\bar{\Phi }}_{E,j}^{*2}}\right) Y_{j}^*k_{\textrm{max},j}\frac{S_j}{K_{S,j}+S_j}, \end{aligned}$$

and we have eliminated the unknown constants from $f_j$. In addition substituting for $f_j^*=\left( \Phi _{\textrm{max}}+(1+\varepsilon )\Phi _{R,0}+{\bar{\Phi }}_{\mathcal {G},0}\right) Y_{j}^*k_{\textrm{max},j}/({\bar{\Phi }}_{E,j}^{*2})$ into Eq. (D12) we have

$$\begin{aligned} \sigma _{A\textrm{max},j}&= \frac{\left( \Phi _{\textrm{max}}+(1+\varepsilon )\Phi _{R,0}+{\bar{\Phi }}_{\mathcal {G},0}\right) Y_{j}^*k_{\textrm{max},j}}{(1+\varepsilon )\Phi _{R,j}^{*2}},\\ \sigma _{C\textrm{max},j}&= \frac{\left( \Phi _{\textrm{max}}+(1+\varepsilon )\Phi _{R,0}+{\bar{\Phi }}_{\mathcal {G},0}\right) Y_{j}^*k_{\textrm{max},j}}{{\bar{\Phi }}_{\mathcal {G},j}^{*2}}, \end{aligned}$$

so that $\sigma _{A\textrm{max},j}$ and $\sigma _{C\textrm{max},j}$ are both expressed entirely in terms of experimentally measurable parameters.

## Appendix E: Rewriting the Protein Dynamics Equations in Terms of the Growth Dependent Protein Mass Fractions

The *R*, *A* and *C* sectors of the proteome are goverened by Eq. (18)

E14a $$\begin{aligned} \frac{\textrm{d}R}{\textrm{d}t}&= \left( \Phi _{R,0}+\chi _R\right) \frac{\textrm{d}Z}{\textrm{d}t}, \end{aligned}$$

E14b $$\begin{aligned} \frac{\textrm{d}C}{\textrm{d}t}&= \left( \Phi _{C,0}+\chi _C\right) \frac{\textrm{d}Z}{\textrm{d}t}, \end{aligned}$$

E14c $$\begin{aligned} \frac{\textrm{d}A}{\textrm{d}t}&= \left( \Phi _{A,0}+\chi _A\right) \frac{\textrm{d}Z}{\textrm{d}t}. \end{aligned}$$

As described in Sect. 2.2.2 we have the following relationships between total protein concentration, *Z*, and biomass concentration, *X*, and protein concentrations, *R*, *A* and *C*, and protein mass fractions, $\Phi _R$, $\Phi _A$ and $\Phi _C$,

$$\begin{aligned} Z&=pX,&R&=pX\Phi _R,&A&=pX\Phi _A,&C&=pX\Phi _C, \end{aligned}$$

where the constant *p* is the fraction of biomass that is protein. Substituting for *R* and *Z* into Eq. (E14a) we obtain

$$\begin{aligned} X\frac{\textrm{d}\Phi _R}{\textrm{d}t}+\Phi _R\frac{\textrm{d}X}{\textrm{d}t}&= \left( \Phi _{R,0}+\chi _R\right) \frac{\textrm{d}X}{\textrm{d}t}. \end{aligned}$$

Now $\Phi _R=\Phi _{R,0}+\phi _R$, where $\phi _R$ is the growth dependent ribosomal mass fraction, and we use this expression to substitute for $\Phi _R$ into the above equation giving

$$\begin{aligned} X\frac{\textrm{d}\phi _R}{\textrm{d}t}+\left( \Phi _{R,0}+\phi _R\right) \frac{\textrm{d}X}{\textrm{d}t}&= \left( \Phi _{R,0}+\chi _R\right) \frac{\textrm{d}X}{\textrm{d}t}. \end{aligned}$$

Rearranging we obtain

$$\begin{aligned} \frac{\textrm{d}\phi _R}{\textrm{d}t}&= \left( \chi _R-\phi _R\right) \frac{1}{X}\frac{\textrm{d}X}{\textrm{d}t}, \end{aligned}$$

and as the growth rate, $\mu =(1/X)(\textrm{d}X/\textrm{d}t)$ we have

$$\begin{aligned} \frac{\textrm{d}\phi _R}{\textrm{d}t}&= \left( \chi _R-\phi _R\right) \mu . \end{aligned}$$

We use the same method to rewrite the equations for *C* and *A*, Eqs. (E14b) and (E14c), in terms of $\phi _C$ and $\phi _A$ obtaining

$$\begin{aligned} \frac{\textrm{d}\phi _C}{\textrm{d}t}&= \left( \chi _C-\phi _C\right) \mu ,\\ \frac{\textrm{d}\phi _A}{\textrm{d}t}&= \left( \chi _A-\phi _A\right) \mu . \end{aligned}$$

## Appendix F: Regulation Functions

In this section we describe the calculation of the functions $C(\{S_j\})$ and $\gamma (\{S_j\})$ which appear in the definition of the regulation functions, given in the main text in equation (25). We have

F15a $$\begin{aligned} \chi _R&= \phi _R+\frac{C(\{S_j\})}{1+\varepsilon }\,g_R\left( \{S_j\},\phi _R,\phi _C,\phi _A\right) , \end{aligned}$$

F15b $$\begin{aligned} \chi _C&= \phi _C+C(\{S_j\}) \,g_C(\{S_j\},\phi _R,\phi _C,\phi _A), \end{aligned}$$

F15c $$\begin{aligned} \chi _A&= \phi _A+C(\{S_j\})\, g_A(\{S_j\},\phi _R,\phi _C,\phi _A). \end{aligned}$$

where we have introduced

F16a $$\begin{aligned} g_R(\{S_j\},\phi _R,\phi _C,\phi _A)&= \left( \frac{1}{1+\varepsilon }\right) \frac{\partial \mu }{\partial \phi _R}-\gamma \frac{\partial \mu }{\partial \phi _C}-(1-\gamma )\frac{\partial \mu }{\partial \phi _A}, \end{aligned}$$

F16b $$\begin{aligned} g_C(\{S_j\},\phi _R,\phi _C,\phi _A)&= \frac{\partial \mu }{\partial \phi _C}-\gamma \frac{\partial \mu }{\partial \phi _A}-(1-\gamma )\left( \frac{1}{1+\varepsilon }\right) \frac{\partial \mu }{\partial \phi _R}, \end{aligned}$$

F16c $$\begin{aligned} g_A(\{S_j\},\phi _R,\phi _C,\phi _A)&= \frac{\partial \mu }{\partial \phi _A}-(1-\gamma )\frac{\partial \mu }{\partial \phi _C}-\gamma \left( \frac{1}{1+\varepsilon }\right) \frac{\partial \mu }{\partial \phi _R}. \end{aligned}$$

The regulation function for each protein sector should reach its maximum value as the corresponding growth dependent protein mass fraction tends to zero, that is $\chi _R\rightarrow \chi _{R,\textrm{max}}$ as $\phi _R\rightarrow 0$, $\chi _C\rightarrow \chi _{C,\textrm{max}}$ as $\phi _C\rightarrow 0$, $\chi _A\rightarrow \chi _{A,\textrm{max}}$ as $\phi _A\rightarrow 0$. As we must also satisfy the constraint equation (19) we have $\chi _{R,\textrm{max}}\le \Phi _{\textrm{max}}/(1+\varepsilon )$, $\chi _{C,\textrm{max}}\le \Phi _{\textrm{max}}$ and $\chi _{A,\textrm{max}}\le \Phi _{\textrm{max}}$. The function $C(\{S_j\})$ is calculated to ensure that these conditions are always satisfied, as we will now show.

The concentrations of substrates, $\{S_j\}$ (and therefore the functions $\{f_j\}$) define the growth conditions. For each set of values $\{S_j\}$ there is a maximum value of the functions $g_R$, $g_C$ and $g_A$, denoted by $g_{R,\textrm{max}}(\{S_j\})$, $g_{C,\textrm{max}}(\{S_j\})$ and $g_{A,\textrm{max}}(\{S_j\})$, which can be determined by examining the limiting values of the derivatives of the growth rate.

The growth rate, given in the main text by Eq. (14), is

$$\begin{aligned} \mu =\frac{\displaystyle \left( \sum \limits _{n=1}^Nf_n{\bar{\Phi }}_{E,n}\right) {\bar{\Phi }}_{\mathcal {G}}\Phi _R}{\displaystyle {\bar{\Phi }}_{\mathcal {G}}\Phi _R+\left( \sum \limits _{n=1}^N\frac{f_n}{\sigma _{C\textrm{max},n}}{\bar{\Phi }}_{E,n}\right) \Phi _R+\left( \sum \limits _{n=1}^N\frac{f_n}{\sigma _{A\textrm{max},n}}{\bar{\Phi }}_{E,n}\right) {\bar{\Phi }}_{\mathcal {G}}}, \end{aligned}$$

so that

$$\begin{aligned} \frac{\partial \mu }{\partial \phi _R}\left( =\frac{\partial \mu }{\partial \Phi _R}\right)&= \frac{\displaystyle \left( \sum \limits _{n=1}^Nf_n{\bar{\Phi }}_{E,n}\right) \left( \sum \limits _{n=1}^N\frac{f_n}{\sigma _{A\textrm{max},n}}{\bar{\Phi }}_{E,n}\right) {\bar{\Phi }}_{\mathcal {G}}^2}{\displaystyle \left( {\bar{\Phi }}_{\mathcal {G}}\Phi _R+\left( \sum \limits _{n=1}^N\frac{f_n}{\sigma _{C\textrm{max},n}}{\bar{\Phi }}_{E,n}\right) \Phi _R+\left( \sum \limits _{n=1}^N\frac{f_n}{\sigma _{A\textrm{max},n}}{\bar{\Phi }}_{E,n}\right) {\bar{\Phi }}_{\mathcal {G}}\right) ^2},\\ \frac{\partial \mu }{\partial \phi _A}\left( =\frac{\partial \mu }{\partial {\bar{\Phi }}_{\mathcal {G}}}\right)&= \frac{\displaystyle \left( \sum \limits _{n=1}^Nf_n{\bar{\Phi }}_{E,n}\right) \left( \sum \limits _{n=1}^N\frac{f_n}{\sigma _{C\textrm{max},n}}{\bar{\Phi }}_{E,n}\right) \Phi _R^2}{\displaystyle \left( {\bar{\Phi }}_{\mathcal {G}}\Phi _R+\left( \sum \limits _{n=1}^N\frac{f_n}{\sigma _{C\textrm{max},n}}{\bar{\Phi }}_{E,n}\right) \Phi _R+\left( \sum \limits _{n=1}^N\frac{f_n}{\sigma _{A\textrm{max},n}}{\bar{\Phi }}_{E,n}\right) {\bar{\Phi }}_{\mathcal {G}}\right) ^2},\\ \frac{\partial \mu }{\partial \phi _C}&= \sum \limits _{j=1}^N\frac{\partial \mu }{\partial {\bar{\Phi }}_{E,j}}\frac{\partial {\bar{\Phi }}_{E,j}}{\partial \phi _C}, \end{aligned}$$

with

$$\begin{aligned} \frac{\partial \mu }{\partial {\bar{\Phi }}_{E,j}}&= \frac{\displaystyle f_j{\bar{\Phi }}_{\mathcal {G}}\Phi _R}{\displaystyle {\bar{\Phi }}_{\mathcal {G}}\Phi _R+\left( \sum \limits _{n=1}^N\frac{f_n}{\sigma _{C\textrm{max},n}}{\bar{\Phi }}_{E,n}\right) \Phi _R+\left( \sum \limits _{n=1}^N\frac{f_n}{\sigma _{A\textrm{max},n}}{\bar{\Phi }}_{E,n}\right) {\bar{\Phi }}_{\mathcal {G}}}\\&-\frac{\displaystyle \left( \sum \limits _{n=1}^Nf_n{\bar{\Phi }}_{E,n}\right) {\bar{\Phi }}_{\mathcal {G}}\Phi _R\left( \frac{f_j}{\sigma _{C\textrm{max},j}}\Phi _R+\frac{f_j}{\sigma _{A\textrm{max},j}}{\bar{\Phi }}_{\mathcal {G}}\right) }{\displaystyle \left( {\bar{\Phi }}_{\mathcal {G}}\Phi _R+\left( \sum \limits _{n=1}^N\frac{f_n}{\sigma _{C\textrm{max},n}}{\bar{\Phi }}_{E,n}\right) \Phi _R+\left( \sum \limits _{n=1}^N\frac{f_n}{\sigma _{A\textrm{max},n}}{\bar{\Phi }}_{E,n}\right) {\bar{\Phi }}_{\mathcal {G}}\right) ^2}. \end{aligned}$$

### All Substrate Specific Enzymes Proportional to Catabolic Sector

If all substrate specific enzymes have expression levels proportional to the total catabolic sector, ${\bar{\Phi }}_{E,j}=\phi _C$, the partial derivatives simplifies to

F17a $$\begin{aligned} \frac{\partial \mu }{\partial \phi _R}&= \frac{\displaystyle F_1(\{S_j\})F_3(\{S_j\})\phi _C^2{\bar{\Phi }}_{\mathcal {G}}^2}{\displaystyle \left( {\bar{\Phi }}_{\mathcal {G}}\Phi _R+F_2(\{S_j\})\phi _C\Phi _R+F_3(\{S_j\})\phi _C{\bar{\Phi }}_{\mathcal {G}}\right) ^2}, \end{aligned}$$

F17b $$\begin{aligned} \frac{\partial \mu }{\partial \phi _A}&= \frac{\displaystyle F_1(\{S_j\})F_2(\{S_j\})\phi _C^2\Phi _R^2}{\displaystyle \left( {\bar{\Phi }}_{\mathcal {G}}\Phi _R+F_2(\{S_j\})\phi _C\Phi _R+F_3(\{S_j\})\phi _C{\bar{\Phi }}_{\mathcal {G}}\right) ^2}, \end{aligned}$$

F17c $$\begin{aligned} \frac{\partial \mu }{\partial \phi _C}&= \frac{\displaystyle F_1(\{S_j\}){\bar{\Phi }}_{\mathcal {G}}^2\Phi _R^2}{\displaystyle \left( {\bar{\Phi }}_{\mathcal {G}}\Phi _R+F_2(\{S_j\})\phi _C\Phi _R+F_3(\{S_j\})\phi _C{\bar{\Phi }}_{\mathcal {G}}\right) ^2}, \end{aligned}$$

where

F18 $$\begin{aligned} F_1(\{S_j\})&= \sum \limits _{n=1}^Nf_n,&F_2(\{S_j\})&= \sum \limits _{n=1}^N\frac{f_n}{\sigma _{C\textrm{max},n}},&F_3(\{S_j\})&= \sum \limits _{n=1}^N\frac{f_n}{\sigma _{A\textrm{max},n}}. \end{aligned}$$

To determine $g_{R,\textrm{max}}$, $g_{C,\textrm{max}}$ and $g_{A,\textrm{max}}$ we examine the limiting values of the above derivatives as we change $\phi _R$, $\phi _C$ and $\phi _A$.

Firstly, as $\phi _R\rightarrow 0$, with $\phi _A,\phi _C\sim \Phi _{\textrm{max}}$, we note that $\partial \mu /\partial \phi _R$ is tending towards its maximum value whereas $\partial \mu /\partial \phi _A$, and $\partial \mu /\partial \phi _C$ tend to their minimal values. We have, therefore, $\partial \mu /\partial \phi _R\gg \partial \mu /\partial \phi _C,\partial \mu /\partial \phi _A$ and hence $g_{R,\textrm{max}}$ can be determined to leading order by the maximum value of $\partial \mu /\partial \phi _R$. Substituting $\Phi _R=\Phi _{R,0}$, ${\bar{\Phi }}_{\mathcal {G}}={\bar{\Phi }}_{\mathcal {G},0}+\phi _A$ and $\phi _C=\Phi _{\textrm{max}}-\phi _A$ into Eq. (F17a) and rearranging we obtain

$$\begin{aligned} \frac{\partial \mu }{\partial \phi _R}&= \frac{\displaystyle \frac{F_1}{F_3}}{\displaystyle \left( \frac{1}{F_3}\frac{\Phi _{R,0}}{(\Phi _{\textrm{max}}-\phi _A)}+\frac{F_2}{F_3}\frac{\Phi _{R,0}}{({\bar{\Phi }}_{\mathcal {G},0}+\phi _A)}+1\right) ^2}, \end{aligned}$$

which is a function of $\phi _A$ only. The maximum value of $\partial \mu /\partial \phi _R$ can thus be found by differentiating with respect to $\phi _A$ and setting the derivative to zero.

$$\begin{aligned} \frac{\textrm{d}}{\textrm{d}\phi _A}\left( \frac{\partial \mu }{\partial \phi _R}\right)&= \frac{\displaystyle -2\frac{F_1}{F_3}\left( \frac{1}{F_3}\frac{\Phi _{R,0}}{(\Phi _{\textrm{max}}-\phi _A)^2}-\frac{F_2}{F_3}\frac{\Phi _{R,0}}{({\bar{\Phi }}_{\mathcal {G},0}+\phi _A)^2}\right) }{\displaystyle \left( \frac{1}{F_3}\frac{\Phi _{R,0}}{(\Phi _{\textrm{max}}-\phi _A)}+\frac{F_2}{F_3}\frac{\Phi _{R,0}}{({\bar{\Phi }}_{\mathcal {G},0}+\phi _A)}+1\right) ^2}=0, \end{aligned}$$

from which it follows that

F19 $$\begin{aligned} \phi _A&= \frac{\sqrt{F_2}\Phi _{\textrm{max}}-{\bar{\Phi }}_{\mathcal {G},0}}{1+\sqrt{F_2}},&\left( \phi _C=\frac{\Phi _{\textrm{max}}+{\bar{\Phi }}_{\mathcal {G},0}}{1+\sqrt{F_2}}\right) , \end{aligned}$$

where we have taken the positive square root as $0\le \phi _A\le \Phi _{\textrm{max}}$. So the maximum value of $\partial \mu /\partial \phi _R$ occurs for $\Phi _R=\Phi _{R,0}$, ${\bar{\Phi }}_{\mathcal {G}}=\sqrt{F_2}(\Phi _{\textrm{max}}+{\bar{\Phi }}_{\mathcal {G},0})/(1+\sqrt{F_2})$ and $\phi _C=(\Phi _{\textrm{max}}+{\bar{\Phi }}_{\mathcal {G},0})/(1+\sqrt{F_2})$, and it follows that

$$\begin{aligned} g_{R,\textrm{max}}&= \left( \frac{1}{1+\varepsilon }\right) \frac{\displaystyle F_1F_3(\phi _{\textrm{max}}+{\bar{\Phi }}_{\mathcal {G},0})^2}{\displaystyle \left( \Phi _{R,0}(1+\sqrt{F_2})^2+F_3(\Phi _{\textrm{max}}+{\bar{\Phi }}_{\mathcal {G},0})\right) ^2}. \end{aligned}$$

In a similar manner we find as $\phi _A\rightarrow 0$

$$\begin{aligned} g_{A,\textrm{max}}&= \frac{F_1F_2\left( \Phi _{\textrm{max}}+(1+\varepsilon )\Phi _{R,0}\right) ^2}{\left( {\bar{\Phi }}_{\mathcal {G},0}\left( 1+\sqrt{(1+\varepsilon )F_3}\right) ^2+F_2\left( \Phi _{\textrm{max}}+(1+\varepsilon )\Phi _{R,0}\right) \right) ^2}, \end{aligned}$$

for

$$\begin{aligned} \phi _C&= \frac{\Phi _{\textrm{max}}+(1+\varepsilon )\Phi _{R,0}}{1+\sqrt{(1+\varepsilon )F_3}},\\ \Phi _R&= \left( \frac{1}{1+\varepsilon }\right) \frac{\sqrt{(1+\varepsilon )F_3}\left( \Phi _{\textrm{max}}+(1+\varepsilon )\Phi _{R,0}\right) }{1+\sqrt{(1+\varepsilon )F_3}}, \end{aligned}$$

and as $$\phi _C\rightarrow 0$$

$$\begin{aligned} g_{C,\textrm{max}}&= F_1, \end{aligned}$$

for

$$\begin{aligned} \Phi _R&= \left( \frac{1}{1+\varepsilon }\right) \left( \frac{\displaystyle \Phi _{\textrm{max}}+(1+\varepsilon )\Phi _{R,0}+{\bar{\Phi }}_{\mathcal {G},0}}{\displaystyle 1+\sqrt{\frac{F_2}{(1+\varepsilon )F_3}}}\right) ,\\ {\bar{\Phi }}_{\mathcal {G}}&= \frac{\displaystyle \sqrt{\frac{F_2}{(1+\varepsilon )F_3}}\left( \Phi _{\textrm{max}}+(1+\varepsilon )\Phi _{R,0}+{\bar{\Phi }}_{\mathcal {G},0}\right) }{\displaystyle 1+\sqrt{\frac{F_2}{(1+\varepsilon )F_3}}}. \end{aligned}$$

Setting $C(\{S_j\})=\Phi _{\textrm{max}}/\max (g_{R,\textrm{max}},g_{C,\textrm{max}},g_{A,\textrm{max}})$ ensures that $\chi _{R,\textrm{max}}\le \Phi _{\textrm{max}}/(1+\varepsilon )$, $\chi _{C,\textrm{max}}\le \Phi _{\textrm{max}}$ and $\chi _{A,\textrm{max}}\le \Phi _{\textrm{max}}$ as required.

To determine the function $\gamma (\{S_j\})$ we note that $\chi _R$, $\chi _A$, $\chi _C\ge 0$ (as a simplifying assumption of this model is that protein is not destroyed). We look at the limiting case $\phi _j\rightarrow 0$ for the sector for which $g_{j,\textrm{max}}$ is largest. For example, if $g_{R,\textrm{max}}>g_{C,\textrm{max}},g_{A,\textrm{max}}$ we look at what happens as $\phi _R\rightarrow 0$. In this case $C(\{S_j\})=\Phi _{\textrm{max}}/g_{R,\textrm{max}}$ and we have $\partial \mu /\partial \phi _R\rightarrow (1+\varepsilon )g_{R,\textrm{max}}$ with the other partial derivatives negligible as $\partial \mu /\partial \phi _R\gg \partial \mu /\partial \phi _C,\partial \mu /\partial \phi _A$. Substituting into equation F15 we obtain

$$\begin{aligned} \chi _R&= \frac{\Phi _{\textrm{max}}}{1+\varepsilon },\\ \chi _C&= \frac{\Phi _{\textrm{max}}+{\bar{\Phi }}_{\mathcal {G},0}}{1+\sqrt{F_2}}-\left( 1-\gamma \right) \Phi _{\textrm{max}}=0,\\ \chi _A&= \frac{\sqrt{F_2}\Phi _{\textrm{max}}-{\bar{\Phi }}_{\mathcal {G},0}}{1+\sqrt{F_2}}-\gamma \Phi _{\textrm{max}}=0, \end{aligned}$$

where we have used the values for $\phi _A$ and $\phi _C$ given in Eq. (F19). The constraint equation (19) together with the requirement that $\chi _A$ and $\chi _C$ are positive means $\chi _A=\chi _C=0$. Solving for $\gamma$ we obtain

$$\begin{aligned} \gamma&= \frac{\sqrt{F_2}\Phi _{\textrm{max}}-{\bar{\Phi }}_{\mathcal {G},0}}{\Phi _{\textrm{max}}\left( 1+\sqrt{F_2}\right) }&\text {for}\quad g_{R,\textrm{max}}>g_{C,\textrm{max}},g_{A,\textrm{max}}. \end{aligned}$$

Similarly we have

$$\begin{aligned} \gamma&= \frac{\displaystyle \Phi _{\textrm{max}}+{\bar{\Phi }}_{\mathcal {G},0}-(1+\varepsilon )\Phi _{R,0}\sqrt{\frac{F_2}{(1+\varepsilon )F_3}}}{\displaystyle \Phi _{\textrm{max}}\left( 1+\sqrt{\frac{F_2}{(1+\varepsilon )F_3}}\right) },&\text {for}\quad g_{C,\textrm{max}}>g_{R,\textrm{max}},g_{A,\textrm{max}}, \end{aligned}$$

and

$$\begin{aligned} \gamma&= \frac{\Phi _{\textrm{max}}+(1+\varepsilon )\Phi _{R,0}}{\Phi _{\textrm{max}}\left( 1+\sqrt{(1+\varepsilon )F_3}\right) },&\text {for}\quad g_{A,\textrm{max}}>g_{R,\textrm{max}},g_{C,\textrm{max}}. \end{aligned}$$

### Substrate Specific Enzymes Non-proportional to Catabolic Sector

To illustrate the case when the substrate specific enzymes are not all proportional to $\phi _C$ we use the example of glucose–lactose diauxie. Here, the mass fraction of glucose specific enzyme is proportional to $\phi _C$, ${\bar{\Phi }}_{E,\textrm{gl}}=\phi _C$, but the mass fraction of lactose specific enzyme, ${\bar{\Phi }}_{E,\textrm{la}}$, is not. We have

$$\begin{aligned} \frac{\partial \mu }{\partial \phi _C}&= \frac{\partial \mu }{\partial {\bar{\Phi }}_{E,\textrm{gl}}}+\frac{\partial \mu }{\partial {\bar{\Phi }}_{E,\textrm{la}}}\frac{\partial {\bar{\Phi }}_{E,\textrm{la}}}{\partial \phi _C}, \end{aligned}$$

As we do not have an explicit expression for ${\bar{\Phi }}_{E,\textrm{la}}$ in terms of $\phi _C$ we calculate $\partial {\bar{\Phi }}_{E,\textrm{la}}/\partial \phi _C$ by

$$\begin{aligned} \frac{\partial {\bar{\Phi }}_{E,\textrm{la}}}{\partial \phi _C}&= \frac{\displaystyle \frac{\textrm{d} {\bar{\Phi }}_{E,\textrm{la}}}{\textrm{d}t}}{\displaystyle \frac{\textrm{d}\phi _C}{\textrm{d}t}}, \end{aligned}$$

and therefore from Eq. (20b) and equation (21) we have

$$\begin{aligned} \frac{\partial {\bar{\Phi }}_{E,\textrm{la}}}{\partial \phi _C}&= \frac{\chi _{E,\textrm{la}}-{\bar{\Phi }}_{E,\textrm{la}}}{\chi _C-\phi _C}\\&= \eta _{\textrm{la}}\left( 1-\zeta _{\textrm{la}}\right) +\frac{\eta _{\textrm{la}}\zeta _{\textrm{la}}\left( \Phi _{\textrm{max}}-\phi _C\right) +\eta _{\textrm{la}}\phi _C-{\bar{\Phi }}_{E,\textrm{la}}}{\chi _C-\phi _C}. \end{aligned}$$

For $\eta _{\textrm{la}}\zeta _{\textrm{la}}\left( \Phi _{\textrm{max}}-\phi _C\right) +\eta _{\textrm{la}}\phi _C-{\bar{\Phi }}_{E,\textrm{la}}\ne 0$ we have $\partial {\bar{\Phi }}_{E,\textrm{la}}/\partial \phi _C\rightarrow \infty$ as $\chi _C\rightarrow \phi _C$ and hence $\partial \mu /\partial \phi _C\rightarrow \infty$ as $\chi _C\rightarrow \phi _C$. It follows that when $\eta _{\textrm{la}}\zeta _{\textrm{la}}\left( \Phi _{\textrm{max}}-\phi _C\right) +\eta _{\textrm{la}}\phi _C-{\bar{\Phi }}_{E,\textrm{la}}\ne 0$ the maximum of the function $g_C$ occurs when $\chi _C\rightarrow \phi _C$ giving $g_{C,\textrm{max}}\rightarrow \infty$ and therefore we must set $C=0$ (and the other regulation functions are simply $\chi _R=\phi _R$ and $\chi _A=\phi _A$).

The case $\eta _{\textrm{la}}\zeta _{\textrm{la}}\left( \Phi _{\textrm{max}}-\phi _C\right) +\eta _{\textrm{la}}\phi _C-{\bar{\Phi }}_{E,\textrm{la}}= 0$ occurs when the lactose enzyme is either at its initial leaky level ${\bar{\phi }}_{E,\textrm{la}}=\xi \Phi _{\textrm{max}}$ (in which case $\eta _{\textrm{la}}=\xi$, $\zeta _{\textrm{la}}=1$) or when it is proportional to $\phi _C$ (in which case $\eta _{\textrm{la}}=1$, $\zeta _{\textrm{la}}=0$ and ${\bar{\phi }}_{E,\textrm{la}}=\phi _C$). In both these cases we calculate the regulation functions as described in Sect. F.1, but with the modified functions

$$\begin{aligned} F_1&= \sum \limits _{n=1}^N\eta _n\left( 1-\zeta _n\right) f_n,&F_2&= \sum \limits _{n=1}^N\frac{\eta _n\left( 1-\zeta _n\right) f_n}{\sigma _{C\textrm{max},n}},&F_3&= \sum \limits _{n=1}^N\frac{\eta _n\left( 1-\zeta _n\right) f_n}{\sigma _{A\textrm{max},n}}, \end{aligned}$$

in place of those given in equation F18, to take into account whether we have ${\bar{\phi }}_{E,\textrm{la}}=\xi \Phi _{\textrm{max}}$ or ${\bar{\phi }}_{E,\textrm{la}}=\phi _C$.

## Appendix G: Governing Equations and Parameters Used in Simulation 1

In the following section we describe the governing equations, variables and parameters used to simulate glucose–lactose diauxie in both our model and, for comparison, the model of Erickson et al. (2017). Subscripts $_{\textrm{gl}}$ and $_{\textrm{la}}$ denote parameters for growth on glucose and lactose respectively.

### Our Model

The governing equations are

$$\begin{aligned} \frac{\textrm{d}S_{\textrm{gl}}}{\textrm{d}t}&= -\left( \frac{k_{\textrm{max,gl}}}{{\bar{\Phi }}_{E,\textrm{gl}}^*}\right) \left( \frac{S_{\textrm{gl}}}{K_{S,\textrm{gl}}+S_{\textrm{gl}}}\right) {\bar{\Phi }}_{E,\textrm{gl}}X,\\ \frac{\textrm{d}S_{\textrm{la}}}{\textrm{d}t}&= -\left( \frac{k_{\textrm{max,la}}}{{\bar{\Phi }}_{E,\textrm{la}}^*}\right) \left( \frac{S_{\textrm{la}}}{K_{S,\textrm{la}}+S_{\textrm{la}}}\right) {\bar{\Phi }}_{E,\textrm{la}}X,\\ \frac{\textrm{d}\phi _{R}}{\textrm{d}t}&= \left( \chi _{R}-\phi _{R}\right) \mu ,\\ \frac{\textrm{d}{\bar{\phi }}_{E,\textrm{gl}}}{\textrm{d}t}&= \left( \chi _{C}-{\bar{\phi }}_{E,\textrm{gl}}\right) \mu ,\\ \frac{\textrm{d}{\bar{\phi }}_{E,\textrm{la}}}{\textrm{d}t}&= \left( \chi _{E,\textrm{la}}-{\bar{\phi }}_{E,\textrm{la}}\right) \mu ,\\ \frac{\textrm{d}X}{\textrm{d}t}&= \mu X. \end{aligned}$$

where

$$\begin{aligned} \mu&= \frac{\displaystyle \left( f_{\textrm{gl}}{\bar{\Phi }}_{E,\textrm{gl}}+f_{\textrm{la}}{\bar{\Phi }}_{E,\textrm{la}}\right) {\bar{\Phi }}_{\mathcal {G}}\Phi _{R}}{{\bar{\Phi }}_{\mathcal {G}}\Phi _{R}+\left( \frac{f_{\textrm{gl}}{\bar{\Phi }}_{E,\textrm{gl}}}{\sigma _{C\textrm{max},\textrm{gl}}}+\frac{f_{\textrm{la}}{\bar{\Phi }}_{E,\textrm{la}}}{\sigma _{C\textrm{max},\textrm{la}}}\right) \Phi _{R}+\left( \frac{f_{\textrm{gl}}{\bar{\Phi }}_{E,\textrm{gl}}}{\sigma _{A\textrm{max},\textrm{gl}}}+\frac{f_{\textrm{la}}{\bar{\Phi }}_{E,\textrm{la}}}{\sigma _{A\textrm{max},\textrm{la}}}\right) {\bar{\Phi }}_{\mathcal {G}}}, \end{aligned}$$

with

$$\begin{aligned} f_{\textrm{gl}}&= \left( \Phi _{\textrm{max}}+(1+\varepsilon )\Phi _{R,0}+{\bar{\Phi }}_{\mathcal {G},0}\right) Y_{\textrm{gl}}^*k_{\textrm{max},\textrm{gl}}\left( \frac{1}{{\bar{\Phi }}_{E,\textrm{gl}}^*}\right) ^2\left( \frac{S_{\textrm{gl}}}{K_{S,\textrm{gl}}+S_{\textrm{gl}}}\right) ,\\ f_{\textrm{la}}&= \left( \Phi _{\textrm{max}}+(1+\varepsilon )\Phi _{R,0}+{\bar{\Phi }}_{\mathcal {G},0}\right) Y_{\textrm{la}}^*k_{\textrm{max},\textrm{la}}\left( \frac{1}{{\bar{\Phi }}_{E,\textrm{la}}^*}\right) ^2\left( \frac{S_{\textrm{la}}}{K_{S,\textrm{la}}+S_{\textrm{la}}}\right) , \\ \Phi _{R}&= \Phi _{R,0}+\phi _{R},\\ {\bar{\Phi }}_{E,\textrm{gl}}&= {\bar{\phi }}_{E,\textrm{gl}}=\phi _{C},\\ {\bar{\Phi }}_{E,\textrm{la}}&= {\bar{\phi }}_{E,\textrm{la}},\\ {\bar{\Phi }}_{\mathcal {G}}&= {\bar{\Phi }}_{\mathcal {G},0}+\Phi _{\textrm{max}}-(1+\varepsilon )\phi _{R}-{\bar{\phi }}_{E,\textrm{gl}}, \end{aligned}$$

and the constants

$$\begin{aligned} \sigma _{A\textrm{max},\textrm{gl}}&= \frac{\left( \Phi _{\textrm{max}}+(1+\varepsilon )\Phi _{R,0}+{\bar{\Phi }}_{\mathcal {G},0}\right) Y_{\textrm{gl}}^*k_{\textrm{max},\textrm{gl}}}{(1+\varepsilon )\Phi _{R,\textrm{gl}}^{*2}},\\ \sigma _{C\textrm{max},\textrm{gl}}&= \frac{\left( \Phi _{\textrm{max}}+(1+\varepsilon )\Phi _{R,0}+{\bar{\Phi }}_{\mathcal {G},0}\right) Y_{\textrm{gl}}^*k_{\textrm{max},\textrm{gl}}}{{\bar{\Phi }}_{\mathcal {G},\textrm{gl}}^{*2}},\\ \sigma _{A\textrm{max},\textrm{la}}&= \frac{\left( \Phi _{\textrm{max}}+(1+\varepsilon )\Phi _{R,0}+{\bar{\Phi }}_{\mathcal {G},0}\right) Y_{\textrm{la}}^*k_{\textrm{max},\textrm{la}}}{(1+\varepsilon )\Phi _{R,\textrm{la}}^{*2}},\\ \sigma _{C\textrm{max},\textrm{la}}&= \frac{\left( \Phi _{\textrm{max}}+(1+\varepsilon )\Phi _{R,0}+{\bar{\Phi }}_{\mathcal {G},0}\right) Y_{\textrm{la}}^*k_{\textrm{max},\textrm{la}}}{{\bar{\Phi }}_{\mathcal {G},\textrm{la}}^{*2}}, \end{aligned}$$

with $\Phi _{R,\textrm{gl}}^*$, ${\bar{\Phi }}_{\mathcal {G},\textrm{gl}}^{*}$ and ${\bar{\Phi }}_{E,\textrm{gl}}^*$ calculated using equation (3) with $\lambda ^*=Y_{\textrm{gl}}^*k_{\textrm{max},\textrm{gl}}$, and $\Phi _{R,\textrm{la}}^*$, ${\bar{\Phi }}_{\mathcal {G},\textrm{la}}^{*}$ and ${\bar{\Phi }}_{E,\textrm{la}}^*$ calculated using equation (3) with $\lambda ^*=Y_{\textrm{la}}^*k_{\textrm{max},\textrm{la}}$.

The glucose specific enzyme will always be produced and its expression level is governed by the level of the C-sector as a whole. The lactose specific enzyme, however, will only be produced when the concentration of glucose drops sufficiently. Its expression level is therefore not proportional to that of the C-sector. The regulation functions are given by

$$\begin{aligned} \chi _{R}&= \phi _{R}+\frac{C}{1+\varepsilon }\left( \left( \frac{1}{1+\varepsilon }\right) \frac{\partial \mu }{\partial \phi _{R}}-\gamma \frac{\partial \mu }{\partial \phi _{C}}-(1-\gamma )\frac{\partial \mu }{\partial \phi _{A}}\right) ,\\ \chi _{C}&= \phi _{C}+C\left( \frac{\partial \mu }{\partial \phi _{C}}-\gamma \frac{\partial \mu }{\partial \phi _{A}}-(1-\gamma )\left( \frac{1}{1+\varepsilon }\right) \frac{\partial \mu }{\partial \phi _{R}}\right) ,\\ \chi _{E,\textrm{la}}&= \eta _{\textrm{la}}\left( \zeta _{\textrm{la}}\Phi _{\textrm{max}}+\chi _{C}\left( 1-\zeta _{\textrm{la}}\right) \right) , \end{aligned}$$

with *C* and $\gamma$ calculated as described in Appendix F.2. The point at which the lactose enzyme switches on is modelled by setting

$$\begin{aligned} \eta _{\textrm{la},1}&= \frac{K_L^2+\xi S_{\textrm{gl}}^2}{K_L^2+S_{\textrm{gl}}^2},\\ \zeta _{\textrm{la}}&= \frac{1}{2}\left( 1-\tanh \left( \frac{1}{\epsilon }\left( \frac{{\bar{\phi }}_{E,\textrm{la}}}{\phi _{C}}-\frac{1}{2}\right) \right) \right) , \end{aligned}$$

where $K_L$, $\xi$ and $\epsilon$ are constants.

**Table 4:** Parameters for *E. coli* taken from the literature

Parameter	Description	Value
$\lambda ^*_{\textrm{gl}}=Y_{\textrm{gl}}^*k_{\textrm{max},\textrm{gl}}$	Growth rate on glucose	0.91 (h$^{-1}$)$^{\textrm{a}}$
$\lambda ^*_{\textrm{la}}=Y_{\textrm{la}}^*k_{\textrm{max},\textrm{la}}$	Growth rate on lactose	0.95 (h$^{-1}$)$^{\textrm{a}}$
$\Phi _{R,0}$	Minimum ribosomal mass fraction	$0.049^{\textrm{a}}$
$\nu _R$	Fitted parameter in growth law	11.02 (h$^{-1}$)$^{\textrm{a}}$
$\lambda _C$	Fitted parameter in growth law	1.17 (h$^{-1}$)$^{\textrm{a}}$
$\xi$	Pre-expression level of lactose specific enzyme	$0.012^{\textrm{a}}$
$\Phi _{\textrm{max}}$	Maximum mass fraction of growth dependent proteins	$0.43^{\textrm{b}}$
$K_{S,\textrm{gl}}$	Half saturation constant on glucose	0.04 (gL$^{-1}$)$^{\textrm{c}}$
$K_{S,\textrm{la}}$	Half saturation constant on lactose	0.43 (gL$^{-1}$)$^{\textrm{c}}$
$\varepsilon$	Constant relating uninduced sector to R-sector	$0.91^{\textrm{d}}$

$^{\textrm{a}}$Data from Erickson et al. (2017)

$^{\textrm{b}}$Data from You et al. (2013)

$^{\textrm{c}}$Data from Doshi and Venkatesh (1998)

$^{\textrm{d}}$Calculated using data from Wu et al. (2023) (see Appendix H)

**Table 5:** Fitted parameters

Parameter	Description	Best fit value
$Y^*_{\textrm{gl}}$	Log-phase yield on glucose ((OD600 *X*)/(g/L $S_{\textrm{gl}}$))	0.24
$Y^*_{\textrm{la}}$	Log-phase yield on lactose ((OD600 *X*)/(g/L $S_{\textrm{la}}$))	0.192
$\Phi _{\mathcal {G},0}$	Minimum mass fraction of $\Phi _{G}$	$2.6\times 10^{-5}$
$K_L$	Constant in function regulating lactose uptake (g/L)	0.001
$\epsilon$	Constant in function regulating lactose uptake (g/L)	0.01

The parameters in the governing equations whose values are taken from the literature are given in Table 4. Where parameter values are given in Erickson et al. (2017) these values have been used. Fitted parameters, shown in Table 5, were determined to give a best fit to the experimental data.

For the initial conditions we have taken initial values of variables from Erickson et al. (2017): $S_{\textrm{gl},I}=0.3$ g/L, $S_{\textrm{la},I}=2.0$ g/L and $X_I=0.20$ OD600. The initial values for $\phi _R$ and $\phi _C$ are obtained by substituting for the initial growth rate ($\lambda _I=0.92$ h$^{-1}$ calculated at the end of the next section) into Eq. (3) and the initial level of the lactose specific enzyme is $\xi \Phi _{\textrm{max}}$.

### Erickson Model

A description of all parameters and variables used is given in Erickson et al. (2017). We have the following governing equations

$$\begin{aligned} \frac{\textrm{d}M}{\textrm{d}t}&= \frac{\alpha _M}{\alpha }\sigma (t)M_{\textrm{Rb}}(t),\\ \frac{\textrm{d}M_{\textrm{Rb}}}{\textrm{d}t}&= \chi _{\textrm{Rb}}(t)\sigma (t)M_{\textrm{Rb}}(t),\\ \frac{\textrm{d}M_{\textrm{Cat,gl}}}{\textrm{d}t}&= \phi _{\textrm{Cat,gl,max}}\chi _{\textrm{Cat}}(t)\sigma (t)M_{\textrm{Rb}}(t),\\ \frac{\textrm{d}M_{\textrm{Cat,la}}}{\textrm{d}t}&= h_{\textrm{la}}\chi _{\textrm{Cat}}(t)\sigma (t)M_{\textrm{Rb}}(t), \end{aligned}$$

with

$$\begin{aligned} \sigma (t)&= \frac{1}{M_{\textrm{Rb}}(t)}\alpha \Big (k_{\textrm{max,gl}}\left( \frac{S_{\textrm{gl}}(t)}{K_{M,\textrm{gl}}+S_{\textrm{gl}}(t)}\right) M_{\textrm{Cat,gl}}(t)+k_{\textrm{max,la}}M_{\textrm{Cat,la}}(t)\Big ),\\ \chi _{\textrm{Rb}}(t)&= \frac{\phi _{\textrm{Rb},0}}{1-\sigma (t)/\gamma },\\ \chi _{\textrm{Cat}}(t)&= 1-\frac{\sigma (t)}{\lambda _C}\chi _{\textrm{Rb}}(t),\\ h_{\textrm{la}}(t)&= {\left\{ \begin{array}{ll} x\phi _{\textrm{Cat,la,max}} &{} \text {for }t<t_{sw},\\ \phi _{\textrm{Cat,la,max}} &{} \text {for } t\ge t_{sw}, \end{array}\right. }. \end{aligned}$$

From the equations during log phase growth on a single substrate the relation $\alpha k_{\textrm{max},j}=\lambda ^*_j/\phi _{\textrm{Cat},j}^*$ is obtained, where $\lambda ^*_j$ is the log phase growth rate on the substrate and $\phi _{\textrm{Cat},j}^*$ is the value of the mass fraction during log phase growth. The equation for $\sigma (t)$ becomes

$$\begin{aligned} \sigma (t)&= \frac{1}{M_{\textrm{Rb}}(t)}\left( \frac{\lambda ^*_{\textrm{gl}}}{\phi _{\textrm{Cat,gl,max}}(1-\lambda ^*_{\textrm{gl}}/\lambda _C)}\left( \frac{S_{\textrm{gl}}}{K_{M,\textrm{gl}}+S_{\textrm{gl}}}\right) M_{\textrm{Cat,gl}}(t)\right. \\&\left. \qquad \qquad +\frac{\lambda ^*_{\textrm{la}}}{\phi _{\textrm{Cat,la,max}}(1-\lambda ^*_{\textrm{la}}/\lambda _C)}M_{\textrm{Cat,la}}(t)\right) , \end{aligned}$$

where we have substituted in for $\phi ^*_{\textrm{Cat,gl}}=\phi _{\textrm{Cat,gl,max}}(1-\lambda ^*_{\textrm{gl}}/\lambda _C)$ and $\phi ^*_{\textrm{Cat,la}}=\phi _{\textrm{Cat,la,max}}(1-\lambda ^*_{\textrm{la}}/\lambda _C)$, from the growth laws. To tidy up the equations we rescale with ${\bar{M}}_{\textrm{Cat,gl}}=\alpha _M M_{\textrm{Cat,gl}}/(\alpha \phi _{\textrm{Cat,gl,max}})$, ${\bar{M}}_{\textrm{Cat,la}}=\alpha _M M_{\textrm{Cat,la}}/(\alpha \phi _{\textrm{Cat,la,max}})$ and ${\bar{M}}_{\textrm{Rb}}=\alpha _M M_{\textrm{Rb}}/\alpha$ to give

$$\begin{aligned} \frac{\textrm{d}M}{\textrm{d}t}&= \sigma (t){\bar{M}}_{\textrm{Rb}}(t),\\ \frac{\textrm{d}{\bar{M}}_{\textrm{Rb}}}{\textrm{d}t}&= \chi _{\textrm{Rb}}(t)\sigma (t){\bar{M}}_{\textrm{Rb}}(t),\\ \frac{\textrm{d}{\bar{M}}_{\textrm{Cat,gl}}}{\textrm{d}t}&= \chi _{\textrm{Cat}}(t)\sigma (t){\bar{M}}_{\textrm{Rb}}(t),\\ \frac{\textrm{d}{\bar{M}}_{\textrm{Cat,la}}}{\textrm{d}t}&= {\bar{h}}_{\textrm{la}}\chi _{\textrm{Cat}}(t)\sigma (t){\bar{M}}_{\textrm{Rb}}(t), \end{aligned}$$

with

$$\begin{aligned} \sigma (t)&= \frac{1}{{\bar{M}}_{\textrm{Rb}}(t)}\left( \frac{\lambda ^*_{\textrm{gl}}}{(1-\lambda ^*_{\textrm{gl}}/\lambda _C)}\left( \frac{S_{\textrm{gl}}}{K_{M,\textrm{gl}}+S_{\textrm{gl}}}\right) {\bar{M}}_{\textrm{Cat,gl}}(t)+\frac{\lambda ^*_{\textrm{la}}}{(1-\lambda ^*_{\textrm{la}}/\lambda _C)}{\bar{M}}_{\textrm{Cat,la}}(t)\right) ,\\ \chi _{\textrm{Rb}}(t)&= \frac{\phi _{\textrm{Rb},0}}{1-\sigma (t)/\gamma },\\ \chi _{\textrm{Cat}}(t)&= 1-\frac{\sigma (t)}{\lambda _C}\chi _{\textrm{Rb}}(t),\\ {\bar{h}}_{\textrm{la}}(t)&= {\left\{ \begin{array}{ll} x &{} \text {for }t<t_{sw},\\ 1 &{} \text {for } t\ge t_{sw}, \end{array}\right. }. \end{aligned}$$

This system of equations is not closed as it does not include an equation for $S_{\textrm{gl}}$. This equation is not given explicitly in Erickson et al. (2017) but we take it to be

$$\begin{aligned} \frac{\textrm{d}S_{\textrm{gl}}}{\textrm{d}t}&= -\frac{k_{\textrm{max},j}}{\alpha _S}\left( \frac{S_{\textrm{gl}}}{K_{M,\textrm{gl}}+S_{\textrm{gl}}}\right) M_{\textrm{Cat,gl}}. \end{aligned}$$

We have introduced the constant $\alpha _S$ to represent the conversion factor from imported substrate to metabolic influx because in Erickson et al. (2017) this conversion factor has been incorporated into the uptake rate ($J_C=k_{\textrm{gl}}M_{\textrm{Cat,gl}}+k_{\textrm{la}}M_{\textrm{Cat,la}}$). In terms of the rescaled protein concentration we have

$$\begin{aligned} \frac{\textrm{d}S_{\textrm{gl}}}{\textrm{d}t}&= -\frac{\lambda ^*_{\textrm{gl}}}{(1-\lambda ^*_{\textrm{gl}}/\lambda _C)}\frac{1}{\alpha _M\alpha _S}\left( \frac{S_{\textrm{gl}}}{K_{M,\textrm{gl}}+S_{\textrm{gl}}}\right) {\bar{M}}_{\textrm{Cat,gl}}. \end{aligned}$$

Now $\alpha _M$ is the conversion factor from carbon influx to biomass flux so the product $\alpha _M\alpha _S$ gives the conversion from imported substrate to biomass flux (the yield of biomass). The yield is assumed to be a substrate specific constant but its value is not given in Erickson et al. (2017). We, therefore, fit the yield to match the results shown in Erickson et al. (2017).

The initial values of biomass and glucose are taken from Erickson et al. (2017), $S_{\textrm{gl},I}=0.3$ g/L and $M_I=0.20$ OD600 and for the proteins we have

$$\begin{aligned} {\bar{M}}_{\textrm{Rb},I}&= \left( \phi _{\textrm{Rb},0}+\frac{\lambda _I}{\gamma }\right) M_I,\\ {\bar{M}}_{\textrm{Cat,gl},I}&= \left( 1-\frac{\lambda _I}{\lambda _C}\right) M_I,\\ {\bar{M}}_{\textrm{Cat,la},I}&= x\left( 1-\frac{\lambda _I}{\lambda _C}\right) M_I. \end{aligned}$$

To find initial growth rate $\lambda _I$ we know that $\textrm{d}M/\textrm{d}t=\lambda (t)M(t)$ so it follows that $\sigma (t){\bar{M}}_{\textrm{Rb}}(t)=\lambda (t)M(t)$. As $M_{\textrm{Rb}}(t)=\phi _{\textrm{Rb}}(t)M_P(t)$, where $M_P(t)=(\alpha /\alpha _M)M(t)$ is the total protein concentration, we have ${\bar{M}}_{\textrm{Rb}}(t)=\phi _{\textrm{Rb}}(t)M(t)$. The growth rate is therefore given by $\lambda (t)=\sigma (t)\phi _{\textrm{Rb}}(t)$. If we also note that ${\bar{M}}_{\textrm{Cat,gl}}(t)=(\phi _{\textrm{Cat,gl}}(t)/\phi _{\textrm{Cat,gl,max}})M(t)$ and ${\bar{M}}_{\textrm{Cat,la}}(t)=(\phi _{\textrm{Cat,la}}(t)/\phi _{\textrm{Cat,la,max}})M(t)$ we obtain

$$\begin{aligned} \lambda (t)&= \frac{\lambda ^*_{\textrm{gl}}}{(1-\lambda ^*_{\textrm{gl}}/\lambda _C)}\left( \frac{S_{\textrm{gl}}(t)}{K_{M,\textrm{gl}}+S_{\textrm{gl}}(t)}\right) \frac{\phi _{\textrm{Cat,gl}}(t)}{\phi _{\textrm{Cat,gl,max}}}+\frac{\lambda ^*_{\textrm{la}}}{(1-\lambda ^*_{\textrm{la}}/\lambda _C)}\frac{\phi _{\textrm{Cat,la}}(t)}{\phi _{\textrm{Cat,la,max}}}. \end{aligned}$$

Now $S_{\textrm{gl},I}\gg K_{M,\textrm{gl}}$ so we have

$$\begin{aligned} \lambda _I&= \frac{\lambda ^*_{\textrm{gl}}}{(1-\lambda ^*_{\textrm{gl}}/\lambda _C)}\frac{\phi _{\textrm{Cat,gl},I}}{\phi _{\textrm{Cat,gl,max}}}+\frac{\lambda ^*_{\textrm{la}}}{(1-\lambda ^*_{\textrm{la}}/\lambda _C)}\frac{\phi _{\textrm{Cat,la},I}}{\phi _{\textrm{Cat,la,max}}}, \end{aligned}$$

and if we assume that initially everything is in steady state we can write $\phi _{\textrm{Cat,gl},I}=\phi _{\textrm{Cat,gl,max}}(1-\lambda _I/\lambda _C)$ and $\phi _{\textrm{Cat,la},I}=x\phi _{\textrm{Cat,la,max}}(1-\lambda _I/\lambda _C)$ (the *x* appears here as the lactose metabolism is not yet switched on) giving

$$\begin{aligned} \lambda _I&= \frac{\lambda ^*_{\textrm{gl}}}{(1-\lambda ^*_{\textrm{gl}}/\lambda _C)}(1-\lambda _I/\lambda _C)+\frac{\lambda ^*_{\textrm{la}}}{(1-\lambda ^*_{\textrm{la}}/\lambda _C)}x(1-\lambda _I/\lambda _C). \end{aligned}$$

This can be solved to give

$$\begin{aligned} \lambda _I&= \frac{\lambda ^*_{\textrm{gl}}+x\lambda ^*_{\textrm{la}}-(1+x)\frac{\lambda ^*_{\textrm{gl}}\lambda ^*_{\textrm{la}}}{\lambda _C}}{1-(1-x)\frac{\lambda ^*_{\textrm{la}}}{\lambda _C}-x\frac{\lambda ^*_{\textrm{gl}}\lambda ^*_{\textrm{la}}}{\lambda _C^2}}. \end{aligned}$$

## Appendix H: Governing Equations and Parameters Used in Simulation 2

In the following governing equations, subscripts $_1$ and $_2$ denote the mass fractions and growth rates of strains $X_1$ and $X_2$ respectively. Subscripts $_{\textrm{gl}}$ and $_{\textrm{la}}$ denote parameters for growth on glucose and lactose respectively. The two strains are assumed to have the same parameters for growth on glucose. For both strains the glucose specific enzyme will always be produced and its expression level is governed by the level of the C-sector as a whole. The lactose specific enzyme, however, is never produced by the mutant strain $X_2$ and will only be produced by $X_1$ when the concentration of glucose drops sufficiently. Its expression level is not proportional to that of the C-sector. Our governing equations are

$$\begin{aligned} \frac{\textrm{d}S_{\textrm{gl}}}{\textrm{d}t}&= -\left( \frac{k_{\textrm{max,gl}}}{{\bar{\Phi }}_{E,\textrm{gl}}^*}\right) \left( \frac{S_{\textrm{gl}}}{K_{S,\textrm{gl}}+S_{\textrm{gl}}}\right) \left( {\bar{\Phi }}_{E,\textrm{gl},1}X_1+{\bar{\Phi }}_{E,\textrm{gl},2}X_2\right) ,\\ \frac{\textrm{d}S_{\textrm{la}}}{\textrm{d}t}&= -\left( \frac{k_{\textrm{max,la}}}{{\bar{\Phi }}_{E,\textrm{la}}^*}\right) \left( \frac{S_{\textrm{la}}}{K_{S,\textrm{la}}+S_{\textrm{la}}}\right) {\bar{\Phi }}_{E,\textrm{la},1}X_1, \\ \frac{\textrm{d}\phi _{R,1}}{\textrm{d}t}&= \left( \chi _{R,1}-\phi _{R,1}\right) \mu _1, \quad \frac{\textrm{d}\phi _{R,2}}{\textrm{d}t} = \left( \chi _{R,2}-\phi _{R,2}\right) \mu _2,\\ \frac{\textrm{d}{\bar{\phi }}_{E,\textrm{gl},1}}{\textrm{d}t}&= \left( \chi _{C,1}-{\bar{\phi }}_{E,\textrm{gl},1}\right) \mu _1, \quad \frac{\textrm{d}{\bar{\phi }}_{E,\textrm{gl},2}}{\textrm{d}t} = \left( \chi _{C,2}-{\bar{\phi }}_{E,\textrm{gl},2}\right) \mu _2,\\ \frac{\textrm{d}{\bar{\phi }}_{E,\textrm{la},1}}{\textrm{d}t}&= \left( \chi _{E,\textrm{la},1}-{\bar{\phi }}_{E,\textrm{la},1}\right) \mu _1, \quad {\bar{\phi }}_{E,\textrm{la},2} = 0,\\ \frac{\textrm{d}X_1}{\textrm{d}t}&= \mu _1 X_1, \quad \frac{\textrm{d}X_2}{\textrm{d}t} = \mu _2 X_2. \end{aligned}$$

where

$$\begin{aligned} \mu _1&= \frac{\displaystyle \left( f_{\textrm{gl}}{\bar{\Phi }}_{E,\textrm{gl},1}+f_{\textrm{la}}{\bar{\Phi }}_{E,\textrm{la},1}\right) {\bar{\Phi }}_{\mathcal {G},1}\Phi _{R,1}}{{\bar{\Phi }}_{\mathcal {G},1}\Phi _{R,1}+\left( \frac{f_{\textrm{gl}}{\bar{\Phi }}_{E,\textrm{gl},1}}{\sigma _{C\textrm{max},\textrm{gl}}}+\frac{f_{\textrm{la}}{\bar{\Phi }}_{E,\textrm{la},1}}{\sigma _{C\textrm{max},\textrm{la}}}\right) \Phi _{R,1}+\left( \frac{f_{\textrm{gl}}{\bar{\Phi }}_{E,\textrm{gl},1}}{\sigma _{A\textrm{max},\textrm{gl}}}+\frac{f_{\textrm{la}}{\bar{\Phi }}_{E,\textrm{la},1}}{\sigma _{A\textrm{max},\textrm{la}}}\right) {\bar{\Phi }}_{\mathcal {G},1}},\\ \mu _2&= \frac{\displaystyle \left( f_{\textrm{gl}}{\bar{\Phi }}_{E,\textrm{gl},2}\right) {\bar{\Phi }}_{\mathcal {G},2}\Phi _{R,2}}{\displaystyle {\bar{\Phi }}_{\mathcal {G},2}\Phi _{R,2}+\left( \frac{f_{\textrm{gl}}{\bar{\Phi }}_{E,\textrm{gl},2}}{\sigma _{C\textrm{max},\textrm{gl}}}\right) \Phi _{R,2}+\left( \frac{f_{\textrm{gl}}{\bar{\Phi }}_{E,\textrm{gl},2}}{\sigma _{A\textrm{max},\textrm{gl}}}\right) {\bar{\Phi }}_{\mathcal {G},2}}, \end{aligned}$$

with

$$\begin{aligned} f_{\textrm{gl}}&= \left( \Phi _{\textrm{max}}+(1+\varepsilon )\Phi _{R,0}+{\bar{\Phi }}_{\mathcal {G},0}\right) Y_{\textrm{gl}}^*k_{\textrm{max},\textrm{gl}}\left( \frac{1}{{\bar{\Phi }}_{E,\textrm{gl}}^*}\right) ^2\left( \frac{S_{\textrm{gl}}}{K_{S,\textrm{gl}}+S_{\textrm{gl}}}\right) ,\\ f_{\textrm{la}}&= \left( \Phi _{\textrm{max}}+(1+\varepsilon )\Phi _{R,0}+{\bar{\Phi }}_{\mathcal {G},0}\right) Y_{\textrm{la}}^*k_{\textrm{max},\textrm{la}}\left( \frac{1}{{\bar{\Phi }}_{E,\textrm{la}}^*}\right) ^2\left( \frac{S_{\textrm{la}}}{K_{S,\textrm{la}}+S_{\textrm{la}}}\right) , \\ \Phi _{R,1}&= \Phi _{R,0}+\phi _{R,1}, \quad \Phi _{R,2} = \Phi _{R,0}+\phi _{R,2},\\ {\bar{\Phi }}_{E,\textrm{gl},1}&= {\bar{\phi }}_{E,\textrm{gl},1}=\phi _{C,1}, \quad {\bar{\Phi }}_{E,\textrm{gl},2} = {\bar{\phi }}_{E,\textrm{gl},2}=\phi _{C,2},\\ {\bar{\Phi }}_{E,\textrm{la},1}&= {\bar{\phi }}_{E,\textrm{la},1},\quad {\bar{\Phi }}_{E,\textrm{la},2} = {\bar{\phi }}_{E,\textrm{la},2}=0,\\ {\bar{\Phi }}_{\mathcal {G},1}&= {\bar{\Phi }}_{\mathcal {G},0}+\Phi _{\textrm{max}}-(1+\varepsilon )\phi _{R,1}-{\bar{\phi }}_{E,\textrm{gl},1},\\ {\bar{\Phi }}_{\mathcal {G},2}&= {\bar{\Phi }}_{\mathcal {G},0}+\Phi _{\textrm{max}}-(1+\varepsilon )\phi _{R,2}-{\bar{\phi }}_{E,\textrm{gl},2}, \end{aligned}$$

and the constants

$$\begin{aligned} \sigma _{A\textrm{max},\textrm{gl}}&= \frac{\left( \Phi _{\textrm{max}}+(1+\varepsilon )\Phi _{R,0}+{\bar{\Phi }}_{\mathcal {G},0}\right) Y_{\textrm{gl}}^*k_{\textrm{max},\textrm{gl}}}{(1+\varepsilon )\Phi _{R,\textrm{gl}}^{*2}},\\ \sigma _{C\textrm{max},\textrm{gl}}&= \frac{\left( \Phi _{\textrm{max}}+(1+\varepsilon )\Phi _{R,0}+{\bar{\Phi }}_{\mathcal {G},0}\right) Y_{\textrm{gl}}^*k_{\textrm{max},\textrm{gl}}}{{\bar{\Phi }}_{\mathcal {G},\textrm{gl}}^{*2}},\\ \sigma _{A\textrm{max},\textrm{la}}&= \frac{\left( \Phi _{\textrm{max}}+(1+\varepsilon )\Phi _{R,0}+{\bar{\Phi }}_{\mathcal {G},0}\right) Y_{\textrm{la}}^*k_{\textrm{max},\textrm{la}}}{(1+\varepsilon )\Phi _{R,\textrm{la}}^{*2}},\\ \sigma _{C\textrm{max},\textrm{la}}&= \frac{\left( \Phi _{\textrm{max}}+(1+\varepsilon )\Phi _{R,0}+{\bar{\Phi }}_{\mathcal {G},0}\right) Y_{\textrm{la}}^*k_{\textrm{max},\textrm{la}}}{{\bar{\Phi }}_{\mathcal {G},\textrm{la}}^{*2}}. \end{aligned}$$

The regulation functions are given by

$$\begin{aligned} \chi _{R,1}&= \phi _{R,1}+\frac{C_1}{1+\varepsilon }\left( \left( \frac{1}{1+\varepsilon }\right) \frac{\partial \mu _1}{\partial \phi _{R,1}}-\gamma _1\frac{\partial \mu _1}{\partial \phi _{C,1}}-(1-\gamma _1)\frac{\partial \mu _1}{\partial \phi _{A,1}}\right) ,\\ \chi _{C,1}&= \phi _{C,1}+C_1\left( \frac{\partial \mu _1}{\partial \phi _{C,1}}-\gamma _1\frac{\partial \mu _1}{\partial \phi _{A,1}}-(1-\gamma _1)\left( \frac{1}{1+\varepsilon }\right) \frac{\partial \mu _1}{\partial \phi _{R,1}}\right) ,\\ \chi _{E,\textrm{la},1}&= \eta _{\textrm{la}}\left( \zeta _{\textrm{la}}\Phi _{\textrm{max}}+\chi _{C,1}\left( 1-\zeta _{\textrm{la}}\right) \right) ,\\ \chi _{R,2}&= \phi _{R,2}+\frac{C_2}{1+\varepsilon }\left( \left( \frac{1}{1+\varepsilon }\right) \frac{\partial \mu _2}{\partial \phi _{R,2}}-\gamma _1\frac{\partial \mu _2}{\partial \phi _{C,2}}-(1-\gamma _1)\frac{\partial \mu _2}{\partial \phi _{A,2}}\right) ,\\ \chi _{C,2}&= \phi _{C,2}+C_2\left( \frac{\partial \mu _2}{\partial \phi _{C,2}}-\gamma _2\frac{\partial \mu _2}{\partial \phi _{A,2}}-(1-\gamma _2)\left( \frac{1}{1+\varepsilon }\right) \frac{\partial \mu _2}{\partial \phi _{R,2}}\right) , \end{aligned}$$

with $C_1$, $C_2$, $\gamma _1$ and $\gamma _2$ calculated as described in Appendix F.2. The point at which the lactose enzyme switches on is modelled by setting

$$\begin{aligned} \eta _{\textrm{la}}&= \frac{K_L^2+\xi S_{\textrm{gl}}^2}{K_L^2+S_{\textrm{gl}}^2},\\ \zeta _{\textrm{la}}&= \frac{1}{2}\left( 1-\tanh \left( \frac{1}{\epsilon }\left( \frac{{\bar{\phi }}_{E,\textrm{la},1}}{\phi _{C,1}}-\frac{1}{2}\right) \right) \right) , \end{aligned}$$

where $K_L$, $\xi$ and $\epsilon$ are constants.

We keep the parameter values the same as in the diauxic-shift only simulation (described in Appendix G with parameters shown in Tables 4 and 5) with the exception of the log-phase yields, $Y^*_{\textrm{gl}}$ and $Y^*_{\textrm{la}}$ (fitted values for simulation 2 are $Y^*_{\textrm{gl}}=0.67$ and $Y^*_{\textrm{la}}=0.536$). This is because the yield depends on the ratio of OD600 to g/L of biomass which will differ between the experiments. We do, however, keep the ratio $Y^*_{\textrm{gl}}:Y^*_{\textrm{la}}$ the same in both simulations.

Initial values for $S_{\textrm{gl}}$, $S_{\textrm{la}}$ and $X_1=X_2$ were taken from our experimental data at $t=0$. The initial value for $\phi _{R,1}=\phi _{R,2}=0.2$ was determined using data from Wu et al. (2023) assuming a doubling rate in Luria–Bertani broth of 25 min (Tao et al. 1999). The initial level of catabolic proteins was taken to be ${\bar{\phi }}_{E,\textrm{gl},1}={\bar{\phi }}_{E,\textrm{gl},2}={\bar{\Phi }}_{E,\textrm{gl}}^*/2$. This is based on data from Wu et al. (2023) showing that the expression level of glycerol uptake enzymes approximately doubles in the transition from rich to minimal media. The anabolic proteins were initially assumed to be at their minimum value so $\phi _{A,1}=\phi _{A,2}=0$. Using the initial levels of the protein mass fractions and the constraint equation 2 we obtain the value of the constant relating the uninduced sector to the ribosomal sector as $\varepsilon = 0.91$.
